# Imaging high-frequency voltage dynamics in multiple neuron classes of behaving mammals

**DOI:** 10.1016/j.cell.2025.06.028

**Published:** 2025-07-16

**Authors:** Simon Haziza, Radosław Chrapkiewicz, Yanping Zhang, Vasily Kruzhilin, Jane Li, Jizhou Li, Geoffroy Delamare, Rachel Swanson, György Buzsáki, Madhuvanthi Kannan, Ganesh Vasan, Michael Z. Lin, Hongkui Zeng, Tanya L. Daigle, Mark J. Schnitzer

**Affiliations:** 1 James H. Clark Center, Stanford University, Stanford, CA 94305, USA; 2 CNC Program, Stanford University, Stanford, CA 94305, USA; 3 Department of Biology, Stanford University, Stanford, CA 94305, USA; 4 Howard Hughes Medical Institute, Stanford University, Stanford, CA 94305, USA; 5 Department of Applied Physics, Stanford University, Stanford, CA 94305, USA; 6 Neuroscience Institute, Langone Medical Center, New York University, New York, NY 10016, USA; 7 Department of Neurology, Langone Medical Center, New York University, New York, NY 10016, USA; 8 Department of Neuroscience, University of Minnesota, Minneapolis, MN 55455, USA; 9 Departments of Bioengineering & Neurobiology, Stanford University, Stanford, CA 94305, USA; 10 Allen Institute for Brain Science, Seattle, WA 98109, USA; 11 Department of Neurosurgery, Stanford University, Stanford, CA 94305, USA

## Abstract

Fluorescent genetically encoded voltage indicators report transmembrane potentials of targeted cell types. However, voltage-imaging instrumentation has lacked the sensitivity to track spontaneous or evoked high-frequency voltage oscillations in neural populations. Here, we describe two complementary TEMPO (transmembrane electrical measurements performed optically) voltage-sensing technologies that capture neural oscillations up to ~100 Hz. Fiber-optic TEMPO achieves ~10-fold greater sensitivity than prior photometric voltage sensing, allows hour-long recordings, and monitors two neuron classes per fiber-optic probe in freely moving mice. With it, we uncovered cross-frequency-coupled theta- and gamma-range oscillations and characterized excitatory-inhibitory neural dynamics during hippocampal ripples and visual cortical processing. The TEMPO mesoscope images voltage activity in two cell classes across an ~8-mm-wide field of view in head-fixed animals. In awake mice, it revealed sensory-evoked excitatory-inhibitory neural interactions and traveling gamma and 3–7 Hz waves in visual cortex and bidirectional propagation directions for both hippocampal theta and beta waves. These technologies have widespread applications probing diverse oscillations and neuron-type interactions in healthy and diseased brains.

## In brief

TEMPO optical voltage sensing in fiber-optic and mesoscope imaging formats captures transmembrane voltage dynamics, oscillations, and waves in targeted neuron types of freely moving and head-fixed behaving animals. These techniques reveal couplings between oscillations of distinct frequencies in specific neuron classes and bidirectional propagation directions for both hippocampal theta and beta waves.

## Graphical Abstract

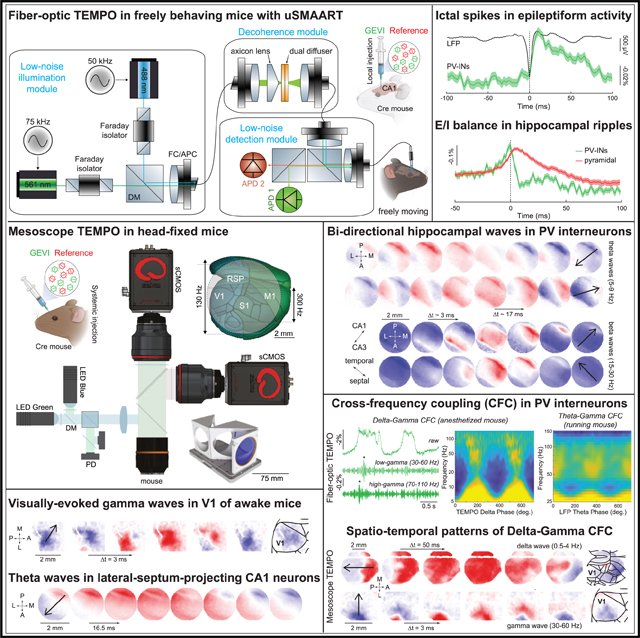

## INTRODUCTION

Fluorescence imaging studies of neural activity using genetically encoded voltage indicators (GEVIs) have generally focused on detecting neural action potentials.^1–6^ However, fiber-optic photometry studies of subthreshold voltage dynamics have revealed collective activity in genetically identified neuron types in behaving rodents,^7–9^ and population-level voltage-imaging studies have mapped sensory-evoked potentials or slow oscillations in head-fixed animals.^10–13^ Nonetheless, prior optical voltage-sensing studies in freely moving or head-restrained rodents lacked the sensitivity to detect oscillations above theta (~5–9 Hz) frequencies without substantial trial averaging.^7,9,13^

Creating techniques to image high-frequency (⪆10 Hz) oscillations is crucial, as oscillations such as beta (~15–30 Hz) and gamma (~35–100 Hz) rhythms are broadly implicated in attention,^14,15^ motor control,^16,17^ memory^18–21^ and sensory processing,^22,23^ and impaired in cognitive disorders,^24^ schizophrenia,^25^ epilepsy,^26–28^ and Alzheimer’s^29–31^ and Parkinson’s diseases.^32–34^ However, much work remains to clarify how different neuron types and their interactions shape rhythmic synchronization.^9,14,21,35–37^

For dissections of how different neuron classes influence high-frequency oscillations, we developed the TEMPO (transmembrane electrical measurements performed optically) approach, demonstrated previously in fiber-optics.^7^ TEMPO captures aggregate dynamics of specific neuron types, revealing collective activity that may be indiscernible from single-cell recordings. TEMPO has 4 ingredients: (1) one or more GEVIs to track activity in specific neuron types, targeted with viral or transgenic strategies; (2) a voltage-insensitive reference fluor to track hemodynamic, brain motion, or other optical artifacts; (3) a dual-color fluorescence measurement apparatus; and (4) computational unmixing of artifactual and neural voltage signals.

Notably, TEMPO and extracellular local field potential (LFP) recordings track neural activity differently. LFPs reflect contributions of multiple cell types, are influenced by electrode shape, orientation and composition, and comprise time-varying, unknown mixtures of signals across length scales—from currents ~0.1 mm from the electrode to volume-conducted signals ≤1 cm away.^38^ TEMPO probes intracellular potentials of user-selected cell types, free from volume conduction.

Here, we present two complementary TEMPO instruments, for fiber-optic voltage-sensing and wide-field voltage imaging. Both achieve the sensitivity needed to track high-frequency oscillations without trial-averaging and use a convolutional filtering algorithm to unmix artifacts from voltage signals. While we usually applied viral expression strategies, our TEMPO reporter mouse line enables highly reproducible, dual-fluor labeling without virus use.

Our fiber-optic apparatus, uSMAART (ultra-sensitive measurement of aggregate activity in restricted cell types) is an ultra-sensitive instrument with neuroscience applications beyond voltage sensing. Through dual-color detection, uSMAART tracks one or two neuron classes in each of 1–2 brain areas concurrently. Its sensitivity is ~10-fold greater than prior fiber photometry systems for voltage-sensing and, when combined with a GEVI optimized for subthreshold voltages, provides ~100-fold better sensitivity than our prior studies.^7,9^

uSMAART revealed oscillations up to gamma frequencies (~35–100 Hz) in sparse and dense neural populations in behaving mice, including cross-frequency coupling (CFC) in which a slower oscillation modulates a faster rhythm. While CFC has been recorded electrically and associated with various cognitive states and diseases,^27,32,39–43^ uSMAART revealed CFC in the transmembrane potentials of specific neuron types, including delta (0.5–4 Hz) modulations of low- (30–60 Hz) and high-gamma (70–100 Hz) rhythms in neocortical parvalbumin (PV) interneurons of anesthetized mice, and theta (5–9 Hz) modulations of gamma (40–70 Hz) rhythms in hippocampal PV cells of behaving mice. uSMAART using two GEVIs revealed the joint dynamics of excitatory and inhibitory neuron types in visual cortex and hippocampus.

To complement uSMAART, we created a TEMPO mesoscope to image population activity in two neuron types concurrently in head-restrained mice. This instrument can image low gamma (30–60 Hz) activity across a ~50 mm^2^ field of view (FOV) or take high-speed (300 Hz) snapshots of waves over a ~21 mm^2^ area, at spatial resolutions >10 times finer than dense electrocorticography (ECoG) recordings.^44,45^ In anesthetized mice, TEMPO imaging captured neocortical voltage waves with delta-gamma CFC in PV and layer 2/3 (L2/3) pyramidal cells. In awake mice, visual stimulation elicited traveling gamma waves in these cell types and 3–7 Hz waves at stimulus offset, which we examined with dual cell-type TEMPO. Further, TEMPO imaging enabled pathway-specific recordings and revealed that hippocampal beta rhythms are waves that can propagate along two orthogonal directions, whereas hippocampal theta waves propagate bidirectionally along the CA1-CA3 axis.

Overall, our instruments, reporter mouse, and data analytics are enabling for a wide range of studies of high-frequency voltage dynamics in identified neuron classes of behaving animals.

## RESULTS

### Ultra-sensitive fiber photometry

To track high-frequency oscillations in active mammals, we created uSMAART. Challenges preventing prior fiber-optic apparatuses from capturing high-frequency oscillations include (1) GEVI photometry signals are usually minuscule (~50–250 pW); (2) faster rhythms afford fewer emission photons per oscillation cycle; (3) GEVI emissions change by only ~0.1%–1% per mV; (4) hemodynamic or tissue motion artifacts are comparable or greater than fluorescence voltage signals; and (5) fluctuations in illumination, photodetection, and autofluorescence hinder measurement sensitivity.

Prior TEMPO studies^7,9^ could not observe individual high-frequency waves and averaged over many trials to analyze high-frequency activity. A main noise source was fiber-optic mode-hopping of the laser illumination, which arose during animal locomotion from motion of the fiber (Figures S1A–S1E). Mode-hopping constitutes speckle noise from fluctuating interference between coherent illumination in different modes when the fiber flexes.^46^ To prevent speckle, one could use incoherent light-emitting diodes (LEDs). However, compared with LEDs commonly used for photometry, solid-state lasers can be stably modulated at higher frequencies (~50–100 kHz) at which photoreceivers usually have less noise than at lower ones (e.g., <5 kHz) (Figure S1C). Even with modulated LEDs and detectors of near-zero electronic noise, e.g., photon-counting photomultiplier tubes^47^ or scientific-grade CMOS (sCMOS) cameras,^48–50^ the additional illumination noise precludes the low-noise floor achievable with high-frequency-modulated lasers (Figures S1A and S1B).

These considerations yielded a key principle for uSMAART: select high-frequency-modulated lasers for their superior stability but actively break the illumination’s coherence to avoid fiber-optic mode-hopping noise. With this strategy, uSMAART achieves ultralow-noise illumination and surpasses laser-based photometry without active suppression of speckle.^7,49,51^

**Figure 1.**
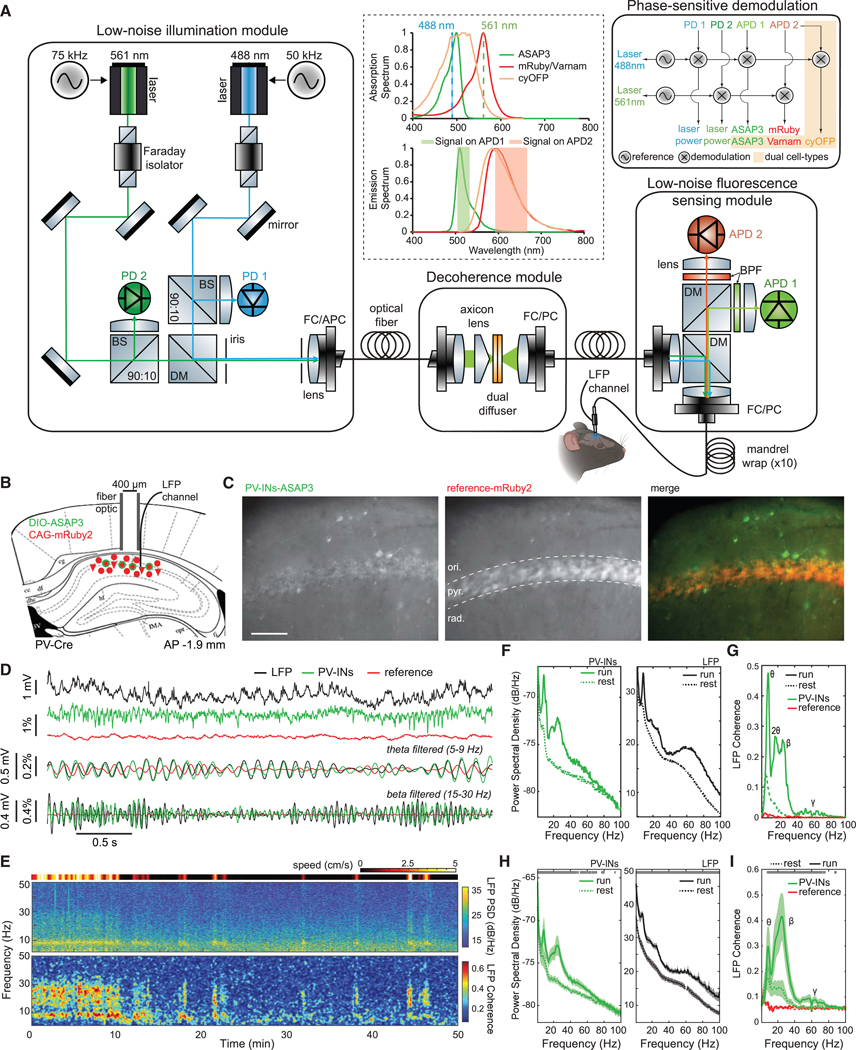
uSMAART photometry captures neural voltage activity up to 100 Hz (A) uSMAART schematic. APD, avalanche photodiode; BPF, bandpass filter; BS, beam splitter; DM, dichroic mirror; FC/APC and FC/PC, angled and flat physical contact fiber connectors; LFP, local field potential; PD, photodiode. Blue and green laser emissions of distinct modulation frequencies are jointly coupled into optical fiber, traverse a dual-stage diffuser, and illuminate the brain via a fiber wrapped around a mandrel. Fluorescence signals from the brain are captured by APDs and undergo phase-sensitive demodulation. Inset: absorption and emission spectra for single- and dual-cell-type voltage sensing, with mRuby and cyOFP, respectively, as reference fluors. See STAR Methods. (B) To monitor hippocampal PV cells (C–I), we virally expressed ASAP3 and mRuby2 in PV-Cre mice and implanted an optical fiber and LFP electrode atop CA1, as shown in a coronal view (−1.9 mm A-P from bregma). (C) Fluorescence images of a coronal section from a PV-Cre mouse showing ASAP3 (left), mRruby2 (middle), and joint (right) expression in strata pyramidale (pyr.), oriens (ori.), and radiatum (rad.) in CA1. Scale bar: 50 μm. (D) LFP (black), ASAP3 (green), and mRuby2 (red) traces in a freely behaving PV-Cre mouse, plus theta- and beta-bandpass-filtered versions. ASAP3 signals are shown reversed in sign relative to all other figures, to highlight anti-correlations to the LFP. (E) Locomotor speed (top), LFP spectrogram (middle), and coherence between LFP and ASAP3 signals (bottom) for a recording with the mouse of (D). (F) Power spectra for LFP (right) and ASAP3 signals (left) for recordings of (E) for times when the mouse was running or resting. Running boosted theta- and beta-band power and coherence (E). (G) Coherence between LFP and either ASAP3 (green) or mRuby2 (red) traces acquired in the mouse of (D) during running or resting. 2θ, a theta harmonic. (H) Same as (F), averaged across 6 mice. Gray dots in (H) and (I): frequencies at which running and resting values differed (signed-rank tests; *p* < 0.05; *n* = 6 mice). Shading: SEM over 6 mice. (I) Same as (G), averaged across *n* = 6 mice. Running increased theta, beta, and gamma activity. Shading: SEM over 6 mice. See also Figures S1 and S2.

uSMAART contains 4 modules for (1) low-noise laser illumination; (2) active decoherence of laser illumination; (3) high-efficiency fluorescence sensing; and (4) digital phase-sensitive detection (Figure 1A). The illumination is ~10 times more stable than in prior voltage-sensing photometry systems,^7,9,52,53^ and, owing to the decoherence module, is impervious to fiber motion (~0.0025% r.m.s. illumination fluctuations across 25–100 Hz) (Figures S1D and S1E). The sensing module minimizes bleed-through into the GEVI channel of emissions and shot noise from the reference fluor. Phase-sensitive detection separates emissions excited by different lasers, removing GEVI signals that bleed into the reference channel.

To evaluate these improvements, we generated artificial 50-Hz signals using a movable fluorescent sample and verified uSMAART boosts sensitivity ~10-fold over prior fiber-optic voltage sensing (Figures S1F–S1I). For *in vivo* studies, we took LFP and uSMAART recordings at the same tissue sites and stipulated that, as in our prior studies of low-frequency rhythms,^7,9^ all high-frequency optical voltage events should accompany a coherent rise in LFP signals at the same frequencies, not just a concomitant rise in high-frequency LFP power^7,9^ (Figures S1J–S1N). While this criterion might exclude optical signals from neural activity that is incoherent with the LFP (see Discussion), it boosts confidence in the electrophysiological origin of small-amplitude, high-frequency optical signals. We applied this coherence criterion to every biological phenomenon studied in this paper but not every recording, as we sometimes omitted LFP measurements for simplicity.

For dual-cell-type recordings, we used green and red GEVIs and a long Stokes-shift fluorescent protein, cyan-excitable orange-red fluorescent protein (cyOFP),^54^ that is excited by blue light, as is the green GEVI, but emits red fluorescence (Figure 1A). Using two light sources modulated at distinct frequencies, uSMAART unambiguously separated signals from 3 fluors using 2 color channels.

**Figure 2.**
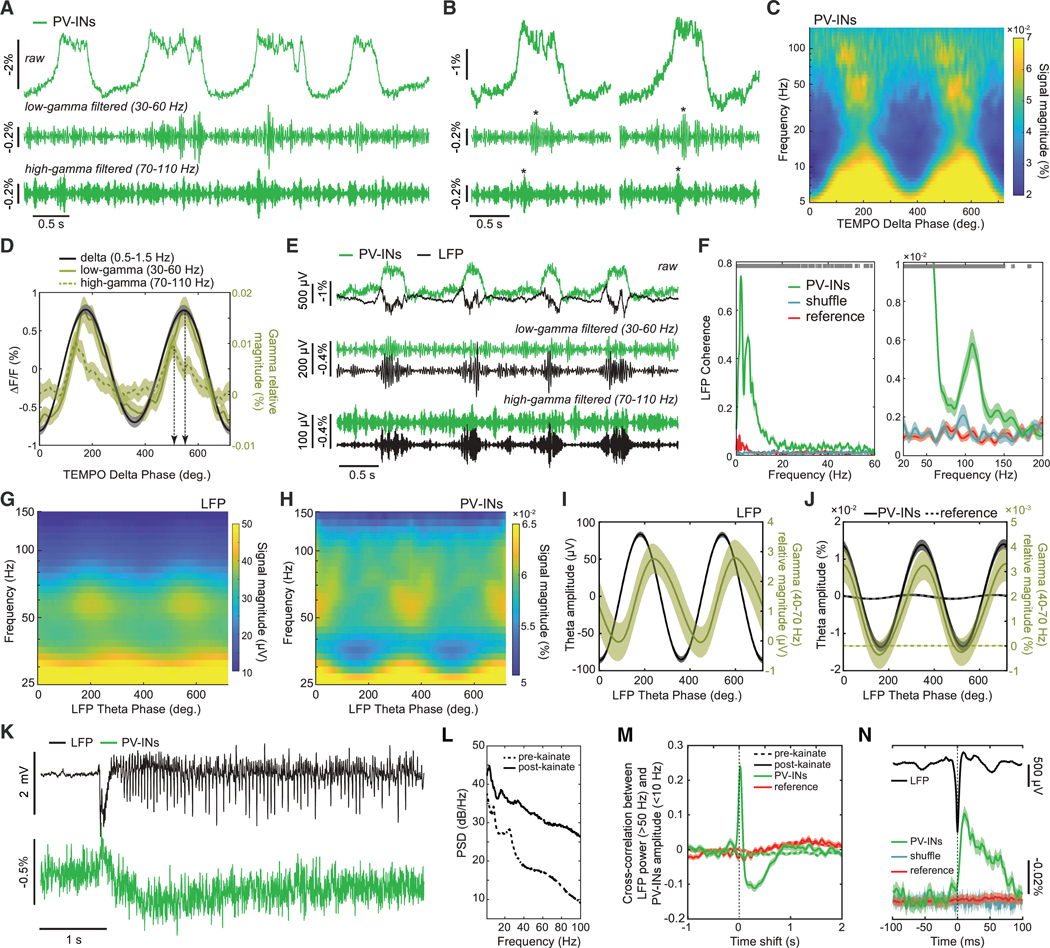
uSMAART captures cell-type-specific CFC (A–F) Delta-gamma coupling in ASAP3-labeled PV cells in area V1 of KX-anesthetized mice. (A and B) PV cell voltage traces (upper) showing delta oscillations. Low- (middle) and high- (bottom) gamma activity rose during up-states in the delta rhythm. Asterisks in (B) mark low- and high-gamma events. (C) Wavelet spectrogram for the mouse of (A) averaged over 257 delta cycles (0.9 Hz), showing two gamma peaks at distinct delta phases. Maximal hyperpolarization of delta is at 0°. (D) Mean voltage signals in the delta band (1.0 ± 0.5 Hz; black, left axis) and delta-phase-dependent modulation (right axis) of low- (solid olive) and high-gamma (dashed olive) activity. Arrows mark phases of peak low- or high-gamma activity. Shading: SEM over 122 events. (E) Raw, low-, and high-gamma-band-filtered traces of LFP and PV cell TEMPO signals in a different mouse than in (A)–(D). (F) Plots of coherence between LFP signals and those of ASAP3-labeled PV cells (green), temporally shuffled ASAP3 traces (teal), or the reference fluor (red) for the mouse of (E) computed across 2-s (left) or 0.2-s (right) intervals. Gray dots: frequencies at which the green and red curves differ significantly (rank-sum test; *p* < 0.05). Shading: SEM over 10 epochs, each of 44 s. (G–J) Theta-gamma coupling in PV cells of dorsal CA1 in an active mouse (labeling strategy of Figure 1B). (G and H) Wavelet spectrograms for LFP (G) and PV cell voltage (H) recordings aligned to LFP theta phase over two theta cycles (7.5 Hz). Maximal hyperpolarization of the LFP is at 0°. (I and J) Mean theta- (black; left axes) and gamma-band (olive; right axes) signal amplitudes for (I) LFP and (J) ASAP3 (solid) or mRuby2 (dashed) fluorescence signals. We made similar findings in *n* = 4 mice. Shading: 95% confidence interval (CI). (K–N) LFP and PV cell voltage signals during kainate-induced seizures in CA1 of an active mouse. (K) LFP- and ASAP3-labeled PV cell voltage traces, showing that an epileptic ictal spike in the former led to a ~100 ms depolarization in the latter. (L) LFP power spectra pre- (dashed) or post- (solid) kainate injection. (M) Cross-correlations between high-frequency (>50 Hz) LFP power and the amplitude (<10 Hz) of PV cell voltage (green) or reference fluor (red) signals pre- (dashed) or post- (solid) kainate injection. Shading: 95% CI over 21 epochs of 5 s, covering either pre-injection or seizure periods. (N) Ictal spike-triggered average activity in LFP, PV cell, reference, and temporally shuffled PV cell traces, showing PV cell depolarization after ictal spike onset. Shading: 95% CI over 21 epochs of 5 s, covering either pre-injection or seizure periods.

**Figure 3.**
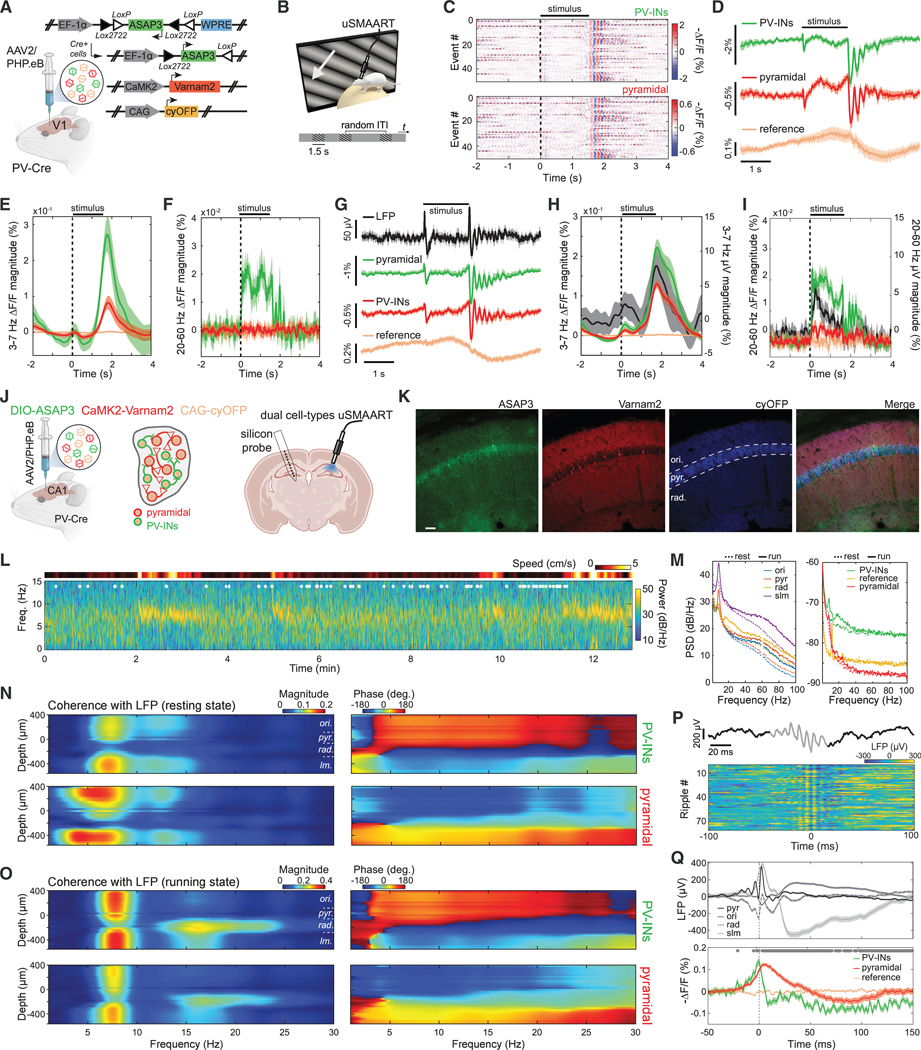
uSMAART tracks voltage activity in two neuron types concurrently in active mice (A–I) Visual stimulation evoked gamma oscillations in area V1 of awake mice. 3–7 Hz oscillations arose after stimulus offset. (A) Retro-orbital injection of 3 AAVs (PHP.eB serotype) in PV-Cre mice allowed expression of ASAP3 in PV cells, Varnam2 in pyramidal cells, and cyOFP in all neuron types. (B) Head-fixed mice viewed a monitor with one eye as we recorded voltage activity in the contralateral V1. Gratings drifted across the monitor (1.5-s trials spaced 2–5 s apart). (C) Visual stimuli evoked post-stimulus 3–7 Hz rhythms in PV and pyramidal cells. Each row shows one of 50 trials in the same mouse. (D) Mean fluorescence traces for all 3 fluors averaged over all 50 trials in (C). Shading in (D)–(I) shows 95% CI across trials. (E and F) Trial-averaged signal magnitudes (3–7 Hz in E; gamma [30–70 Hz] in F) computed for all 3 fluors via wavelet transforms. (G) Trial-averaged traces (*n* = 100 trials) for studies with opposite GEVI assignments to those in (A). (H and I) Same as (E) and (F) but for the studies of (G). (J) To study hippocampal ripple (120–200 Hz) events (J–Q), we performed dual-cell-type uSMAART (same viruses as A) and electrophysiological (32-channel silicon probe) recordings in area CA1 of freely moving PV-Cre mice. (K) Confocal images showing expression of ASAP3 (PV cells), Varnam2 (pyramidal cells), cyOFP (pan-neuronal), and an overlay. Scale bar: 100 μm. (L) Top: mouse speed. Bottom: spectrogram of LFP signals in CA1 stratum pyramidale. White asterisks: ripple events. Theta and ripple activity arose during locomotion and rest, respectively. (M) Power spectra of (left) LFP signals from CA1 (ori., stratum oriens; pyr, stratum pyramidale; rad., stratum radiatum; slm, stratum lacunosum moleculare) and (right) all 3 TEMPO channels during resting or running. (N and O) Coherence magnitude (left) and phase (right) between PV (top plots) or pyramidal cell (bottom plots) voltage and LFP signals across CA1 layers (0 mm denotes stratum pyramidale) during rest (N) or running (O). Theta and beta activity were cell-type, laminar, and behavioral-state-dependent. (P) Top: example ripple event (light gray) in a stratum pyramidale LFP recording. Bottom: LFP traces for 80 ripple events in the same mouse, aligned to ripple trough. (Q) PV and pyramidal cell voltage (bottom) and layer-dependent LFP (top) signals averaged over 273 ripples. Dashed lines: ripple peak. Gray dots mark times with significant differences between cell types (signed-rank test; *p* < 0.05). Shading: SEM. See also Figure S3.

We explored uSMAART’s capabilities, first with a densely labeled neuron class in head-fixed mice (Figures S2A–S2L), then a sparse cell class in freely moving mice (Figures 1 and 2), and then studies of two cell types (Figures 3, S2M–S2Q, and S3). We used various GEVIs, including the fluorescence resonance energy transfer (FRET)-opsins Ace-mNeon1,^2^ Varnam1,^8^ and Varnam2,^1^ and the green fluorescent protein (GFP)- based ASAP3.^55^ For detecting subthreshold oscillations, ASAP3 proved superior due to its much greater subthreshold sensitivity (Figure S1O). Combining uSMAART and ASAP3—each providing a ~10-fold sensitivity gain (Figures S1I and S1O, respectively)—improved sensitivity ~100-fold over prior TEMPO measurements,^7,9^ revealing high-frequency activity in single trials (next sections).

### High-frequency voltage dynamics in freely behaving animals

We first examined whether uSMAART could sense high-frequency activity in primary visual cortex (V1) of awake head-fixed mice that viewed drifting grating stimuli and expressed Varnam1 in pyramidal cells and GFP non-selectively (Figures S2A and S2B). Consistent with past work,^56–58^ visually evoked 3–7 Hz oscillations arose in TEMPO and LFP signals (Figures S2C–S2E). During visual stimulation, the unmixed GEVI but not the GFP channel increased its coherence with the LFP over frequencies ≤30 Hz (Figure S2F), verifying TEMPO captured cell-type-specific high-frequency dynamics.

Next, we recorded high-frequency activity in a sparse cell type in head-fixed active mice (Figures S2G–S2L). In hippocampal area CA1, we expressed ASAP3 in PV interneurons and the reference fluor mRuby2 non-selectively (Figure S2G). Delivery of an air puff to mice at rest triggered locomotor-evoked ASAP3 and LFP signals in the beta range (15–30 Hz), while reference signals reflected slow (~1 Hz) hemodynamic and heartbeat (~10–12 Hz) artifacts (Figures S2H–S2K). Locomotor-triggered rises in coherence between LFP and ASAP3 signals supported the observations of PV cells’ beta-frequency dynamics (Figure S2L).

We next studied CA1 PV cells in mice exploring an arena. Locomotion altered both LFP and PV cell TEMPO signals, as during running, TEMPO voltage but not reference signals increased their power and coherence with the LFP at theta (5–9 Hz), beta (15–30 Hz), and gamma (30–100 Hz) frequencies (*p* < 0.05; signed-rank tests; *n* = 6 mice) (Figures 1A–1I).

### CFC between low- and high-frequency oscillations

Given our ability to capture high-frequency dynamics, we explored whether CFC, associated with various human cognitive processes, could be observed in individual neuron types. We were initially unsure whether this would be feasible, as CFC might be a collective phenomenon involving non-stationary contributions of diverse cell types.^59–62^

We first studied cortical delta-gamma coupling in anesthetized mice (Figures 2A–2F), previously characterized with electrical recordings.^63^ TEMPO signals in visual cortical PV cells expressing ASAP3 revealed prominent delta rhythms^7^ plus gamma frequency bursts during delta up-states^63^ (Figures 2A and 2B). Strikingly, gamma bursts arose in two distinct bands, 30–60 Hz and 70–110 Hz, at distinct phases of delta (Figures 2C and 2D). The apex of high-gamma activity preceded the peak delta depolarization (−56 ± 14 ms; mean ± SEM; *p* < 10^−4^; *n* = 122 delta oscillations; signed-rank test), whereas the low-gamma apex followed it (17 ± 6 ms; *p* < 0.005). Both gamma oscillations cohered with LFP signals significantly more than the reference channel did (Figure 2E and 2F).

We next examined freely behaving mice expressing ASAP3 in hippocampal PV cells, which displayed theta-gamma CFC. The amplitude of PV cell gamma activity peaked at the apex of the LFP theta oscillation, whereas the peak amplitude of LFP gamma activity was phase-shifted relative to the LFP theta oscillation (Figures 2G–2J), supporting reports PV cells exert a hyperpolarizing influence at the theta rhythm apex.^64^

### Bi-phasic dynamics of PV interneurons during epileptiform activity

Dysfunctional PV cells are implicated in several disorders, including epilepsy,^65,66^ in which imbalanced excitatory and inhibitory activity induces hyperexcitability and seizures.^67^ Notably, abnormal PV cell dynamics can impact the regulation of excitatory pyramidal cells. To examine these issues, we induced seizures with kainic acid in mice expressing ASAP3 in PV cells and mRuby2 non-selectively in hippocampal area CA1. During unrestrained mouse behavior, we recorded LFP and TEMPO signals (Figures 2K and 2L).

During epileptiform events, PV cells underwent two successive changes. First, they depolarized for ~100 ms, during which LFPs revealed extracellular hyperpolarization (i.e., intracellular depolarization inducing a local extracellular current sink) (Figures 2M and 2N). Then, PV cells hyperpolarized for ~1 s as CA1 became hyperexcitable and exhibited ictal spikes, high-frequency bursts likely reflecting positive feedback between excitatory and inhibitory cells.^68^ During this second phase of seizure activity, another interneuron-type might suppress PV cell discharge. The PV cell ictal spikes observed here fit with prior recordings,^69^ support evidence for dynamical shifts between excitation and inhibition during epileptiform events,^70^ and illustrate TEMPO can help reveal how different neuron types influence epilepsy generation.

### Dual-neuron-class recordings of visually evoked activity in awake mice

To assess whether uSMAART can report the concurrent dynamics of glutamatergic and GABAergic cell populations, we studied head-fixed mice expressing cyOFP non-selectively as well as Ace-mNeon1 and Varnam1 in pyramidal cells and interneurons, respectively, during passive viewing of visual stimuli (Figures S2M–S2P). Similar to prior results (Figures S2A–S2F), these older GEVIs reported stimulus-evoked 3–7 Hz but not gamma oscillations in both cell types (Figure S2Q).

To capture excitatory/inhibitory interactions in more detail, we prepared mice expressing ASAP3 in PV cells and Varnam2 in pyramidal cells (Figure 3A). 3–7 Hz oscillations consistently arose in both cell types at stimulus offset (Figures 3B–3E). ASAP3’s superior dynamic range also enabled detection of stimulus-evoked gamma (30–70 Hz) activity in PV cells (Figure 3F). To verify that the lack of observed gamma activation in pyramidal cells reflected indicator assignments, rather than true differences between cell types, we prepared mice with reversed indicator assignments and performed joint LFP and TEMPO recordings (Figures 3G–3I). In these mice, both cell types and the LFP exhibited post-stimulus 3–7 Hz activation (Figure 3H), and pyramidal cells showed the expected increases in gamma activity during visual stimulation, as did LFP signals (Figure 3I).

### Dual-neuron-class recordings in freely moving mice

We next studied hippocampal activity via dual-cell-type uSMAART recordings in freely behaving mice expressing cyOFP non-selectively, ASAP3 in PV cells, and Varnam2 in pyramidal cells (Figures 3J–3Q and S3A–S3F). To identify neurophysiological signatures of different laminae, we performed linearprobe electrical recordings in the contralateral hippocampus (Figures S3A and S3B).^71–73^ As in prior studies, LFP power in theta (5–9 Hz) and gamma (30–100 Hz) frequencies rose across laminae during locomotion, but sharp-wave ripples (120–200 Hz) occurred mainly during rest (Figures 3L and 3M). Locomotor-evoked increases in LFP beta (15–30 Hz) activity were more limited to stratum radiatum. In both neuron types but especially ASAP3-labeled PV cells, locomotion triggered prominent theta and beta signals, supporting prior results (Figures 1 and 3M).

During rest, PV and pyramidal cell dynamics were coherent with the LFP but with distinct frequency signatures and phase relationships, both varying across laminae (Figures 3N and S3C–S3E). Oscillations in the two cell types exhibited roughly opposite phase relationships with the LFP and phase inversions near stratum radiatum. During running, both cell types had theta (5–9 Hz) activity of increased coherence with the LFP in nearly all CA1 layers and beta (15–30 Hz) and gamma (30–70 Hz) dynamics more selectively coherent with the LFP in stratum radiatum (Figures 3O and S3C–S3E).

During ripples (Figure 3P), PV and pyramidal cells depolarized at ripple onset, consistent with reports pyramidal cell activity peaks at the ripple envelope’s apex.^74,75^ Next, there was a sharp PV cell hyperpolarization preceding the depolarization peak of pyramidal cells, and then a more gradual pyramidal cell hyperpolarization (Figures 3Q and S3F). The rapid PV cell hyperpolarization is suggestive of a time-gating mechanism for precisely releasing synchronized pyramidal cell firing, consistent with the idea that inhibitory control by PV interneurons shapes pyramidal cell activity during sharp-wave ripples.^72,76^ Future studies should explore how such interactions affect memory encoding and consolidation.

### Concurrent recordings in two brain areas

**Figure 4.**
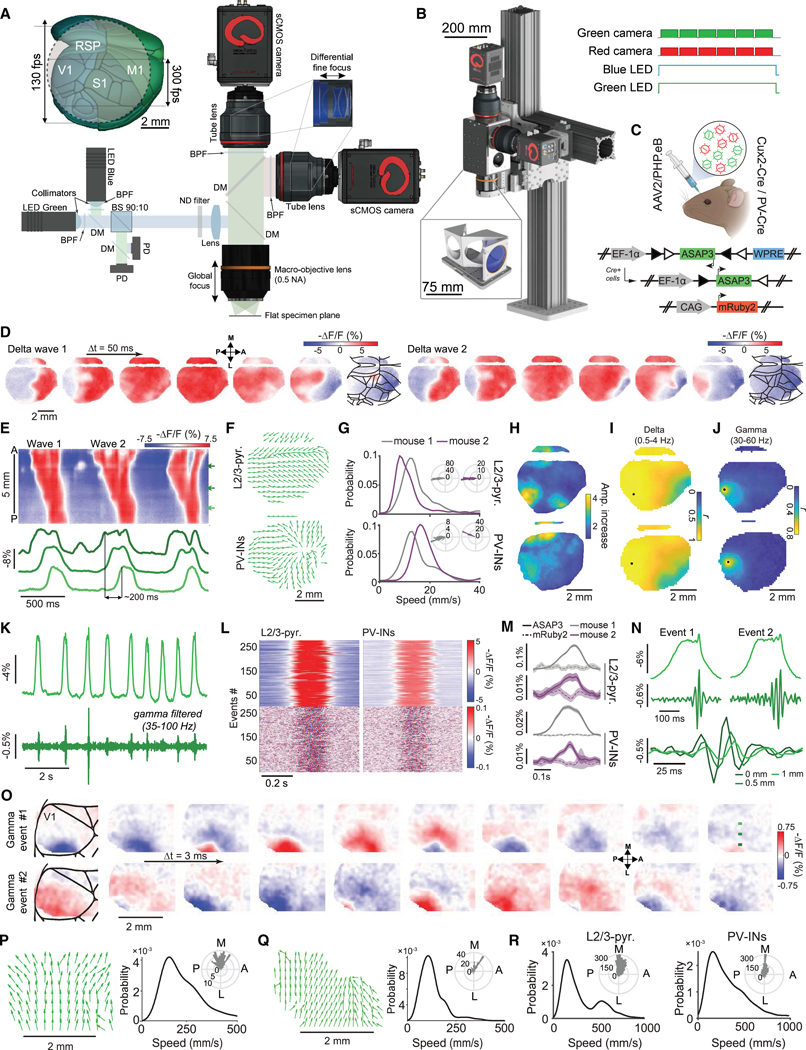
Traveling cortical voltage waves with delta-gamma coupling in anesthetized mice (A and B) Optical (A) and mechanical (B) designs of the TEMPO mesoscope. Light from two low-noise LEDs is monitored by photodiodes, reflects off a dual-band dichroic mirror (70 × 100 mm^2^) and reaches the specimen via a 0.5 NA objective lens. sCMOS cameras allow dual-color fluorescence detection. Insets: (A) cranial window, aligned to Allen Brain Atlas, used in this figure and Figures 5 and 7 to image across a 7–8 mm diameter (130 fps) or a sub-region (300 fps). (B) Lower left, large custom filter set. Upper right, imaging protocol for a green GEVI and a red reference fluor used continuous illumination and externally triggered image acquisition (800 Mbytes·s^−1^ per camera). BPF, bandpass filter; BS, beamsplitter; DM, dichroic mirror; LED, light-emitting diode; M1, primary motor cortex; ND, neutral density; PD, photodiode; RSP, retrosplenial cortex; S1, primary somatosensory cortex; V1, primary visual cortex. (C) Retro-orbitally injected AAV2/PHP.eB viruses expressed mRuby2 and Cre-dependent ASAP3 via CAG and EF-1α promoters, respectively, in either Cux2-Cre^ERT2^ or PV-Cre mice. Imaging in KX-anesthetized mice was at 130 fps (D–J) or 300 fps (K–R). See Video S2. (D) Images (50 ms apart) of cortical L2/3 pyramidal cells in a Cux2-Cre^ERT2^ mouse. Delta wave depolarization (red hues) swept anterior to posterior. Images were unmixed (Figure S5) but otherwise unfiltered. Brain area boundaries (A, inset) are in rightmost images. (E) Space-time plot (top) shows the anterior to posterior travel of waves in (D). Colors: mean signals averaged across the medio-lateral axis. Arrows: Anterior-posterior (A-P) locations for 3 color-corresponding fluorescence voltage traces (bottom). (F) Flow maps for example delta waves in Cux2-Cre^ERT2^ (top) and PV-Cre (bottom) mice. Here and in all figures, flow vectors are shown with uniform length. (G) Delta wave speed distributions in area V1 of 2 Cux2-Cre^ERT2^ (top) and 2 PV-Cre (bottom) mice. Insets: polar histograms of wave direction (200–405 delta events per mouse). (H–J) Maps characterizing coupled delta (0.5–4 Hz) and gamma (30–60 Hz) waves in example Cux2-Cre^ERT2^ (top) and PV-Cre (bottom) mice. (H) Gamma activity amplitudes rose during delta wave maxima up to ∼4-fold over baseline values in V1 and RSP. (I and J) Correlation coefficients, *r*, denoting the peak value of the temporal correlation function between each point’s voltage (I) or gamma-band-filtered (J) voltage trace and that at the black dot in V1. (K) Fluorescence voltage (top) and gamma-band-filtered (35–100 Hz, bottom) traces from V1 of the Cux2-Cre^ERT2^ mouse of (H)–(J). (L) Top: voltage (top) and gamma-band-filtered (35–100 Hz, bottom) traces from V1 of the 2 mice in (H)–(J) aligned to delta peaks, showing delta-gamma coupling (260 delta events per mouse). (M) Mean amplitudes of gamma-band voltage (solid) and reference (dashed) traces aligned to the delta peak and averaged over all delta events in each mouse of (G). Shading: 95% CI. (N and O) Traces of coupled delta (N, top) and gamma-band (N, middle) activity in V1 of the mouse of (K) and corresponding gamma-band-filtered image sequences (O). Gamma-filtered traces (N, bottom) are from the color-corresponding dot locations in the rightmost frame of (O). (Images in O were spatially low-pass-filtered for display purposes only). (P and Q) Flow maps (left) and speed distributions (right) for gamma events #1 (P) and #2 (Q) of (N) and (O). Insets: polar histograms of wave directions across the maps. (R) Wave speed distributions and direction histograms for 20 gamma events and all locations in V1 for mice of (H)–(J). Wave directions are roughly orthogonal to the carrier delta waves (E–G). See also Figures S4, S5, and S6.

Capabilities for tracking multiple neuron types and brain regions concurrently would empower studies of inter-area dynamics. To this end, uSMAART permits joint recordings of two cell types in each of two areas. In ketamine-xylazine (KX)-anesthetized mice expressing ASAP3 in neocortical PV cells and Varnam2 in pyramidal cells, both neuron types displayed prominent delta rhythms in V1 and primary motor cortex (M1) (Figures S3G–S3I). Supporting prior results (Figure 2), PV cells exhibited delta-gamma CFC in both V1 and M1, with M1 delta activity leading that in V1 by 103 ± 41 ms (mean ± SD) (Figures S3J–S3M). The same calculation for pyramidal cells yielded an estimated lead in M1 of 117 ± 35 ms. These results are suggestive of a wave traveling 10–15 mm·s^−1^ from anterior to posterior, prompting us to perform follow-up imaging studies (Figure 4).

### TEMPO mesoscope

As uSMAART records signals near the fiber tip, we created a complementary instrument, the TEMPO mesoscope, to image voltage dynamics across the cortex of head-restrained mice (Figures 4A, 4B, and S4A–S4K). The instrument design embodies the TEMPO approach and has 3 main features: (1) a pair of high-power, low-noise LEDs for exciting two GEVIs plus a reference fluor; (2) a low-aberration, high numerical aperture (NA) macro-objective lens to image a FOV up to 8 mm in diameter; and (3) two detection pathways, each with an sCMOS camera, to track GEVI and reference fluor emissions. During full-frame imaging, the cameras operated at 130 fps, above the Nyquist sampling rates for low-gamma band activity (30–60 Hz). To detect higher frequency activity or capture fine temporal snapshots of wave propagation, we imaged at 300 fps over narrower FOVs (2.7 × 8 mm) (Figure 4A, inset). In either mode, we acquired ~1 TB of raw data in ~10 min, from which we unmixed voltage signals from biological and instrumentation artifacts.

### Frequency-dependent unmixing of voltage and artifact signals

Unmixing artifacts and noise captured in the reference channel from voltage signals is a key facet of TEMPO.^7–9^ Initially, we used independent component analysis for unmixing^7^ and later tried linear regression.^8^ However, these algorithms poorly account for the variable extents that different artifacts impact different frequency bands (Figures S5A and S5B), necessitating better analytics.

Specifically, heartbeat-related artifacts and changes in tissue’s optical properties from blood volume and oxygenation dynamics have distinct temporal frequency signatures and phase shifts between the GEVI and reference channels (Figure S5B). Prior unmixing approaches used frequency-independent or zero-phase-lag transformations that cannot capture frequency-dependent, non-zero-phase lags.^7,8,13,77–79^ We created convolutional filtering methods^80^ to remove artifacts from neural voltage signals in a frequency-dependent way that also accounts for the spatial heterogeneity of hemodynamics^79,81^ (Figures S5C–S5M).

The algorithm first estimates a linear filter describing how frequency-dependent artifacts in the reference channel affect the GEVI channel. Using this filter to estimate and then subtract non-voltage signals from the GEVI channel, the algorithm estimates the true voltage signals. This method outperformed a simple (i.e., frequency-independent) linear regression, as it better removed broad- and narrow-band artifacts and did not transfer noise from the reference to the GEVI channel, crucial for sensitivity to high-frequency activity (Figures S5E–S5J). When applied to individual or aggregated pixels in TEMPO videos, our approach captured the heterogeneity of hemodynamics across different blood vessels (Figures S5K and S5L). Altogether, convolutional unmixing preserves high-frequency voltage signals in photometry and video datasets and is an important facet of all our analyses.

### Transgenic reporter mice

To co-express the Ace-mNeon1^2^ voltage sensor and mRuby3^82^ reference fluor, we created Cre-dependent reporter mice customized for TEMPO, using our published Flp-in approach to make a TIGRE 2.0-based mouse line (Figure S6A).^83–85^ This mouse (termed Ai218) enables convenient, uniform expression of both fluorescent proteins. For evaluations, we crossed Ai218 with PV-Cre or Cux2-Cre^ERT2^ mice to express Ace-mNeon1 and mRuby3 in PV or L2/3 pyramidal cells, respectively (Figures S6B and S6C). To image widely across a cortical hemisphere, we created a mouse preparation with a circular glass window of 7–8 mm diameter installed in the cranium, allowing repeated imaging over months.

In KX-anesthetized Cux2-Cre^ERT2^ × Ai218 mice, TEMPO imaging revealed up-down state transitions traveling anterior to posterior (Figures S6D–S6G). These delta (0.5–4 Hz) waves were spatially coherent across most imaged areas (Figure S6H). However, unlike uSMAART and past electrical^63^ measurements (Figures 2 and S3), we did not see gamma-range (30–100 Hz) dynamics (Figures S6I–S6K), likely due to the 10-fold poorer subthreshold sensitivity of Ace-mNeon1 relative to ASAP3 (Figure S1O).

Next, we imaged awake PV-Cre × Ai218 mice viewing moving grating stimuli (Figure S6L). Strong 3–7 Hz oscillations arose at stimulus offset in V1 but not other brain areas (Figures S6M–S6R). Visually evoked gamma activity (20–50 Hz) arose in visual cortex (Figures S6O, S6Q, and S6S), consistent with uSMAART data (Figures 3A–3I, S2A–S2F, and S2M–S2Q). Overall, Ai218 mice enabled explorations of cell-type-specific oscillations up to the low gamma range.

### Traveling waves with delta-gamma CFC

To complement Ai218 mice, we created PHP.eB serotype^86^ adeno-associated viruses (AAVs) for brain-wide expression of newer GEVIs.^1,55^ To image cortical PV or L2/3 pyramidal cells, we retro-orbitally co-injected into PV-Cre or Cux2-Cre^ERT2^ mice, respectively, a pair of AAV2/PHP.eB viruses driving Cre-dependent expression of ASAP3 and non-selective expression of mRuby2 (Figures 4C, S6T, and S6U). TEMPO imaging with these mice under KX-anesthesia revealed delta waves sweeping anterior to posterior across neocortex (Figures 4D–4G; Video S1) (*n* = 200–405 delta events per mouse; speeds: 12.0 ± 0.4 mm·s^−1^ for Cux2-Cre^ERT2^ mice, 15.7 ± 0.9 mm·s^−1^ for PV-Cre mice; angle from anterior axis: 176° ± 1° for Cux2-Cre^ERT2^ mice, 164° ± 1° for PV-Cre mice; mean ± SEM). Localized gamma (30–60 Hz) activity arose during delta wave peaks and, unlike delta activity, was confined to a ∼2-mm-wide area (Figures 4H–4J).

To further characterize gamma activity, we took 300-fps-videos over a smaller FOV that revealed gamma waves nested into delta wave peaks in both L2/3 pyramidal and PV cells (Figures 4K–4N; Video S2). Unlike delta waves, gamma waves traveled lateral to medial (72° ± 3° and 88° ± 5° from the anterior-posterior (A-P) axis in Cux2-Cre^ERT2^ and PV-Cre mice, respectively; mean ± SEM over *n* = 200 and 300 waves, respectively) and were also much faster (225 ± 28 and 279 ± 20 mm·s^−1^ in Cux2-Cre^ERT2^ and PV-Cre mice, respectively) (Figures 4O–4R). This striking observation that coupled waves need not co-propagate in the same direction highlights how TEMPO imaging can uncover the complex spatiotemporal patterns of CFC and traveling wave dynamics.

### Visually evoked sequences of traveling gamma and 3–7 Hz waves in awake mice

**Figure 5.**
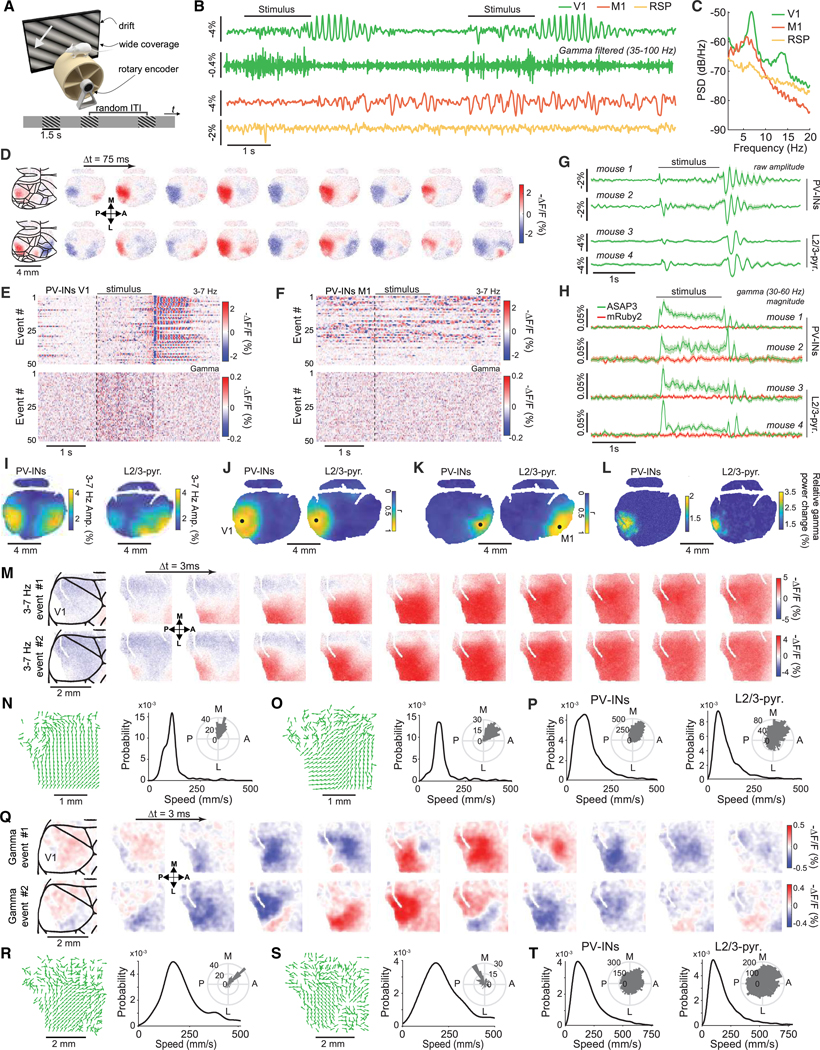
Successive visually evoked gamma and 3–7 Hz waves in V1 of awake mice (A) Visual stimulation was as in Figure 3B. Labeling was as in Figure 4C. (B and C) Traces (B) and power spectra (over 276-s recording) (C) of PV cell voltage signals averaged over visual (V1), motor (M1), or retrosplenial (RSP) cortices. In V1, visual stimuli evoked gamma activity and 3–7 Hz rhythms arose after stimulus offset. (D) Image sequences of visually evoked 3–7 Hz waves in PV cells of V1. Brain area boundaries shown at left. (E and F) Raster plots of raw (top) or gamma (30–60 Hz, bottom) PV cell voltage activity averaged over V1 (E) or M1 (F) showing V1 gamma and 3–7 Hz oscillations evoked during and after visual stimulation, respectively (50 trials; mouse of B–D). (G and H) Mean activity (G) and gamma-band signal magnitudes (H, computed via wavelet spectrogram) for 2 PV-Cre and 2 Cux2-Cre^ERT2^ mice, each with significantly elevated gamma signals during visual stimulation (*p* < 10^−14^ for a 0.5-s interval in the middle of stimulation compared to baseline values; *n* = 50 trials; rank-sum test). Shading: 95% CI over trials. (I–L) Maps of 3–7 Hz power (I) correlation coefficients (J and K) computed as in Figure 4I between each point and that at the black dot in V1 (J) or M1 (K), and visually evoked rises in gamma-band (30–60 Hz) signal amplitudes (L) for mice 1 and 3 in (H) (recordings of 275 and 266 s, respectively). Brain area boundaries are shown in (D). (M) Images (300 fps) showing two 3–7 Hz waves in V1 from mouse 1. (N and O) Flow maps (left) and speed distributions and direction histograms (right) for local wave propagation for events #1 (N) and #2 (O) of (M). (P) Speed distributions and direction histograms across all locations in V1 and 30 wave events (3–7 Hz) for each mouse of (I)–(L). (Q) Images (300 fps) of gamma-filtered (35–100 Hz) activity showing two gamma events in V1 from mouse of (M). Images were spatially low-pass filtered for display purposes only. (R and S) Flow maps (left) and distributions of speed and histograms of direction (right) for local wave propagation for events #1 (R) and #2 (S) of (Q). (T) Speed distributions and direction histograms over all locations in V1 and 50 gamma events in each of the PV-Cre and Cux2-Cre^ERT2^ mice of (L). See also Figures S6 and S7A.

We next studied visual processing in awake mice expressing ASAP3 in either cortical PV or L2/3 pyramidal cells, using drifting grating visual stimuli (Figures 5, S6V–S6Y, and S7A). TEMPO imaging revealed gamma (30–60 Hz) waves in visual cortex during stimulus presentation and ~3–7 Hz waves at stimulus offset^56,58^ (Figures 5A–5L; Video S3). Gamma-band power rose significantly in V1 during visual stimulation (*p* < 10^−9^ for each of 2 PV-Cre mice, *p* < 10^−3^ for each of 3 Cux2-Cre^ERT2^ mice, 50 trials each, rank-sum test) (Figures 5H and 5L), unlike reference fluor signals (*p* > 0.27, all mice). Visually evoked 3–7 Hz rhythms, observed previously in electrophysiological studies,^56–58^ also appeared outside visual cortex, such as in M1, but were incoherent with those in V1 (Figures 5I–5K).

We next examined somatostatin (SST) interneurons and layer 4 (L4) and layer 5 (L5) pyramidal cells (Figure S7A). Stimulus-evoked gamma activity in L4 pyramids was far weaker than in other cell types and largely confined to stimulus onset and offset. 3–7 Hz oscillations arose at stimulus offset across cell types, with varying time courses. These characterizations of sensory-evoked oscillations in five V1 neuron types could inform models of visual processing.

We next characterized waves’ spatiotemporal dynamics. In V1, 3–7 Hz waves had similar speeds (108 ± 16 mm·s^−1^ for PV, 138 ± 16 mm·s^−1^ for L2/3 cells) and directions (66° ± 8° relative to the A-P axis for PV, 63° ± 10° for L2/3 cells) in both cell types (*n* = 30 waves each; mean ± SEM) (Figures 5M–5P). Individual gamma waves generally traveled unidirectionally, but the distributions of directions across all gamma waves were approximately isotropic (Figures 5Q–5T). Gamma waves in PV and L2/3 pyramidal cells were faster (178 ± 85 and 145 ± 79 mm·s^−1^, respectively; mean ± SEM; 50 gamma events per cell type) than 3–7 Hz waves (Figures 5P and 5T). However, a slower subset of gamma activity was locked to a 3–7 Hz wave sharing the same speed and direction, suggesting that, unlike the faster gamma waves arising in isolation and with isotopically distributed directions, this slower gamma activity constituted harmonics of 3–7 Hz activity (Figures S6X and S6Y).

### Locomotion-evoked hippocampal theta and beta waves

We next imaged hippocampal activity during rest-to-run transitions. Prior electrophysiological studies of locomotion-associated hippocampal theta waves used electrode array or multi-site recordings with relatively poor spatial resolution, the interpretative limitations of field potential recordings, and a lack of cell-type specificity.^71,87^

**Figure 6.**
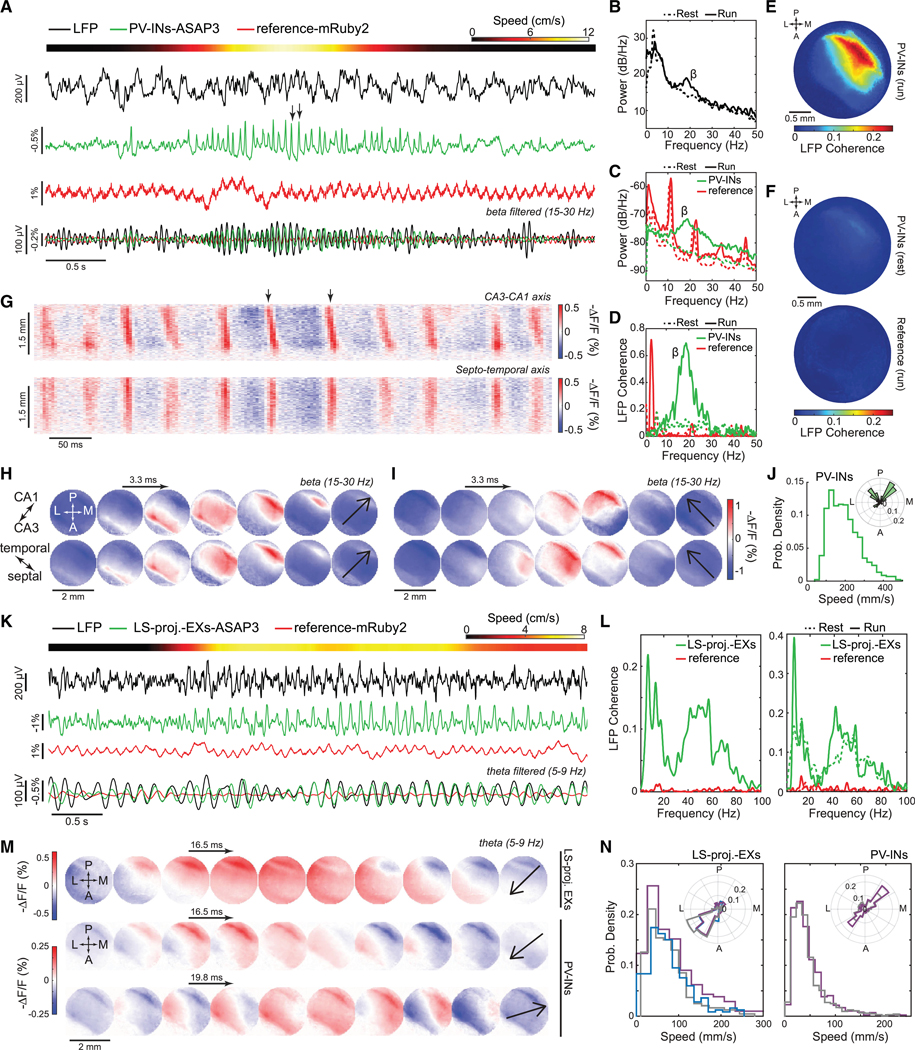
Bidirectional locomotor-evoked theta and beta waves in hippocampus (A) Top: mouse speed. Bottom: example broadband (top 3) and beta-filtered (15–30 Hz, bottom 3) concurrent traces of hippocampal LFP, ASAP3, and mRuby2 signals from a PV-Cre mouse. Fluorescence signals are averaged over the FOV. Arrows: events characterized in (G) and (H). (B–D) Power spectral densities of LFP (B) and fluorescence (C) signals and coherences (D) between them during resting and running for a 5-min recording in the mouse of (A) (see also Figures S7B and S7C). (E and F) Maps of beta-band (15–25 Hz) coherence between LFP and PV cell signals during running (E) or rest (F, top) or with the reference fluor during running (F, bottom). LFP electrode was in CA1 (upper right of the maps). (G) Space-time plots of PV activity during running projected onto CA3-CA1 (top) or septo-temporal (bottom) axes. Arrows: events marked in (A). (H and I) Images (300 fps) showing pairs of beta waves traveling along CA3-to-CA1 (H) or septo-to-temporal (I) axes. Arrows: propagation directions. (J) Distributions of speed and direction across 756 beta waves in 2 mice. (K) Same format as (A) but showing locomotor-evoked theta-band (5–9 Hz) activity in LS-projecting pyramidal cells. (L) Plots of coherence between LFP and either mRuby2 or ASAP signals from LS-projecting pyramidal cells over the entire recording (left) or separately for resting and running epochs (right). (M) Movie frames showing theta waves for LS-projecting pyramidal (top) or PV (middle, bottom) cells. Black arrows: propagation directions. (N) Distributions of speed and direction for theta waves in LS-projecting pyramidal (left; 3 mice) or PV (right; 2 mice) cells (441–1,094 theta waves per mouse).

To overcome these limitations, we imaged ASAP3-expressing PV cells across a ∼2-mm-wide area of dorsal CA1. We took concurrent LFP recordings and used airpuffs to elicit running (Figures 6A–6J). During locomotion, ASAP3 and LFP signals exhibited substantial increases in beta-band power plus increased coherence between these two signals across most of the optical window (Figures 6B–6F, S7B, and S7C). mRuby2 signals showed neither of these. Strikingly, we discovered that hippocampal beta oscillations are traveling waves with two orthogonal propagation modes in the CA3-to-CA1 and septal-to-temporal directions (speeds: 220 ± 4 vs. 220 ± 6 mm·s^−1^; median ± SEM; *n* = 428 and 199 waves traveling CA3-to-CA1 and septal to temporal, respectively, *p* = 0.6; rank-sum test) (Figures 6G–6J).

To image oscillations in a specific pathway, we virally expressed ASAP3 in CA1 pyramidal cells with axons in lateral septum (LS) (Figure 6K). LS-projecting pyramidal cells exhibited broadband coherence with the LFP that rose substantially in the theta (5–9 Hz) band during locomotion (Figure 6L). Beta-band coherence for LS-projecting pyramids was weaker than for PV cells and unchanged during locomotion.

We compared theta wave dynamics in mice with either PV interneurons or LS-projecting pyramids expressing ASAP3 (Figures 6M and 6N). Both neuron types exhibited CA1-to-CA3 wave propagation, but only PV cells exhibited waves traveling in the reverse CA3-to-CA1 direction. Interestingly, theta waves had different speeds in PV (36 ± 1 mm·s^−1^, median ± SEM; 2,024 theta waves; *n* = 2 mice) and LS-projecting (87 ± 2 mm·s^−1^; 1,414 waves; *n* = 3 mice) neurons. Unlike beta waves, for which speeds were statistically indistinguishable for the two propagation directions, the two classes of theta waves in PV cells had different speeds (35 ± 2 and 42 ± 2 mm·s^−1^, median ± SEM, for *n* = 777 CA3-to-CA1 and 637 CA1-to-CA3 waves, respectively; *p* = 0.003; rank-sum test).

Altogether, the discovery that beta oscillations are traveling waves with two orthogonal propagation modes and the observations of forward and reverse traveling directions for theta waves illustrate how TEMPO imaging can reveal the spatiotemporal patterns of voltage activity in specific neuron types or projection pathways.

### Dual-cell-type imaging in visual cortex

**Figure 7.**
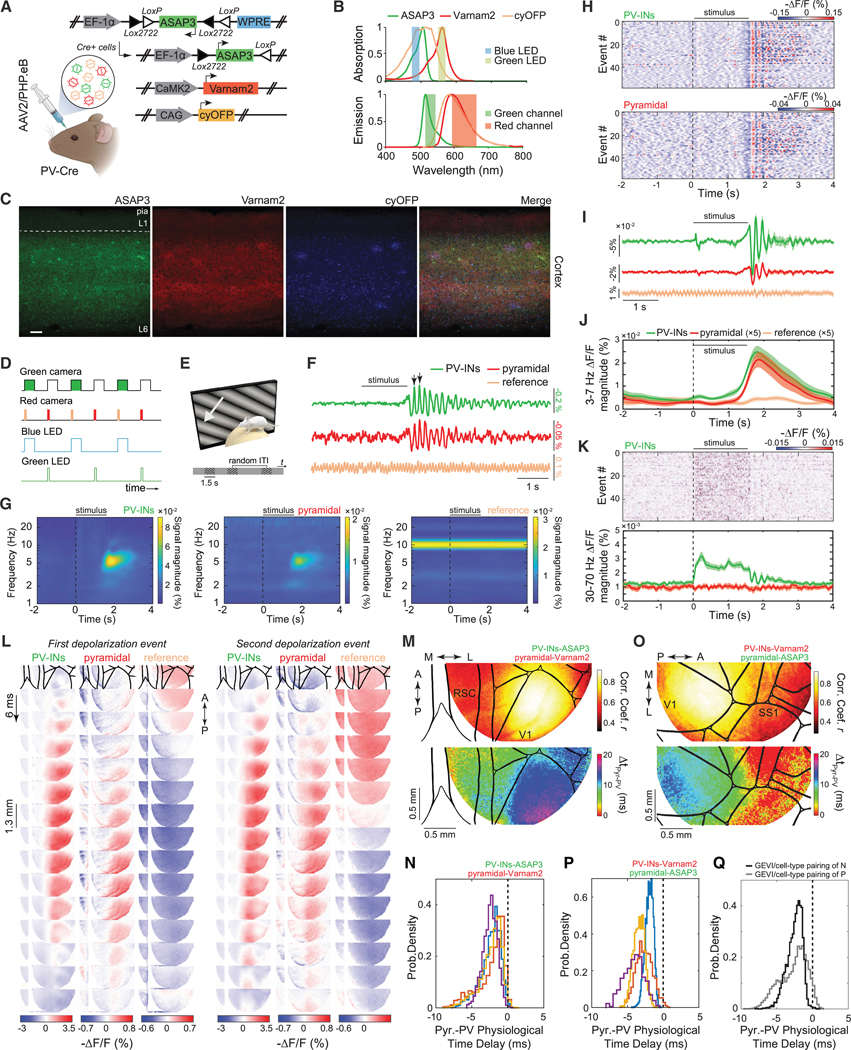
Imaging the concurrent voltage dynamics of two neuron types in behaving mice (A) Dual-cell-type labeling strategy as Figure 3A, except viruses were injected retro-orbitally. (B) Absorption and emission fluorescence spectra with LED spectra and emission filter passbands. (C) Confocal images of ASAP3, Varnam2, cyOFP, and joint expression patterns. L1, layer 1; L6, layer 6. Scale bar: 100 μm. (D) Timing protocol for dual-cell-type TEMPO imaging with 3 fluors and 2 cameras. (E) Visual stimulation paradigm as in Figure 3B. (F) Traces from ASAP3-expressing PV cells and Varnam2-expressing pyramidal cells averaged over V1. 3–7 Hz rhythms arose at stimulation offset. Arrows: events characterized in (L). (G–I) Mean wavelet spectrograms (G), fluorescence voltage traces for PV and pyramidal cells (H), and mean traces (I) averaged across V1 (*n* = 60 visual stimulation trials) for the mouse of (F). Shading in (I)–(K) shows 95% CI over trials. (J) Mean signal magnitudes in the 3–7 Hz band computed via wavelet spectrogram. (K) Top: gamma-band (30–70 Hz) PV cell activity for the 60 trials of (H). Bottom: traces of mean gamma-band magnitudes for all 3 fluors. (L) Images showing the 2 events marked in (F). Brain area boundaries shown at top (see Figure 4A). (M and N) Example maps (M) of correlation coefficients (top) and measured time delays (bottom) between excitatory and inhibitory activity in the 3–7 Hz band averaged over *n* = 98 stimulation trials for the mouse of (L). Distributions (N) of physiological time delays estimated for 4 recordings from 3 mice after correcting for time-lags induced by GEVI kinetics (Figures S7D–S7F). (O and P) Same as (M) and (N), respectively, but for mice with reversed labeling in which pyramidal and PV cells respectively express ASAP3 and Varnam2 (4 recordings total, 4 mice). (Q) Probability distributions aggregating data from (N) (black) and (P) (gray). See also Figures S7D–S7F.

To study the joint activation of neocortical PV and pyramidal cells in awake mice, we performed dual-cell-type TEMPO imaging as mice viewed drifting grating stimuli (Figures 7A–7F). In V1, gratings evoked 3–7 Hz oscillations in both cell types after stimulus offset (Figures 7G–7J). As before (Figures 3A–3I, 5, S2A–S2F, and S7A), we observed stimulus-evoked gamma band activity increases in ASAP3-expressing cells but not in those expressing Varnam2 (Figure 7K).

Since 3–7 Hz waves appeared in both cell types (Figure 7L), we computed maps of the correlation coefficients and temporal delays between the waves in each cell class (Figures 7M and 7O). As expected, correlation coefficients were highest in visual cortex. However, the measured temporal delays differed when we reversed the GEVI assignments to the two neuron types (Figures 7M and 7O). Hence, one or both GEVIs likely induced an apparent temporal delay due to insufficiently fast fluorescence voltage-signaling to report the wave’s instantaneous phase.

Motivated by this inference, we characterized the delays induced by three GEVIs used in this paper, which were non-zero for Varnam2 and Ace-mNeon2 but near zero for ASAP3 (Figures S7D–S7F). Having determined these artifactual delays, we estimated the true physiological delays between visual cortical PV and pyramidal cells during 3–7 Hz waves (Figures 7N–7Q). Reassuringly, these estimated values were statistically indistinguishable regardless of the cell-type assignments of ASAP3 and Varnam2 (2.27 ± 0.02 vs. 2.32 ± 0.01 ms, respectively, for *n* = 332 events from 3 mice in Figure 7N and 230 events from 4 mice in Figure 7P; median ± SEM; *p* = 0.08; rank-sum test) and show that pyramidal cell depolarization preceded that of the PV cells. This agrees with patch-clamp recordings of visually evoked 3–7 Hz oscillations in awake mice^56^ and constrains candidate mechanisms for wave propagation.

## DISCUSSION

TEMPO detects high-frequency dynamics without trial-averaging, and unlike Ca^2+^ imaging, it reports depolarizations and hyperpolarizations about equally well. Unlike voltage-imaging studies of individual neural spikes, which generally show rapid photobleaching,^1,4–6^ TEMPO uses far weaker illumination (∼2 mW/mm^2^), allowing hour-long studies with minimal bleaching. The Ai218 mouse line provides highly reproducible Ace-mNeon1 and mRuby3 expression but makes detection of fast oscillations challenging. With PHP.eB AAVs, we expressed across cortex a more sensitive indicator, ASAP3, plus Varnam2 for dual-cell-type studies. Using these viruses, TEMPO imaging captured individual delta, theta, beta, and gamma waves, illustrating that averaging over waves with distinct directions can mask how individual waves differ^88,89^ (Figures 5R–5T). Further TEMPO studies of waves in targeted neuron types will help reveal their mechanisms and functional roles.

### Reference channel

Early photometry studies commonly used a reference channel to monitor signal artifacts^90^; recent studies often^51,52,91^ but not always^53^ omitted a reference channel. Similarly, some brain imaging studies in head-fixed rodents used an isosbestic or isofluorescent point or multiple excitation wavelengths to track hemodynamic artifacts.^79,92,93^ Omitting a reference channel is problematic when artifacts and signals of interest have similar magnitudes. As most GEVIs lack a convenient voltage-independent wavelength, TEMPO uses a spectrally separable, non-functional fluorophore as a reference.^7,9,78^ For dual-cell-type TEMPO, we used a long Stokes-shift fluorophore. As previously,^7,8,93^ reference fluor emissions captured brain motion, heartbeat, and slow hemodynamic artifacts (Figures 3D, 6A, S5B, and S5E). To unmix these artifacts in a frequency-dependent manner, we used convolutional filtering to capture the variable impact of different noise sources on the GEVI channel. This algorithm is both integral to TEMPO and broadly applicable to other fluorescence measurements.

### Comparisons with prior voltage-mapping techniques

In comparison with prior photometry methods, uSMAART allows fiber-optic measurements of high-frequency oscillations at a single-trial level. The key engineering insight was that, to capitalize on the stability of high-frequency-modulated lasers, one should break the illumination coherence to prevent fiber-optic speckle noise during animal movement. The ∼10-fold improvement over earlier TEMPO apparatus, combined with ASAP3’s superior tracking of subthreshold dynamics, yielded an overall ∼100-fold sensitivity improvement over prior fiber-optic voltage sensing (Figures S1I and S1O).^7,9^

To map field potentials across the brain surface, prior work used voltage-sensitive dyes (VSDs), GEVIs, or ECoG electrode arrays.^94,95^ ECoG recordings usually have better temporal but poorer spatial resolution than imaging methods^38^ and can be hard to interpret, owing to volume conduction and a lack of cell-type specificity. Imaging studies with VSDs or GEVIs can monitor ~100-mm^2^ areas at spatial resolutions >10-fold finer than those of dense ECoG electrode arrays.^44,45^ However, VSDs lack cell-type specificity and are often highly prone to photobleaching and phototoxicity.

The TEMPO mesoscope reveals traveling voltage waves in targeted cell types in a manner infeasible with electrode arrays, which sample discrete sets of points, or prior imaging methods, which lack sensitivity to the brain’s intrinsic high-frequency rhythms. A recent GEVI imaging study used large-amplitude sensory stimulation to evoke high-frequency neural oscillations at the same frequency,^78^ but this differs markedly from our observations of internally generated beta and gamma waves. Demonstrating its potent capabilities, the TEMPO mesoscope captured CFC, enabled pathway-specific recordings, uncovered that hippocampal beta oscillations are traveling waves, and revealed bidirectional propagation of hippocampal waves in the theta- and beta-frequency bands.

### CFC in targeted neuron types

Past studies of CFC, including in humans, examined field potentials, aggregating contributions from multiple cell types. Other studies assessed CFC via joint LFP and extracellular recordings of spiking.^96–100^ Here, TEMPO revealed CFC in the transmembrane potentials of genetically identified cell types, potentially reflecting complex interactions between excitatory and inhibitory inputs and spiking outputs. We observed interactions in PV cells between neocortical delta and gamma rhythms in anesthetized mice, and between hippocampal theta and gamma oscillations during locomotion. The former gamma waves traveled lateral to medial, orthogonal to their coupled delta waves traveling anterior to posterior, showing that coupled waves may travel in distinct directions. Further cell-type-specific studies of CFC with TEMPO will facilitate mechanistic investigations.

### Traveling voltage waves in visual cortex

We characterized anesthesia- and visually-evoked gamma waves in visual cortex. Individual gamma waves traveled mainly unidirectionally, whereas the distribution of propagation directions was nearly isotropic in awake but not anesthetized mice (Figures 4P–4R and 5R–5T). This is suggestive of distinct propagation mechanisms^101^ and how the repertoire of neural dynamics varies with brain state.^102,103^ In primate visual cortex, gamma waves exhibited heterogeneous propagation directions in electrical recordings.^104,105^ Here, visually evoked gamma activity varied between cell types, with L4 pyramids showing less activation than L5 pyramids and other neuron classes (Figure S7A). We also observed 3–7 Hz waves at visual stimulus offset. These oscillations likely require thalamocortical interactions and may be precursors of human alpha rhythms.^57,58^ Prior electrical studies of 3–7 Hz oscillations examined cell-type differences one neuron at a time^56^ or used ECoG electrode arrays,^106^ which yielded similar wave speed estimates as ours.

Dual-cell-type TEMPO studies of 3–7 Hz waves revealed pyramidal cell activation preceding that of PV cells by ∼2 ms. This estimate was unchanged when we swapped the GEVI assignments; however, it was important to account for the voltage-dependent kinetics of the FRET-opsin GEVIs, which, unlike ASAP3, were insufficiently fast to report the wave’s instantaneous phase. FRET-opsin signaling delays differed between cell types; since GEVI time constants vary with transmembrane potential,^1,2,8,55^ cell-type differences in resting potentials or oscillatory amplitudes will induce cell-type-dependent GEVI kinetics and signaling delays. While further GEVI engineering will surely yield improved GEVIs, users studying timing relationships between cell types should carefully characterize GEVI-induced signaling delays within the oscillations of interest.

### Hippocampal theta and beta oscillations are bidirectional traveling waves

Using both TEMPO instruments, we studied CA1 hippocampal PV and pyramidal neurons and found locomotor-evoked theta (Figures 1D–1I, 3M–3O, 6K–6N, and S3C–S3E), beta (Figures 1D–1I, 3M–3O, 6A–6J, S2J–S2L, and S3C–S3E), and gamma (Figures 1E–1I, 2G–2J, 6L, and S3E) activity. In both neuron types, theta oscillations were coherent with the LFP across hippocampal laminae. For beta and gamma rhythms, coherence with the LFP was strongest in stratum radiatum for both cell classes. In PV cells, nested gamma activity arose near theta oscillation peaks, a well-documented form of theta-gamma coupling in electrical studies.^107^ PV cell beta activity was not similarly nested; although locomotion activated theta and beta rhythms, their moment-by-moment amplitudes seemed to vary unrelatedly (Figure 1D).^108,109^ Theta and beta waves also had distinct speeds and propagation directions (Figures 6J and 6N).

PV cells had especially prominent beta activity, which LFP recordings captured but not as strongly as TEMPO. Several factors likely influence these results. PV cells are relatively sparse and have modest dendritic trees and dipole moments, implying that LFPs may be less sensitive to their dynamics than to denser neurons with larger dendrites. PV cells may also exhibit beta and theta oscillations in different proportions than neuron types, such as pyramidal cells, that strongly influence LFPs. Cell-type differences in GEVI labeling and how oscillations engage subcellular compartments may also be important. In PV cells, GEVI labeling near our optrodes seemed largely in axons; in pyramidal cells, basal dendrites nearby the optrodes were well labeled. Further, unlike for theta activity, detection of beta activity was more sensitive to location along the CA1-to-CA3 axis (Figures S7B and S7C) and an electrode’s laminar position (Figure 3M); hence, slight variations in electrode positioning can impair detection of beta far more than of theta activity.

While hippocampal beta oscillations have been reported,^108–110^ we uncovered that they are waves traveling in two orthogonal directions, CA1 to CA3 and septal to temporal. Hippocampal beta activity arises during exploration of novel environments,^109^ olfactory learning,^110–112^ and memory retrieval,^113^ pointing to its behavioral relevance.

We imaged traveling theta waves in hippocampal PV interneurons and LS-projecting CA1 pyramidal neurons, initiating pathway-specific recordings of oscillations. Theta waves in LS-projecting neurons traveled approximately CA1 to CA3, consistent with electrophysiological studies.^71,87^ Unexpectedly, in PV cells, theta waves traveled in this and the reverse direction, showing that there are two classes of theta waves and suggesting LS-projecting neurons might participate selectively in CA1- to-CA3 theta waves.

Overall, these demonstrations that hippocampal beta and theta waves each come in two classes with distinct travel directions raise important questions about the participating cell types, mechanisms, and likely distinct signaling roles of waves traveling in each direction, as similarly discussed for neocortical oscillations.^106,114^

### Limitations of the study

By measuring transmembrane rather than extracellular potentials, TEMPO complements LFP, ECoG, and electroencephalogram (EEG) recordings. Unlike TEMPO, LFPs are affected by volume conduction, tissue composition, and laminar organization, complicating interpretations.^38^ We interpreted TEMPO signals as voltage-related only if they were coherent with LFP signals above levels observed for reference signals. This criterion could disqualify certain bona fide voltage signals, e.g., from sparse neuron types or asynchronous synaptic dynamics poorly captured by the LFP. Alternatively, weak oscillatory signals might emerge only after trial averaging, which could mask LFP coherence. However, interpreting TEMPO signals incoherent with the LFP is risky. Convolutional filtering effectively unmixes physiological and other noise artifacts, including those with non-stationary amplitudes. However, the filter has a stationary form and may not capture artifacts with time-varying spectral properties. This may leave residual noise in the unmixed voltage trace that is likely incoherent with the LFP.

As with field potentials, there are complexities relating to where TEMPO signals originate within neurons. The net signal is summed across all compartments of all GEVI-labeled cells in the recording volume, weighted by their GEVI-labeled membrane areas and proximities to the objective lens or fiber-optic. While dendrites and axons typically dominate a neuron’s membrane area, membrane labeling depends on GEVI trafficking, influencing the contributions to the TEMPO signal from these compartments. Further, fluorescence from neural compartments far from the collection optic will be attenuated more than emissions from nearby. The lack of optical sectioning in our instruments also obscures the spatial origins of the collected photons.

While TEMPO reports aggregate dynamics across a targeted cell population, as with LFP recordings one cannot distinguish large voltage changes in a modest set of cells from modest changes in a large cell set. We favored ASAP3 due to its sensitivity to subthreshold activity, but this does not exclude the possibility of spiking contributions to the TEMPO signal. Finally, since GEVIs reside in the cell membrane, alterations to transmembrane potential dynamics are conceivable, especially at high expression levels.

### Technological outlook

TEMPO will benefit from advances of several kinds. While GEVI engineering has mostly optimized spike detection, GEVIs designed for subthreshold voltages, such as the recently developed ASAP5,^115^ could improve TEMPO’s sensitivity and voltage-signaling fidelity. While uSMAART minimizes mode-hopping noise, autofluorescence fluctuations could be alleviated with autofluorescence-free fiber-optics and fluorescence lifetime-based signal separation.^47,116^ For TEMPO imaging, sCMOS sensors with more pixels, larger well capacities, and faster acquisition speeds will enable broader FOVs, improved sensitivity, and finer temporal snapshots.

TEMPO could also have advanced implementations. uSMAART might use high-density multi-fiber probes to study multiple brain areas concurrently.^49^ TEMPO imaging could reach deep tissues via microprisms^117^ or microendoscopes.^118^ Viral vectors with engineered capsids^106,107^ and cell-type-specific enhancers^119^ should broaden TEMPO’s utility for studies across cell classes and species.

Both uSMAART and the TEMPO mesoscope are compatible with fluorescent reporters beyond GEVIs. While Ca^2+^ indicators have high dynamic ranges, neuromodulator and neuropeptide indicators often produce smaller signals^51,91^; thus, studies with the latter should benefit greatly from the instruments and unmixing methods introduced here.

### Scientific outlook

TEMPO is poised to significantly impact basic and disease-related neuroscience. The neurons and mechanisms that drive many normal or pathophysiological rhythms remain unknown or under debate,^35–37^ impeding understanding of how clinical electrophysiological recordings reflect specific neuron types. Combined TEMPO and electrophysiological recordings will clarify which cell or projection types are active in normal or pathophysiological brain states and the frequency bands in which each neuron type is active.

Such joint recordings may benefit from transparent graphene-based electrodes^120,121^ that do not occlude optical FOVs. With distinct GEVIs for signaling supra- and subthreshold activity, neuroscientists could dissect how spiking and subthreshold dynamics in each neuron type relate to extracellular potentials. Dual-cell-class TEMPO will help reveal interactions between neuron types. Overall, these efforts should yield advances in understanding the biophysics of electric field generation and electrophysiological biomarkers of brain disease.

TEMPO has already revealed distinctive wave phenomena and can empower investigations of how waves influence neural computation. Traditional neural network models emphasize network topology, expressed via a synaptic weight matrix.^122,123^ The possible role of waves in neural computation^95,124^ suggests the geometric aspects of network connectivity may also shape information processing. By reporting rhythms up to ∼100 Hz, TEMPO will reveal contributions of different neuron types to the brain’s high-frequency waves and oscillations and their roles in health and disease.

## RESOURCE AVAILABILITY

### Lead contact

Further information and requests for resources and reagents should be directed to and will be fulfilled by the lead contact, Mark J. Schnitzer (mschnitz@stanford.edu).

### Materials availability

Plasmids for the 7 viruses generated in this study are available on Addgene (see key resources table). The transgenic reporter mouse line created for this work, Ai218, is available at Jackson Laboratory (https://www.jax.org/strain/037940).

### Data and code availability

- TEMPO datasets are available at DOI: https://doi.org/10.25740/cj061qr5050 and are publicly available as of the date of publication.
- Software for convolutional filtering and unmixing is available at https://github.com/schnitzer-lab/CoReU and DOI: https://doi.org/10.5281/zenodo.15686223 and is publicly available as of the date of publication.
- Software to process fiber-optic TEMPO recordings is available at https://github.com/sihaziza/uSMAART_public and DOI: https://doi.org/10.5281/zenodo.15679319 and is publicly available as of the date of publication.
- Software to process TEMPO imaging data is available at https://github.com/schnitzer-lab/TEMPO-processing/tree/release-HCh25 and DOI: https://doi.org/10.5281/zenodo.15686336 and is publicly available as of the date of publication.
- Any additional information required to reanalyze the data reported in this paper is available from the lead contact upon request.

## STAR★METHODS

### EXPERIMENTAL MODEL AND STUDY PARTICIPANT DETAILS

#### Mice

The Stanford APLAC approved all animal procedures. We used 3- to 12-week-old male and female C57BL/6, Thy1-GFP, PV-Cre, SST-Cre, Cux2-Cre^ERT2^, Nr5a1-Cre and Rbp4-Cre driver lines from Jackson Laboratory (C57BL/6: #000664, Thy1-GFP: #007788, PV-IRES-Cre: #008069, Sst-IRES-Cre: #013044, and FVB-Tg(Nr5a1-cre)2Lowl/J: #006364) and the Allen Institute (Cux2-Cre^ERT2^; MMRRC stock #032779-MU^125^ and Rbp4-Cre KL100; MMRRC stock #037128-UCD). For optical experiments using the Ai218 reporter line, we crossed it to either PV-Cre or Cux2-Cre^ERT2^ mice, yielding Cre heterozygous and heterozygous Ai218 reporter mice, *i.e.*, *Pvalb-IRES-Cre/wt; Ai218/wt* or Cux2-Cre^ERT2^*/wt; Ai218/wt*. We housed mice in normal light cycle conditions, 2–5 per cage before surgery, and 1 per cage afterward.

#### Transgenic mouse creation

To co-express the FRET-opsin voltage sensor Ace-mNeon1^2^ and the reference fluor mRuby3^82^ in genetically targeted cell-types, we generated a Cre-dependent Ai218 reporter mouse line expressly designed for TEMPO studies (Figure S6). We used our published Flp-in approach^83,85^ and constructed a targeting vector via gene synthesis and standard molecular cloning methods. We used an engineered mouse embryonic stem cell line containing Flp-recombinase sites paired with the constructed TIGRE target vector containing the following components^83–85^: FRT3 – 2X HS4 chicken beta globin insulators – TRE_tight_ promoter – LoxP – ORF-3X stops -Synthetic pA– hGH poly(A), PGK polyA) – LoxP – Ace2N-4AA-mNeonGreen-TS/ER – WPRE-bGH poly(A) – 2X HS4 chicken beta globin insulators – CAG promoter – Lox2272 – ORF-3X stops -Synthetic pA – hGH poly(A), TK poly(A) – Lox2272 – mRuby3– IRES2– tTA2 – WPRE – bGH poly(A) – AttB – PGK promoter – Hygro1-SD.1 – FRT5– origin– Amp (Figure S6A). Here, TS/ER denotes the Golgi export trafficking signal (TS) and the endoplasmic reticulum (ER) export sequence, as described.^2^

To express Ace-mNeon1 and mRuby3 in targeted neuron-types, we crossed Ai218 reporter mice with cell-type specific Cre-driver lines, either PV-Cre or Cux2-Cre^ERT2^. To verify the genotypes, we performed the polymerase chain reaction (PCR) on mice tail tissue samples. To activate the Cre-recombinase in crosses with the Cux2-Cre^ERT2^ mouse line, we intraperitoneally injected tamoxifen (T5648, Sigma), dissolved in corn oil (C8267, Sigma) at 20 mg/mL, when mice were 5–8 weeks old. Each mouse received 75 mg/kg of tamoxifen daily for 5 consecutive days.

#### Viral vectors

We obtained the following plasmids: pcDNA3-mRuby2^126^ (#40260, Addgene), pNCS-cyOFP^54^ (#74278, Addgene), pAAV-CaMKII-Ace2N-4AA-mNeon^2^ (hereafter termed Ace-mNeon1), pAAV-Syn-VARNAM^8^ (hereafter termed Varnam1; #115554, Addgene), pAAV-CaMKII-Varnam2^1^ (#195527, Addgene), pAAV-CaMKII-Ace-mNeon2^1^ (#195526, Addgene) and pAAV-EF1α-DIO-ASAP3-WPRE^55^ (#132318, Addgene).

We subcloned the genetic sequence encoding the reference fluor, cyOFP, flanked by BamHI and HindIII targeting sequences for the associated restriction enzymes, into an AAV backbone with the CAG promoter followed by the WPRE-hGH PolyA sequence. We used the same strategy for the reference fluor mRuby2. We cloned sequences encoding the fluorescent GEVI proteins Varnam1, Varnam2, or Ace-mNeon2 into the AAV backbone pAAV-EF1α-DIO-Ace-mNeon1 by inserting the Varnam1, Varnam2, or Ace-mNeon2 sequence in place of that for Ace-mNeon1 between NcoI and AscI targeting sequences, respectively. We cloned the ASAP3 sequence flanked with NheI and AscI into an AAV backbone with the CaMKII promoter followed by the WPRE-hGH PolyA sequence. We confirmed that vectors had accurate sequences using Sanger sequencing (Quintara Biosciences). We used QIAGEN Plasmid Plus Maxi Kits (#12963, QIAGEN) to amplify plasmids prior to viral packaging.

The Viral Tools facility at the HHMI Janelia Research Campus manufactured all viruses used in our work. For fiber-optic studies (Figures 1, 2, 3, S2, S3, and S7), we expressed green GEVIs using AAV2/PHP.eB-EF1α-DIO-ASAP3-WPRE (1.3E13 GC/mL), AAV2/PHP.eB-CaMKII-Ace-mNeon2-WPRE (7.48E13 GC/ml), AAV2/PHP.eB-Ef1a-DIO-Ace-mNeon2-WPRE (2.8E13 GC/ml) and AAV2/PHP.eB-CaMKII-Ace-mNeon1 (3.6E13 GC/mL). We expressed red GEVIs using AAV2/9-CaMKII-Varnam1-WPRE (1.8E13 GC/mL), AAV2/PHP.eB-EF1α-DIO-Varnam1-WPRE (1.3E13 GC/mL) or AAV2/PHP.eB-CaMKII-Varnam2 (3.6E13 GC/mL). We expressed reference fluors via AAV2/PHP.B-CAG-mRuby2 (2E14 GC/mL) and AAV2/PHP.eB-CAG-cyOFP (5.6E13 GC/mL).

For TEMPO microscopy studies (Figures 4, 5, 7, S6, and S7), we expressed a green GEVI with AAV2/PHP.eB-EF1α-DIO-ASAP3-WPRE (1.3E13 GC/mL), AAV2-Retro-CaMKII-ASAP3-WPRE (4.6E13 GC/ml), or AAV2/PHP.eB-CaMKII-ASAP3-WPRE (4.5E13 GC/ml), and/or a red GEVI with AAV2/PHP.eB-CaMKII-Varnam2 (3.6E13 GC/mL) or AAV2/PHP.eB-Ef1a-DIO-Varnam2-WPRE (1.18E13 GC/ml). To express reference fluors, we used AAV2/PHP.B-CAG-mRuby2 (2E14 GC/mL) or AAV2/PHP.eB-CAG-cyOFP (5.6E13 GC/mL). For control studies in which neither fluor was a GEVI (Figure S7A), we used AAV2/PHP.eB-CAG-GFP (7.95E13 GC/ml) and AAV2/PHP.B-CAG-mRuby2.

As described previously,^2^ all sequences for red and green FRET-opsin GEVIs, but not for ASAP3, included the endoplasmic reticulum (ER) export sequence and Golgi export trafficking signal (TS), as in the Ai218 mouse reporter line described above.

#### Pharmacology

For studies of ketamine-xylazine-anesthetized mice (Figures 2, 4, S3, and S6), we jointly dissolved ketamine (#50989–996-06, VEDCO) and xylazine (59399–110-20, AnaSed) in phosphate buffered saline (PBS; 10 mg/mL ketamine; 1 mg/mL xylazine), yielding final dosages of 100 mg/kg and 10 mg/kg, respectively. For studies of epileptic mice (Figure 2), we used kainic acid monohydrate (#K0250–10MG, Sigma-Aldrich). We dissolved kainic acid in NaCl (5 mg/mL), yielding a final dosage of 10 mg/kg. We injected these drugs intraperitoneally (i.p.) with a 30-gauge needle at least 20 min before data acquisition, unless noted otherwise.

### METHOD DETAILS

#### Instrumentation for fiber-optic TEMPO measurements

The uSMAART (**u**ltra-**S**ensitive **M**easurement of **A**ggregated **A**ctivity in **R**estricted cell-**T**ypes) fiber photometry system has 4 main modules: the (1) low-noise illumination module; (2) decoherence module; (3) low-noise fluorescence sensing module; and (4) phase-sensitive demodulation module.

##### Low-noise laser illumination module

To achieve stable illumination for uSMAART (∼0.0025% r.m.s. fluctuations in the amplitude of illumination modulation over the 25–100 Hz range), we selected high-frequency-modulated solid-state laser sources for their low noise properties (Figures S1A and S1B). Notably, commercial solid-state lasers commonly have an analog modulation bandwidth that extends to the ∼100 kHz range, much beyond the modulation bandwidths of typical LEDs. We modulated our lasers at 50 or 75 kHz, to perform measurements in a part of the frequency spectrum where both lasers and detectors have reduced instrumentation (1/*f*) noise (Figure S1C). Normally, the blue and green lasers were modulated at 50 and 75 kHz, respectively, using a purely sinusoidal analogue modulation scheme to ensure that no spectral harmonics propagated to adjacent recording channels.

To attain low-noise illumination from a laser source, it is important to minimize optical back-reflections into the laser cavity, which can lead to unstable emission output levels. To prevent back-reflections, we placed a Faraday isolator (–40 dB attenuation) immediately succeeding each laser source in its illumination pathway (Figure 1A). Given these considerations, we used continuous wave laser light sources with 488 nm and 561 nm wavelengths (488–20LS and 561–50LS OBIS Lasers, Coherent) to excite green and red fluorescence, respectively. We used a fixed free-space Faraday isolator (IO-3–488-HP, Thorlabs) and a tunable free-space isolator (FI-500/820–5SV, Linos) to prevent back-reflections into the two respective laser cavities. To further reduce back-reflections, we coupled all laser beams into an 8°-angled fiber-optic patch-cord (FC/APC patch cord in Figure 1A).

To align the polarization of the 561-nm-emissions with the polarization axis of the accompanying isolator, we aligned an achromatic half-wave plate (AHWP05M-600, Thorlabs) in front of the isolator’s entrance face. We aligned the two laser beams onto a common optical path using steering mirrors (BB1-E02, Thorlabs) and iris diaphragms (CP20D, Throlabs). To direct 10% of the light from each laser beam to a photodiode (PDA100A, Thorlabs) for continuous monitoring of the emission power, we used a pair of 90/10 beamsplitters (BS028, Thorlabs). We combined the other 90% of the light power from each of the two beams using a dichroic mirror (FF511-Di01, Semrock). We coupled the pair of collimated beams into a multi-mode, 8°-angled fiber (105 μm core, 0.2 NA; FG105LCA, Thorlabs) with an FC/APC fiber collimator (F240APC-532, Thorlabs).

##### Decoherence module

The decoherence module breaks the coherence between light in different spatial (transverse) modes within the optical fiber (Figure 1A), rendering the illumination pattern and output power insensitive to motion of the multimode fiber connected to the animal. Without this coherence reduction, fiber motion would induce optical mode-hopping fluctuations, a form of optical speckle (Figures S1D and S1E).^46^ To break the illumination coherence, we built a dual-stage optical diffuser, the second stage of which is dynamic and reduces the spatial coherence of the beam by inducing rapid variations of the optical path length across each beam’s spatial cross-section. Within the decoherence module, after exiting the FC/APC optical fiber, each laser beam passes through an axicon lens, which converts each beam’s Gaussian cross-sectional profile to an approximate flat-top intensity profile (Figure 1A). This increases the number of micron-scale grains within the diffuser that interact with and scatter the laser light.

To implement this approach, we first collimated the laser light exiting the FC/APC fiber module using a fiber-optic collimator (F240APC-532; Thorlabs). Next, we positioned onto the light path a Ø1” plano-convex ring grade axicon lens (140° apex angle; #83–790, Edmund optics). The axicon focused each beam onto the dual static-dynamic diffuser (LSR-3005–24D-VIS, Optotune), which had an average grain size of 3 μm. The second diffuser in the pair circularly oscillated at 300 Hz in the two lateral dimensions orthogonal to the longitudinal optical axis, actuated by four independent electro-active polymer electrodes. To collimate the diffused laser light, we used an aspheric lens (A240TM-A, f = 8.00 mm, NA = 0.5, Thorlabs). Finally, to maximize the photon collection efficiency, we coupled the beams into a FC/PC multimode fiber Ø1000 μm / 0.39 NA (FP1000URT, Thorlabs) using a fiber collimator (F240PC-532, Thorlabs). To enable concurrent recordings in two brain areas (Figure S3), we coupled the illumination into a FC/PC bifurcated multimode fiber bundle, Ø400 μm / 0.39 NA, (BFY400HF2, Thorlabs), using a FC/PC to FC/PC mating sleeve (ADAFC2, Thorlabs).

In sum, owing to the decoherence module, the noise reductions achieved in the low-noise illumination module were impervious to fiber and animal motion (Figures S1D and S1E).

##### Fluorescence collection module

The collection module maximizes fluorescence signals while minimizing noise (Figure 1A). We computed, for multiple commercial filter sets, the expected collection efficiencies and levels of crosstalk between fluorescence detection channels, based on the spectral properties of all fluorophores used in our experiments. We thereby selected filter sets for which bleedthrough of emissions from the reference fluor into the detection channel for the GEVI was <0.01%, minimizing the addition of photon shot noise from reference fluor emissions into neural voltage measurements.

To maximize signal-to-noise levels of fluorescence detection, we selected an avalanche photodetector with a noise-equivalent power of 2.5 fW/√Hz and a signal detection bandwidth (100 kHz) wide enough to accommodate the two lasers’ analogue modulation frequencies (50 and 75 kHz).

To deliver light to and from the brain, we chose an optical fiber of low intrinsic autofluorescence. Minimizing autofluorescence is vital, as otherwise it will diminish a detector’s effective dynamic range; further, autofluorescence photon shot noise can mask the weak signals associated with high-frequency voltage dynamics. To further minimize fiber autofluorescence, we photobleached the fiber conveying light to and from the brain at least 1 day before each experiment using the same illumination wavelengths, yielding a ∼5–10-fold reduction in autofluorescence (∼1–10 pW residual fiber fluorescence induced by ∼100 μW of 488 nm illumination).^127^ We removed light in the cladding modes of the fiber delivering light to and from the brain by winding the fiber ten times around a mandrel. In many mice, we also measured local field potential (LFP) signals near the tip of the optical fiber (Figure 1A).

In our specific implementation of these approaches, the fluorescence collection pathway had two spectral detection channels, and a single-band dichroic mirror and two bandpass filters to separate and steer green and red signals to separate photodetectors. The excitation laser beams from one branch of the bifurcated multimode fiber bundle were collimated using a fiber collimator (F240PC-532, Thorlabs), reflected off a dual-edge dichroic mirror (Di01-R488/561, Semrock), and focused into a multimode, low autofluorescence, pre-photobleached, fiber-optic patch cord (1.5-m-long, Ø400 μm-core, 0.50 NA; FT400URT, Thorlabs) using a fiber collimator (F240PC-532, Thorlabs). The patch cord fiber was tightly wrapped ten times around a Ø1” pedestal post (RS3P8E, Thorlabs), placed just after the fiber collimator, which served as a mandrel wrap and provided about a 3 dB (or ∼50%) reduction in the illumination fluctuations at the specimen when the optical fiber was moving owing to movement of the animal. Using a ceramic mating sleeve (ADAF1, Thorlabs), we connected the patch cord to an optical fiber (CFM14L05, Thorlabs) that we implanted in the mouse brain (cf. Mouse Preparation). The total mean power delivered to the brain was 25–200 μW. Fluorescence emissions from the brain passed through the dual-edge dichroic mirror and were split into red and green channels using a single-edge dichroic mirror (FF562-Di01, Semrock). Light in each channel passed through a bandpass filter (FF01–520/28 or FF01–630/92, Semrock) and was focused onto an avalanche photodiode (APD) (APD440A2, Thorlabs) by an aspheric lens (A240TM-A, f=8.00 mm, NA=0.5, Thorlabs).

##### Phase-sensitive detection module

The detection module performs phase-sensitive frequency demodulation to unmix emission signals that are excited by different lasers but captured on the same detector. With this approach, one need not rely solely on emission filters to separate fluorescence from different fluors but can also exploit the fluors’ different absorption spectra. To demodulate the fluorescence signals from the photodetector outputs, we used a pair of low noise, digital lock-in amplifiers (LIA) (MFLI-MD, Zurich Instruments) (Figure 1A), one for each illumination path. Each LIA provides one analog modulation signal and can demodulate up to 4 signals.

We amplitude-modulated the 488 nm and 561 nm lasers with analogue sinusoidal oscillations at 75 kHz and 50 kHz respectively, with 0 V to 4 V peak-to-valley amplitudes. We used one LIA to demodulate signals from the photodiode measuring 488 nm laser power fluctuations and from the APD measuring green fluorescence signals. We used the other LIA to demodulate signals from the photodiode measuring 561 nm laser power fluctuations and from the APD detecting the red fluorescence. Signals were demodulated using a linear-phase finite impulse response (FIR) filter, which is specifically designed for digital signal processing applications and allows low-pass filtering of signals with a frequency range that depends on the demodulation frequency. The low-pass filter used for demodulation was set to have a 0.1 ms time-constant and 48 dB/octave roll-off, enabling a measurement bandwidth of 0–500 Hz.

For concurrent fiber-optic TEMPO recordings from two cell types (Figures 3, S2, and S3), cyOFP emissions were largely captured in the red fluorescence detection channel but demodulated at 50 kHz owing to their excitation by the 488-nm laser. We routed the reference modulation signal from the first LIA controlling the 488-nm-laser to the second LIA, to demodulate the signal from the APD capturing red fluorescence and thereby to extract the time-varying level of cyOFP fluorescence.

For concurrent measurements from two cell-types and two brain regions (Figure S3), we duplicated the fluorescence collection module. This approach yielded 4 independent APD signals, 2 per brain area. The LIA configuration was similar to that used for studies with a single GEVI, except that we no longer measured laser power fluctuations, thereby enabling each of the two LIAs to demodulate all 3 fluorescence signals collected by a single optical fiber.

By combining the set of 2 emission filters and 2 dichroic mirrors with the frequency modulation and demodulation scheme used in our phase-sensitive detection module, we obtained the following optical bleedthrough and crosstalk numbers for the uSMAART systems. Less than 0.01% of the collected fluorescence from the red fluor (either mRuby2 or Varnam2) enters the green fluorescence detection channel. Approximately 8.8% of the fluorescence collected from the green fluor ASAP3 (or ∼6.4% for Ace-mNeon1) enters the red fluorescence detection channel. However, owing to the frequency modulation, the red and green signals can be unmixed from each other, because they are excited by lasers modulated at distinct temporal frequencies. Therefore, virtually no green fluorescence (either GFP, Ace-mNeon1 or ASAP3) contributes to the demodulated signal from the red fluorescence detection channel, except for the added green fluorescence photon shot noise, which has a white frequency spectrum and cannot be removed through demodulation.

Nevertheless, in a dual-GEVI experiment, because cyOFP is excited with the same laser source as the green fluor, the bleed-through of green GEVI fluorescence into the red detection channel does affect the demodulated cyOFP signal. Further, 1.1% of the fluorescence collected from cyOFP bleeds into the green fluorescence detection channel. In addition, because cyOFP has a broad absorption spectrum, a certain amount of cyOFP fluorescence can be excited by the green laser used to excite Varnam. Consequently, when the green and blue laser intensities are set to equivalent levels at the specimen, ∼18.5% of the total cyOFP fluorescence collected in the red fluorescence channel cannot be removed through frequency demodulation and hence will appear as crosstalk in the demodulated Varnam2 signal. Conversely, ∼10.3% of the collected fluorescence from Varnam2 will appear as crosstalk in the demodulated cyOFP signal, because the blue laser excites both fluors. For these reasons, in dual-GEVI experiments, data analyses included a decrosstalking step (see below, data processing for fiber-optic TEMPO recordings)

#### Electrophysiological recordings

We performed electrical recordings concurrently with fiber-optic TEMPO (Figures 1, 2, 3, S2, and S3) and TEMPO imaging studies (Figures 6, S7B, and S7C). We amplified electrophysiological signals with a digital head-stage amplifier (RHD 16-channel or 32-channel, Part #C3334 or #C3324, Intantech) attached to our custom-made optrode (see below). We acquired digital data at a 2-kHz-sampling rate using a USB data acquisition board (RHD USB interface board, #C3100, Intantech).

#### Synchronization of optical and electrical recordings

To synchronize the optical and electrical recording instruments, we generated a synchronizing digital signal (hereafter called ‘syncTTL’), which comprised a square-wave with a 50% duty ratio and a 1-s-long ON-state but with intervals of random duration between the square pulses, to enable unambiguous temporal alignments. The syncTTL signal was externally and independently generated by a data acquisition system (USB-6003, National Instruments) and recorded concurrently by both the electrical and optical data acquisition systems.

To temporally align the optical and electrical signals, we first preprocessed the LFP signal as follows. (1) We removed any residual 60-Hz narrow-band noise, representing contamination from the electrical power line, by using an 8^th^-order Butterworth notch-filter; we designed the filter using the *designfilt()* function in MATLAB, with the option ‘bandstopiir’ and the low and high cut-off frequencies set at 59.5 Hz and 60.5 Hz, respectively. We then applied the filter using the zero-phase digital filtering function *filtfilt()*. (2) We bandpass-filtered the resulting LFP traces with a 3^rd^-order Butterworth filter with low and high 3 dB cutoff frequencies of 0.1 Hz and 500 Hz. (3) We interpolated the LFP traces and the squared syncTTL signal so the sampling rates of the resulting traces matched the sampling frequency of the optical data. We used the MATLAB function *interp1()* with the ‘spline’ method to interpolate the continuously valued LFP trace. To interpolate the binary valued syncTTL signal, we used the *interp1()* function with the ‘nearest’ method. Finally, we aligned the optical and electrical traces using the temporal registration provided by the concurrently recorded syncTTL signal.

#### Optical and optrode implants

For all uSMAART studies with a single GEVI, we implanted an optrode into the brain to allow joint optical and LFP measurements (Figures 1, 2, and S2), except for our recordings in the visual cortex of anesthetized mice (Figures 2A–2D), for which we only used an optical fiber. For TEMPO microscopy studies of hippocampus, we used a distinct but similar optrode for dual optical and electrical measurements (Figure 6). For uSMAART studies of visual cortex with two GEVIs (Figures 3 and S2), we implanted an optical cannula without an ipsilateral LFP electrode, except for Figures 2G–2I. However, for studies of hippocampus with two GEVIs, we implanted a linear silicon probe (A1×32-Edge-10mm-30–177-H32_21mm, Neuronexus) in the contralateral hippocampus. We manufactured all optical and optrode implants under a stereomicroscope (Leica MZ7–5) equipped with a soldering station.

For TEMPO mesoscope studies in the hippocampus (Figure 6), we manually fabricated each cannula implant using a borosilicate cover glass (170 ± 5 μm thickness, Schott) that was laser cut to a 3-mm-diameter circle (Fluence Technology). We glued the cover glass onto a 3-mm-outer-diameter, 1.5-mm-long stainless-steel ring (grade 316, gauge 11TW, MicroGroup) using adhesive cured with ultraviolet light (Loctite #3105). To record optical and electrical signals simultaneously, we glued two tungsten wires (0.002″, 99.95% CS, with a single polyimide insulation, M215580, California Fine Wire) to the optical implant, ∼50 μm beyond the glass window, using UV-light-cured adhesive (Loctite #3905). We used coated stainless-steel wire (0.005” bare, 0.008” coated, 100 feet, A-M Systems) for the ground electrode, and we soldered both the LFP and ground electrodes into a gold-plated pin head (0.031”, WPI). During the implant surgery, we positioned the ground electrode in the cerebellum.

Optrode implants for uSMAART comprised an optical fiber (400 μm core diameter, 0.5 NA, Thorlabs) (Figures 1, 2, 3, S2, and S3). Optrode implants for hippocampal imaging comprised a circular borosilicate cover glass (3 mm diameter, 170 mm ± 5 mm thickness, Schott) that was glued to the end of a stainless-steel ring (3 mm OD, 1.5 mm long) with UV-curable adhesive (Loctite #3105) (Figure 6). To achieve dual-modality, optical/LFP recordings (Figures 1, 2, 3, and 6), we affixed a pair of ∼1-cm-long, ∼50-μm-diameter tungsten wires coated with single polyimide insulation (M215580, California Fine Wire) to the optical fiber (Figures 1, 2, and 3) or cannula (Figure 6) at two different axial positions, spaced in increments of ∼50 μm, with the first tungsten wire placed flush with the optical fiber or window, and with the tip of the other wire extended into the brain tissue under investigation. This set of electrode placements was chosen to maximize the chances that at least one of the electrodes would provide high quality LFP signals, given that our surgical placement of the implant was performed without fine visual feedback regarding the exact location of the ∼100-μm thick pyramidal cell layer of CA1. We glued these tungsten wires onto the optical cannula with ultraviolet-light-cured adhesive, which constrained the electrodes to be laterally offset by 1.5 mm from the center of the imaging window. Next, we soldered a gold-plated pin-head (NC0102069, WPI) onto the end of each tungsten wire projecting away from the imaging window. The reference electrode comprised a ∼5-mm-long, ∼127-μm-diameter, uncoated, stainless steel wire (#791600, A-M Systems) soldered to a gold-plated pinhead (NC0102069, WPI). Separately, we fabricated a 4-channel-connector using 4 gold-plated sockets (NC1456862, WPI) and a connector board (EIB8, Neuralynx).

#### Mouse preparation for fiber-optic TEMPO measurements

Mice (aged 8–16 weeks at start) underwent two surgical procedures under isoflurane anesthesia (1.5%–2% in O_2_). In the first procedure, we injected viruses to express the GEVI and reference fluor. In the second procedure, performed about a week after viral injection, we implanted the optrode.

Coordinates for virus injections in CA1 (Figures 1, 2, 3, 6, S1–S3, and S7) were −2.0, 1.7, −1.1 (in mm from bregma; AP, ML, DV). Coordinates for virus injections in V1 (Figures 2, 3, S2, S3, and S7) were −3.5, 2.5, −1.2. Coordinates for injections of retrograde virus in the lateral septum (Figure 6) were 0.4, 0.4, −3.3. For single cell-type studies, we injected 0.5 μL per mouse of a solution containing 5E9 genome copies (GC) of the GEVI virus and 1E9 GC of the reference virus. For dual cell-type studies, we injected 0.5 μL per mouse of a solution containing 10E9 GC of the Cre-dependent GEVI virus, 5E9 GC of the CaMKII-dependent GEVI virus and 1E9 GC of the reference virus.

For all mice, we prepared the skull by manually removing the conjunctive tissue. We cleaned the skull by applying H_2_O_2_, quickly followed by rinsing with Ringer’s solution (#50980245, ThermoFisher). For studies in the primary visual cortex V1, we drilled a 0.5-mm-diameter opening in the skull above V1 using 0.5-mm-diameter micro drill burr (#19007–05, Fine Science Tools). For studies in the dorsal CA1 area, we drilled a 2-mm-diameter opening in the skull above CA1 using the same model of 0.5-mm-diameter micro drill burr. We removed cortical tissue above CA1 using suction applied via a 30-gauge blunt needle under ice cold Ringer’s solution. To improve optical access to hippocampal tissue, we gently removed two of the three layers of the overlying alveus. We discarded mice in which CA1 was damaged, as a damaged hippocampus is prone to undergo local epileptic seizures. We inserted the implant and sealed the gap between the skull and the implant using adhesive cured with ultraviolet light (Loctite 33105). In mice used for uSMAART studies under head-restraint (Figures 3, S2, and S3), we glued a custom-designed stainless steel head bar on the mouse skull and secured the implant to the skull using blue-light cured resin (Flow-it ALC, Pentron). To mitigate post-operative pain, we administered carprofen (5 mg kg^−1^) about 30 min prior to the end of surgery. Mice recovered for >2 weeks before imaging experiments began.

#### Fiber-optic TEMPO recording sessions

We performed all recordings during the mouse’s light cycle. In studies with ketamine-xylazine-anesthetized animals, mice were either unrestrained (Figure 2) or head-restrained (Figure S3). Recordings began ∼20 min after anesthesia administration, when the mice became immobile, and lasted 2–10 min. In studies of mice allowed to behave freely or of those with kainate-induced epilepsy, the animals were placed in a circular arena with clear acrylic walls (31 cm diameter, 31 cm height, Figure 1) or within their house cage (Figures 2 and 3); the recordings lasted <60 min. In some experiments (Figures 2 and 3), we took video recordings of the mouse’s behavior in the open field arena or home cage using infrared illumination and an overhead camera (DMK23 U40, Imaging Source) that acquired images at a 20 Hz frame rate. To control video acquisition, we used the IC-Capture software package (Imaging Source).

#### Visual stimulus presentation

We placed an LCD monitor (85-cm-diagonal) in the monocular visual field of the mouse at a distance of 20 cm, contralateral to the fiber optic implant (Figures 3, S2, and S3) or cranial window (Figures 5, 7, S6, and S7). We used custom software developed with the Psychtoolbox in MATLAB (MathWorks) to display drifting gratings (sine waves of 1 Hz temporal frequency, 0.033 cycle per degree of spatial frequency and 100% contrast, oriented at 315° to horizontal). We placed a blue bandpass optical filter (#5084 Damson Violet −48’ X 25’ Roll, Rosco E-Colour) onto the video monitor to ensure that its illumination was outside the range of wavelengths detected by the TEMPO apparatus. Presentations of the drifting grating lasted 1.5 s and were preceded and followed by presentations of a gray isoluminant screen. Inter-trial intervals (ITIs) were randomized between 2–5 s.

#### Behavioral studies of rest-to-run transitions

We built a custom mouse running wheel using a 3D-printed, polylactic acid plastic wheel (8 cm wide, 13 cm diameter). We covered the wheel’s surface with self-adherent wrap (Coban, 3M) and tracked the angular displacement using a rotary encoder (optical AB Phase Quadrature Encoder 600P/R, Amazon). We recorded the wheel displacements via a data acquisition device (BNC-2090A, National Instruments) controlled via LabView 2019 software (National Instruments). To elicit a rest/run behavioral transition, we delivered an airpuff to the mouse’s back. The airpuff intensity was ∼30 psi and was administered via a 20-gauge blunt needle placed 1–2 cm above the mouse’s back. We triggered the airpuff after the mouse stayed immobile for at least 5 s. Each experimental session lasted for about 5 min. All mice were habituated on the running wheel for at least 2 days, for at least 15 min per day, before TEMPO recordings began (Figures 6 and S2).

#### TEMPO mesoscope

The mesoscope system includes an LED illumination module, an objective lens with an electronically controlled focusing mechanism, a fluorescence filter module, a tube lens system that allows differential focusing between the two fluorescence channels, a pair of sCMOS cameras, data acquisition and system control electronics, as well as a custom mechanical design (Figure 4).

We developed the TEMPO mesoscope to monitor neural voltage dynamics across wide imaging fields with high spatiotemporal resolution and detection sensitivity. (Our system has a nominal field-of-view of 8 mm, although, in practice, we used glass cranial windows of 7–8 mm in diameter.) The imaging system we designed and built has several distinctive advantages, including a 0.47 numerical aperture (NA) objective lens, which enables good fluorescence collection across the large field-of-view and provides high efficiency (∼85%) transmission of visible wavelengths. The system’s high-speed imaging capabilities allow recordings at 100 fps across the full field-of-view and even faster recordings over a reduced field-of-view of width, $FOV_{y}$, at a frame rate of 100 fps × 8 mm/$FOV_{y}$. For example, to study gamma activity we typically acquired images at 130–300 fps; the images taken at ∼130 fps were of sufficient spatial size to cover the full area of visible brain tissue through a ∼7-mm-diameter cranial window (e.g., Figure 4H), whereas we used 300 fps image acquisition to capture in detail the propagation of individual gamma waves (e.g., Figure 4O). The optical apparatus has a resolution of approximately 6 μm and two fluorescence detection channels that use a custom-made, polished dichroic filter (λ/4 peak-to-valley wavefront errors, Alluxa) to separate green and red fluorescence. This combination of features has generally been unavailable in commercial or previously published microscopes yet was vital for the recordings of this paper.

Past work on large-scale voltage imaging focused on slow (<10 Hz) voltage activity and used imaging systems that supported the detection of voltage activity in this frequency range.^128,129^ During the optical design process, we sought a design that maximized the SNR, imaging frame-rate, and field-of-view for studies of voltage dynamics up to the high-gamma range. We also sought recordings up to 60 min, without photodamage to tissue or undue photobleaching. To obtain SNR values sufficient to detect the tiny fluorescence changes associated with fast voltage dynamics (*i.e.*, <0.1% ΔF/F), we sought photon shot-noise-limited measurements such that the mean flux of signal photons would be much greater than accompanying photon shot noise. In practice, the goal of maximizing the photon flux implies that the cameras should operate at close to saturation.

Given this general approach, we examined the SNR for detecting a voltage wave of spatial wavelength $\Lambda_{\gamma }$ and temporal frequency $f_{\gamma }$. For simplicity, we considered a wave propagating in one spatial dimension, A(x,t)=A(φ(x,t)), where the phase depends on space and time, $\varphi (x,t)=2\pi \left(x/\Lambda_{\gamma }-t/f_{\gamma }\right)$. We considered the SNR level needed to detect the wave in a single trial (*i.e.*, without trial averaging) and quantify its propagation properties. Assuming there is a spatially uniform noise of standard deviation, $\sigma_{\epsilon }$, originating mainly from photon shot noise, the ability to distinguish changes in the wave’s amplitude in space and time via two measurements is related to the local phases of the wave and to the desired SNR:

$$SNR\left(\varphi_{i},\varphi_{j}\right)=\left|\langle A\left(\varphi_{i}\right)\rangle -\langle A\left(\varphi_{j}\right)\rangle \right|/\sigma_{\epsilon },$$

where $\varphi_{i}$ and $\varphi_{j}$ are the phases of the wave for the two measurements in pixels i and j. Similarly, the ability to detect the wave relates to the SNR with which one can distinguish between the polar-opposite phases:

$$SNR(0,\pi )=\text{Δ}A/\sigma_{\epsilon },$$

where $\text{Δ}A=|\langle A(0)\rangle -\langle A(\pi )\rangle |$. For waves at the edge of detectability, it is beneficial to work at the Nyquist limit of the wave and to boost the SNR by averaging fluorescence signals across as many pixels as possible within a correlation length, which can be estimated as half a wavelength, $\Lambda_{\gamma }/2$. (Averaging over pixels beyond this correlation length would lead to canceling out of the wave amplitudes.) The number of photoelectrons acquired from this width, $\Lambda_{\gamma }/2$, of tissue, within $n_{f}$ image frame acquisitions relates to the corresponding number of camera pixels and their individual well capacity, $W_{C}$, assuming the camera is operating close to saturation. Given a system magnification, M, and pixel pitch, $I_{px}$, the maximum number of photoelectrons that can be acquired is thus:

$$n_{max}\leq {\left(M\Lambda_{\gamma }/2I_{px}\right)}^{2}n_{f}W_{C}.$$

This sets the shot noise in the photoelectron count as $\sigma_{\epsilon }≅\left(M\Lambda_{\gamma }/2I_{px}\right)\times \sqrt{n_{f}W_{C}}$. For a wave of maximum fluorescence amplitude $\frac{\text{Δ}F}{F}$, the maximum SNR thus is bounded as follows:

$$SNR\leq \frac{\text{Δ}F}{F}\frac{M\Lambda_{\gamma }\sqrt{n_{f}W_{c}}}{2I_{px}}.$$

Optimization of this expression leads to high magnifications and the use of many pixels of large well capacity, which is opposite to the approach taken in most prior voltage-imaging studies in which the dorsal neocortex was imaged at low magnification in order to obtain fast imaging frame rates.^129^ Further, with our sCMOS cameras, one can use a high sampling frequency, f, to maximize $n_{f}$, at the expense of the vertical field-of-view, $FOV_{y}$. Similarly, the magnification limits the field-of-view, suggesting that major gains in SNR are attainable in exchange for working with limited fields-of-view and large pixel counts of large well capacity. (Figures S4H–S4J has plots showing how the SNR scales with magnification and pixel well capacity at a constant illumination power and field-of-view.)

Given the above considerations, we decided to build a microscope whose imaging field can span our 7–8 mm cranial window preparation. Given this field-of-view size, we sought to optimize the other properties noted above that influence detection sensitivity, and we chose fast and high-quantum efficiency sCMOS cameras with sensor chips 13–15 mm across. We conducted optical design studies with ZEMAX software. We modeled the objective and tube lenses in an infinity-corrected configuration and aimed to optimize the resolution and transmission across the field-of-view, which led to the minimization of vignetting and aberrations while keeping photon transmission high.

During optical design, we examined the properties of both custom lenses and commercially available optical components. After an iterative optimization process that considered both the properties of the objective lens and its performance in conjunction with the tube lens, we selected two existing lenses for high-end photography. The Leica Noctilux-M 50mm f/0.95 ASPH (NA=0.47) serves as our system’s objective lens, and the Canon EF 85mm f/1.2L II USM serves as the tube lens (85 mm effective focal length) in each spectral channel. This pair of lenses provides a net optical magnification of about 1.7×, a resolution of about 6 μm, and visible light transmission of ∼85% at 532 nm in the absence of any filters.

To accompany the objective and tube lenses, we designed an illumination module to provide high-power, high-stability incoherent illumination across the absorption spectrum of our green and red fluorescent GEVIs. Illumination from two LEDs (UHP-T-475-SR and UHP-T-545-SR both with low-noise and detached-fan options, Prizmatix) was combined using a dichroic beam combiner (T505LPXR, Chroma) installed in a beam combiner module (Prizmatix). The light then passed through a dual-bandpass clean-up filter (FF01–482_563, Semrock). A dual-band custom dichroic (488/568-Di, Alluxa), 70 mm × 100 mm in size, reflected illumination from both LEDs and transmitted the fluorescence. In dual cell-type experiments, we directly monitored the LED illumination power using photodiodes.

A motorized stage (Sutter MP-285) allowed us to perform fine axial focus adjustments of the objective lens. We created a custom mechanical assembly to mate the lens with the focusing stage and to allow a high mechanical accuracy of insertion and alignment with the rest of the optical axis. An additional differential focusing mechanism relied on the internal focusing mechanism of the Canon 85 mm f/1.2 tube lenses, controlled by an Ultrasonic Motor (USM) Autofocus (AF) controller (Astromechanics) via the serial port. Each USM focus controller had a EOS-C-mount adapter that allowed alignment between the lens and the camera. Alignment of the tube and objective lenses for the green channel was achieved using a CNC-machined filter cube holder (Figure 4); residual angular misalignment between the two video streams was corrected computationally. Fine alignment of the red-channel tube lens/camera module was implemented using a 5-axis compact kinematic stage (Newport 9081-M), placed at the base of the camera.

The microscope’s emission pathway was designed to separate the illumination from the fluorescence emissions in both spectral channels, while preserving the optical resolution in both transmission and reflection. In the emission path, we used a custom-made 550 nm short-pass dichroic mirror (Alluxa), placed on a super-flat substrate, such that the wavefront error in reflection was empirically 150 nm or less across the entire coated filter. We used emission filters specific to each color channel, a 520/41 nm for Ace-mNeon (Alluxa) and ASAP3 and 609/62 nm (FF01–609/62–25, Semrock) for VARNAM2. With these filters, only 0.1% of the fluorescence collected from the red fluor (either mRuby or Varnam) enters the green fluorescence detection channel. Whereas ∼7% of the fluorescence collected from green fluorescent ASAP3 (∼9.5% for Ace-mNeon1) enters the red fluorescence detection channel. In experiments using cyOFP, ∼2% of its collected fluorescence enters the green fluorescence detection channel. During data processing, we computationally corrected for the bleedthrough of the green emissions into the red fluorescence detection channel (see section below, processing of TEMPO mesoscope video data).

We utilized scientific grade sCMOS cameras (Hamamatsu ORCA Fusion Digital CMOS camera; Hamamatsu Photonics K.K., C14440–20UP) as detectors. We controlled both cameras through the CoaXPress interface using a PCIe frame grabber and streamed the data through the same interface.

After having designed the optics, we designed the mechanical assembly using Inventor (Autodesk) computer-aided-design (CAD) software. The main components were custom-designed and machined on a 5-axis CNC mill (Optima Precision) using aluminum alloys 6060 and 7075 and then anodized black to reduce stray light from reflections.

Electronics for controlling the microscope and data acquisition are detailed in Figure S4. The cameras ran in external trigger mode, using the configuration of Figure S4C. For studies using a single GEVI, we used the External Start Trigger mode to initiate frame acquisition on both cameras. The cameras acquired frames in the overlap rolling shutter mode based on the internal pixel clock, which we verified gave temporal synchronization between the two cameras across recordings of up to 30 min. (We did not test longer time spans). This operation mode provided stable exposure durations as well as the fast data acquisition rates that our work required.

Figure S4 provides a schematic of the camera operations, including the trigger output signals used for diagnostics and control, such as VSYNC or Global Exposure signals. As Figure S4 shows, we controlled the system from a PC workstation utilizing PCIe and serial port interfaces. A DAQ board (myRIO-1900, National Instruments) equipped with a custom-programmed field programmable gate array (FPGA) (Xilinx Z-7010) controlled all analog and digital signals. The DAQ board providing the interface to the FPGA was controlled by the PC operating the mesoscope apparatus. Live data from each camera streamed to a RAID0 array of NVMe SSD (Samsung 970 Pro) hard drives on a NVMe RAID Controller with a PCIe interface (Highpoint Technologies Inc).

We stored data files as native Digital Camera Image files (DCIMG, Hamamatsu). We used Samsung NVMe 970 Pro M.2 storage with triple-cell base units and no buffering, which was crucial for achieving sustained write-in using consumer grade hard drives. For long-term storage of raw data, we used a QNAP NAS TS-1685, which hosts a RAID6 array of 140 TB of storage, with 2TB of SSD (Samsung 860) cache, connected to the acquisition computer via a direct 10 Gbps optical link.

In practice, we found that a suboptimal streaming configuration, even when using hard-drive interfaces that nominally exceed the requisite write-in speed, may lead to detrimental frame dropping. To prevent frame-dropping, we established a diagnostic routine based on the analysis of the time stamps natively written in the DCIMG files and decoded with custom software provided to us by Hamamatsu. The precision of the time stamps was in the microseconds range, allowing us to detect frames whose exposure interval was notably larger than the nominal period between image frames. With this approach, we verified several hard-drive configurations that provided a stable data streaming architecture for >1 yr. We observed that after ∼1 year of use, cells in NVMe memory can degrade, leading to a recurrence of frame dropping. This issue can be resolved by replacing the hard drives.

#### TEMPO imaging studies in two neuron-types

To track the emissions of 3 different fluorophores, we created a protocol based on the interleaving of illumination from the two different LEDs and our use of two spectrally distinct GEVIs plus a long Stokes-shift reference channel (cyOFP) (Figure 7). We operated the two LEDs in pulsed mode, interleaving them in time, while the two cameras recorded continuously as we encoded information about which LEDs were activated (Figure S4). Successful implementation of this protocol necessitated synchronizing the two cameras, operating in rolling shutter mode, with the pulsed light sources. To prevent spatially varying artifacts caused by uneven illumination of camera rows, the synchronization had to be precise down to the readout time (10 μs) of a single image row.

To implement the illumination interleaving scheme, we programmed the FPGA (40 MHz clock) to create trigger pulses for the LEDs, and we synchronized them to the global-exposure trigger from both cameras that marks the time period when all rows of the camera are simultaneously acquiring photons (Figure S4). Synchronization to the global exposure trigger allowed us to avoid spatial artifacts that might have arisen due to temporal mismatches between two cameras operating in rolling shutter mode. The FPGA created digital pulses that externally gated the LEDs. The FPGA also detected, counted, and, depending on the parity of the count number, rerouted global exposure signals to the LEDs (Figure S4). We used the triggering protocol shown in Figure S4E for dual-GEVI studies, in which exposure times were equalized across different rows of the camera yet remained longer than the nominal, fastest possible acquisition time given the number of rows being read out. For instance, the acquisition of 2048 rows at 100 fps led to negligible, 10 μs temporal overlap between the exposures of the first and last rows. An increase of the exposure time to 12.5 ms decreased the frame rate to 80 fps yet allowed us to use 2.5-ms-pulses of light that were precisely synchronized to the time period when all camera rows were exposed simultaneously.

The protocol outlined above was effective in cases when the fluorophores were expressed in a balanced manner, as shown in the results presented in Figure 7. However, when expression of cyOFP was stronger than that of the GEVIs, we adjusted the exposure time for the camera in the red fluorescence camera to reduce the amount of red fluorescence excited by the longer-duration blue pulse, which was also used to excite the green GEVI. To capture signals from 3 fluorophores with varying expression patterns and intensities, cameras with different exposure times are needed in principle. However, the use of multiple illumination sources can result in extra intensity fluctuations, and running two cameras in frame-by-frame trigger mode can introduce exposure and illumination jitters that degrade the accuracy of the data. Therefore, for studies with 2 GEVIs, we implemented a protocol using the External Edge Trigger mode and trigger signals generated by the FPGA clock, which allowed the two cameras to operate with different exposure durations.

#### Software for TEMPO imaging

We controlled the TEMPO mesoscope using custom software written in LabVIEW (2019) (National Instruments). The high-level architecture of our software has 3 main components (Figure S4F). The main software, Recorder, was the primary controller of most of the hardware components and served as the primary user interface. To create Recorder interfaces to the camera, LEDs and mechanical actuators, we respectively used a software development kit (SDK) from Hamamatsu, and serial port communication protocols from Prizmatix and Sutter. Recorder exchanges the software state and metadata with subsequent FPGA programs, split into Host and Target programs. The diagram in Figure S4F shows all software submodules and the relationships between them, as well as with the external devices, lines or file containers.

The submodules of Recorder, running on the PC, handle several functions. The camera setting manager interfaces with the sCMOS cameras, while the DCAM streamer interfaces with a set of proprietary DLL libraries from Hamamatsu. The software includes a file system manager and a metadata manager to store and organize data that we designed and implemented to support our experiments and data analysis pipeline. A shared setting server allows multiple users to access the same settings. Image frames from the cameras are buffered for real-time monitoring, and the software runs simple analytics to check data quality. The Recorder software also enables active control of the LEDs and focusing stage, which are synchronized to the recordings. The LEDs are turned on only during the recording and are pre-launched 0–10 s before recording starts, to remove the fastest photobleaching components from the fluorophore signals.

The FPGA host software includes a shared settings server that links camera and FPGA settings. A key feature of this software is its ability to take FIFO input from the FPGA target, which buffers and streams data from various sources, including VSYNC signals, behavioral stimulation signals, position and velocity data from the rotary encoder, behavioral properties, real-time averaged signals from LEDs matched to the frame duration, and time stamps with microsecond-precision. The VSYNC is a synchronization signal used in camera control that indicates the start of the image sensor’s exposure cycle.

The FPGA target software constitutes the actual code running on the FPGA. It has several modules that generate pulses to control the cameras and LEDs, edge detectors, counters and quadrature encoders. There is also a pulse selector for dual-GEVI studies and a real-time averaging module that provides a convenient readout of LED power, resampled to the frame-rate. The FPGA target software communicates with the host software via a FIFO, containing the data specified above.

#### Mouse preparation for mesoscope TEMPO measurements

Mice (aged 4–16 weeks at start) underwent two surgical procedures under isoflurane anesthesia (1.5%–2% in O_2_). The first procedure involved virus injection, to express the fluorescent proteins. In the second procedure, we implanted either a glass window (7–8 mm diameter) in the neocortex covering ∼20 brain regions (Figures 4, 5, 7, and S7) or a 3 mm-diameter optrode device in the hippocampus (Figures 6 and S7; see description above). For cortical imaging, we injected the PHP.eB virus retro-orbitally in ~5-week-old mice. For TEMPO imaging experiments of one cell type (Figures 4, 5, and S7), we injected 100 μL of a Ringer solution containing 1.5E11 GC of AAV2/PHP.eB-EF1α-DIO-ASAP3 virus and 1E11 GC of AAV2/PHP.B-CAG-mRuby2 virus. For imaging studies of two neuron-types (Figures 7A–7N), we injected 100 μL of Ringer solution containing 1.5E11 GC of AAV2/PHP.eB-EF1α-DIO-ASAP3 virus, 2.5E11 GC of AAV2/PHP.eB-CaMKII-Varnam2 virus, and 1E10 GC of AAV2/PHP.eB-CAG-cyOFP virus. For studies of two neuron-types with reversed GEVI assignments (Figures 7O and 7P) we injected 100 μL of Ringer solution containing 3E11 GC of AAV2lPHP.eB-CaMKII-ASAP3 virus, 1.5E11 GC of AAV2/PHP.eB-EF1a-DIO-Varnam2 virus, and 1E10 GC of AAV2/PHP. eB-CAG-cyOFP virus. For controls in which neither fluor was a GEVI (Figure S7A), we injected 100 μL of a Ringer solution containing 1.5E11 of AAV2/PHP.eB-CAG-GFP virus and 2E12 GC of AAV2/PHP.B-CAG-mRuby2 virus. For hippocampal imaging studies (Figure 6), we locally injected viruses into dorsal CA1 at coordinates relative to Bregma of (AP, ML, DV): −1.8, −2.5, −1.3. We injected 500 nL of a viral solution containing 5E9 GC of AAV2/PHP.eB-EF1α-DIO-ASAP3 virus and 1E9 GC of AAV2/PHP.B-CAG-Ruby3 virus. See above for a description of the implant for hippocampal imaging.

For neocortical studies, we implanted a glass window (7–8 mm diameter) on the right hemisphere of ∼12-week-old mice, ipsilateral to the retro-orbital injection. For 7-mm-diameter windows we used #64–0723 coverslips from Warner Instruments. To create larger windows, we manually cut them from #22–266-940 cover glasses from Fisher Scientific. The surgical procedure was similar to that of our previously published work.^130^ We anesthetized mice with isoflurane (4% for induction and 1%–2% during surgery) on a stereotaxic frame (Model 963, David Kopf instruments). To mitigate post-operative pain, we injected carprofen (5–10 mg/kg) subcutaneously prior to any surgical incision. We applied eye ointment to the mouse’s eyes to maintain their moisture. A heating pad (FHC, 40–90-8D) maintained the mouse’s body temperature at 37°C.

We removed the skin atop the cranium and mechanically removed all soft tissues from the skull surface with a scalpel. Next, we used a 0.7-mm-diameter micro drill burr (#19007–07, Fine Science Tools) to perform a craniotomy along AP 3.0 to AP −5.0 (referenced to the mouse Bregma), approximately matching the diameter of the implanted glass window size. During this most critical step, it was important to avoid drilling past the desired diameter so that the skull and window edges fit properly. Moreover, it was important to minimize damage to the dura to prevent excessive bleeding. We applied mammalian Ringer’s solution (#11763–10, Electron Microscopy Science) throughout the drilling to rinse off debris and minimize tissue heating. After completing the drilling, we disconnected the resulting circular-shaped bone piece from the surrounding skull. Once the bone piece was disconnected from the skull, we centered a plastic pipette tip atop the detached skull bone. We then replaced the bone piece with the glass coverslip and pressed the window ∼100 μm downward to flatten the cortical brain tissue beneath the window. We dried the edges of the window implant with surgical eye spears (#1556455, Butler Schein Animal Health) and glued the window to the skull with UV-light-cured adhesive (#4305, Loctite), while protecting the mouse’s eyes with aluminum foil to prevent UV-light damage. To allow head-fixation during brain imaging, we installed a stainless steel annular headplate (12 mm inner diameter), concentric with the glass window, and we filled the gap between the headplate and the skull with dental cement (#10000787, Fisher Scientific).

We transferred the mouse to a recovery cage and placed it on a heating pad until it awoke. We then returned the mouse to its home cage and provided food and water on the cage floor without use of a food hopper. We subcutaneously administered the mice carprofen (5 mg/kg) for the first 3 days after surgery to reduce post-surgical discomfort. For the first 7 days after surgery, we checked all mice daily for signs of distress. Mice recovered for at least 2 weeks before imaging experiments began.

#### Recording sessions for TEMPO microscopy

We performed all recordings during the mouse’s light cycle. Before every recording session, we first removed cage dust deposited onto the mouse cranial window using a cotton tip sprayed with ethanol and immediately blew away the excess ethanol using an air duster tank. Next, to align the mouse cranial window orthogonal to the microscope optical axis, we used two goniometers to control the mouse head-stage in two rotational angles (tilt and yaw). We used a ∼1 mW laser pointer (CPS532-C2, Thorlabs) that was directed vertically onto the window and aligned the reflection off the cranial window back to the laser output. For experiments using ketamine-xylazine anesthesia (Figures 4 and S6), recordings began ∼20 min after anesthesia administration, when mice became immobile, and recording durations were between 2–10 min. Imaging experiments involving visual simulation of head-restrained mice (Figures 5, 7, S6, and S7) were similar to those performed with fiber-optic TEMPO (Figures 3, S2, and S7), and the recordings lasted up to 10 min. Imaging experiments involving head-restrained locomotion (Figures 6 and S7) were similar to those performed with fiber-optic TEMPO (Figures S2 and S3), and recordings lasted up to 10 min. Recording depths were ∼200–300 μm for cortical imaging (Figures 4, 5, 7, S6, and S7) and ∼50–100 μm for hippocampal imaging studies (Figures 6 and S7). Across all experiments, we used up to 150 mW of blue LED and 110 mW of green LED illumination across the 7–8 mm-wide field-of-view.

#### Histology and fluorescence microscopy of brain slices

At the end of *in vivo* experimentation, we deeply anesthetized mice with isoflurane. We transcardially perfused the mice with phosphate buffered saline (PBS) (pH 7.4), followed by 4% paraformaldehyde (PFA) in PBS. We fixed brains in PFA at 4°C for at least 24 h and prepared tissue sections (∼100 μm) using a vibrating microtome (VT1000S, Leica). We washed the sections with PBS several times and incubated them in 150 mM glycine in PBS for 15 min to quench fluorescence induced by PFA. We then washed sections 3 times in PBS, and mounted sections with Fluoromount-G (Southern Biotech).

To capture the native fluorescence of the GEVIs (ASAP3, Ace-mNeon1, Varnam1 or Varnam2) and reference fluorescent proteins (GFP, mRuby2, mRuby3 or cyOFP), we used a commercial confocal system (Leica SP8) with a 0.75 NA, 20x immersion objective and hybrid photomultiplier tube. We used continuous wave laser excitation, of 488 nm or 561 nm wavelength, along with green fluorescence (525/50) and red fluorescence (600/50) filters (Figures 1, 3, 7, S2, and S6).

### QUANTIFICATION AND STATISTICAL ANALYSIS

#### Data processing for fiber-optic TEMPO recordings

For all fiber-optic TEMPO recordings (Figures 1, 2, 3, S1–S3, and S7), we analyzed the data using a custom-written uSMAART analysis package (https://github.com/sihaziza/uSMAART_public; https://doi.org/10.5281/zenodo.15679319) that runs in MATLAB (Mathworks). In brief, we first loaded the raw, unfiltered data into MATLAB. Next, we applied a notch bandstop filter to remove the artifacts induced by the dynamic diffuser from the decoherence stage (see above, instrumentation for fiber-optic TEMPO measurements). We designed this bandstop filter using the MATLAB function, *designfilt()*, and created an infinite impulse response, 8th-order Butterworth filter with center cut-off frequencies of 300 ± 1 Hz or 600 ± 1 Hz to remove the fundamental and its first harmonic, respectively. We applied this filter to the voltage and reference traces using the zero-phase-delay MATLAB implementation, *filtfilt()*.

To remove the photobleaching dynamics, we then detrended the fluorescence signals by normalizing the trace by the time-varying baseline fluorescence, $F_{0}$, as estimated using a temporal low-pass filter (4th-order Butterworth filter, 0.5 Hz cut-off frequency). As is common in fluorescence studies, photobleaching typically exhibited multi-exponential kinetics, which we characterized through parametric fits. For example, we found that the characteristic time interval, $t_{1/2}$, over which ASAP3 fluorescence decays to half of its initial value is $t_{1/2}=2.5$ h, using an illumination intensity of 2.8 mW/mm^2^. We calculated this value by fitting the time-course of ASAP3 photobleaching with a tri-exponential decay, with 3 time-constants (*t*_fast_ = 26 s; *t*_mid_ = 300 s; *t*_slow_ ≈ 5.5 h), which provided a substantially better fit than a bi-exponential function. For Varnam2 and mRuby2, we determined that $t_{1/2}$ is 6.4 h for an illumination intensity of 0.25 mW/mm^2^ by using a bi-exponential fit (*t*_fast_ = 291 s; *t*_slow_ ≈ 9.7 h). These $t_{1/2}$ values are much longer than those reported for voltage imaging studies at single action potential resolution (*e.g.*, $t_{1/2}<10$ min for ASAP3,^55^ Ace-mNeon2 and Varnam2^1^), since TEMPO uses ∼20–200 times weaker illumination intensity levels than imaging studies of neural spiking.

Finally, to remove the contributions of non-voltage dependent artifacts and noise sources to the fluorescence voltage trace, we unmixed the voltage and reference channel traces using our custom-designed convolutional unmixing algorithm (below).

In dual cell-type TEMPO studies (Figures 3, S2, S3, and S7), prior to the convolutional unmixing step, we included a decrosstalking step to correct for fluorescence crosstalk between the demodulated cyOFP and Varnam2 time traces. The rationale for this step is that fluorescence signals collected from cyOFP will, by design, include some crosstalk from the voltage-dependent Varnam2 indicator. Because the blue laser excites both of these fluors, ∼10.3% of the fluorescence collected from Varnam2 appears as crosstalk in the demodulated cyOFP signal, as calculated under the condition that the blue and green laser emissions are equal in intensity at the specimen. The demodulated cyOFP signal also includes some crosstalk from ASAP3 emissions, because ∼8.8% of the collected ASAP3 photons bleed into the red fluorescence detection channel. However, we observed empirically that crosstalk from Varnam2 was the dominant contaminant of the cyOFP signal, because (i) Varnam2 fluorescence was generally of greater amplitude than ASAP3 fluorescence under our fluorescence labeling conditions, and (ii) in real experimental situations, the blue laser was typically set at a ∼10-fold higher intensity level than that of the green laser (see values above) to compensate for the weaker expression of ASAP3.

To perform the decrosstalking, we regressed the demodulated cyOFP trace against the demodulated Varnam2 trace, using bandpass-filtered versions of both traces centered on the relevant frequency band (3rd-order Butterworth filter with cut-off frequencies set at 3–7 Hz for visual cortical studies and 5–9 Hz for hippocampal studies), which contains well-characterized voltage dynamics and is generally free of artifacts. We weighted the unfiltered Varnam2 trace by the value of the regression coefficient and subtracted the resultant from the unfiltered cyOFP trace, which was then used as one of the inputs to the convolutional filter. This decrosstalking step ensures that the subsequent convolutional filtering algorithm does not unmix or alter the voltage signals in the GEVI traces.

#### Frequency-dependent unmixing of voltage signals

We developed a custom algorithm for unmixing hemodynamics and other artifacts captured in the reference fluorescence channel from the voltage signals captured in the GEVI fluorescence channel (Figure S5). This convolutional filtering algorithm presumes that both artifacts and voltage signals have characteristic frequency domain representations and performs a frequency-dependent regression between the two channels, thus outperforming a frequency-independent regression.^131^ The frequency-dependent amplitudes and phases used for this regression constitute a convolutional filter. Although this appears to be the initial instance of convolutional filtering applied to voltage imaging, the concept of signal estimation through filtering was introduced decades ago by Kolmogorov^132^ and Wiener.^133^ Today, Wiener filtering is widely used in digital signal processing, control, image processing, and other fields of engineering.^80^

Here, we describe the algorithm for the case in which there is one GEVI. In studies with two GEVIs, we unmixed the reference channel content from each voltage channel individually. We treat the case in which one detection channel collects fluorescence from a GEVI (*e.g.*, ASAP3 or Varnam2) and the other collects emissions from a reference fluor (*e.g.*, mRuby2 or cyOFP) (Figure S5A). The data generally have 5 notable characteristics: (1) the GEVI channel contains a mixture of neuronal voltage-dependent and non-neuronal voltage-independent signals, whereas the reference channel only contains signals of the latter type, *i.e.*, that are independent of neural membrane potentials and thus are artifacts for the purposes of voltage imaging; (2) the biological artifacts (*e.g.*, hemodynamics and brain motion) and instrumentation noise (*e.g.*, illumination fluctuations) generally have intrinsic temporal frequency signatures; (3) oscillatory hemodynamic signals are generally the artifacts of the greatest magnitude and have characteristic frequencies concentrated around that of the heartbeat and its harmonics (Figure S5B); (4) hemodynamic oscillations are present in the same frequency bands and are generally coherent between the two channels, but do not always oscillate with the same phase in the two channels (Figure S5B); (5) the spatial distribution of hemodynamic artifacts is non-uniform across the field-of-view (see *e.g.*, Figure S5L).

We implemented our unmixing algorithm using the assumption that the signals in the GEVI channel, G(t), are a sum of the voltage signals, V(t), plus a voltage-independent component, H(t):

(Equation 1) G(t)=V(t)+H(t)

Based on the above observations (1–5), we formulated the problem of estimating the non-voltage signals, H(t), as a Wiener filter estimation problem in which we seek a filter, F(t) such that the convolution of the reference channel trace, R(t), with the filter, F(t), yields H(t),

(Equation 2) H(t)=(F∗R)(t)

where the ∗ operation denotes a convolution. Our physiological intuition for why this formulation makes sense is that H(t) predominantly arises from changes in the optical properties of the tissue, due to temporal variations in oxygenated (HbO) and deoxygenated (Hb) hemoglobin content. These two forms of hemoglobin will have distinct influences on the GEVI and reference fluorescence signals, but these influences are not independent. A simplified way of depicting the dynamics relating the concentrations of Hb and HbO is to represent them as a linear dynamical system, which implies a linear dynamical relationship between R(t) and H(t) that can be represented as a convolution in the time domain. With this framework, the main steps of our algorithm are (Figures S5C and S5D):

1. We split the traces from the GEVI, $G_{k}(t)$, and reference channels, $R_{k}(t)$, into N temporal segments of duration τ (typically 0.5–2 s) indexed by the variable k=0,1,…N, and with fractional overlap (1−γ)∈[0,1] (typically 0.75)
  (Equation 3) $$G_{k}(t)=G(t+k\gamma \tau ),R_{k}(t)=R(t+k\gamma \tau ),t\in [0,\tau )$$
  We compute the corresponding Fourier transforms $g_{k}(\omega )$ and $r_{k}(\omega )$:
  (Equation 4) $$g_{k}(\omega )=F\left\{G_{k}(t)w(t)\right\},r_{k}(\omega )=F\left\{R_{k}(t)w(t)\right\}$$
  where F{⋅} denotes a Fourier transform and w(t) is a windowing function (*e.g.*, a Hann window).
2. We estimate the Wiener filter in the frequency domain by concurrently fitting a complex coefficient, f(ω), for every frequency, ω, to approximate the GEVI channel signal $g_{k}(\omega )$ with the reference channel signal $r_{k}(\omega )$ for all N segments,
  (Equation 5) $$f(\omega )=\frac{{\langle g_{k}(\omega )r_{k}^{*}(\omega )\rangle }_{k}}{{\langle r_{k}(\omega )r_{k}^{*}(\omega )\rangle }_{k}}$$
  where $r^{⋆}$ is the complex conjugate of r and ${\langle \cdot \rangle }_{k}$ denotes averaging over the N segments. We limit the upper bound of the filter amplitude relative to the amplitude of the linear regression estimated at the heartbeat frequency $\omega_{0}$, with α>0 (typically 1.1, see Figure S5M)
  (Equation 6) $$f\left(\omega^{\prime }\right)=\alpha \left|f\left(\omega_{0}\right)\right|e^{i\cdot arg\left(f\left(\omega^{\prime }\right)\right)},\omega^{\prime }:\left|f\left(\omega^{\prime }\right)\right|>\alpha \left|f\left(\omega_{0}\right)\right|$$
  If $\omega_{0}$ is the fundamental harmonic of the heartbeat frequency, the amplitude $\left|f\left(\omega_{0}\right)\right|$ can be interpreted as a linear regression coefficient between the channels filtered at the heartbeat frequency. We set the spectral amplitude limit for the filter to $\alpha \left|f\left(\omega_{0}\right)\right|$, thus explicitly prohibiting unbounded amplification.
3. We apply an inverse Fourier transform to find the time-domain representation of the filter F(t)
  (Equation 7) $$F(t)=F^{-1}\{f(\omega )\}$$
  We convolve F(t) and R(t) in the time-domain to estimate the non-neuronal contribution H(t), and subtract H(t) from the GEVI signal G(t) to recover the unmixed voltage signal V(t):
  (Equation 8) V(t)=G(t)−H(t)

The above algorithm can be interpreted as a rank-constrained linear regression in the frequency domain. We fit a complex-valued scaling coefficient f(ω) for every frequency ω to approximate the voltage channel signal $g_{k}(\omega )$ with the reference channel signal $r_{k}(\omega )$ for all segments, k. The rank of the regression (*i.e.*, the number of free parameters or the number of points in the filter) is equivalent to the filter length, τ, and thus inversely proportional to the spectral resolution.

We determined the appropriate segment length τ (usually 1.0–1.5 s) and optimal relative spectral limit, α (1.0–1.3) by splitting the data in half into train and test segments and minimizing the remaining signal in the test movie segment after unmixing with respect to the parameters τ and α. The unmixing procedure and the results of the paper are robust to minor changes in the values of these parameters (Figure S5M). Larger segment lengths allow for greater spectral resolution of the filter, whereas smaller segment lengths result in more robust estimation in low SNR recordings.

In some recordings, the noise floor in the reference channel was higher than that in the GEVI channel (see *e.g.*, Figure S5H). For such recordings, unmixing would not be fully successful in removing signal artifacts from the voltage channel. Therefore, we performed a pre-filtering step that consisted of estimating $H^{avg}(t)$ using a spatially averaged reference movie $R^{avg}(t)$, and then removing $H^{avg}(t)$ at the single pixel level:

(Equation 9) $$V^{res}(t)=G(t)-H^{avg}(t)$$

Then, we performed a second convolutional filtering at the single pixel level between signals in the reference channel, R(t), and those in the voltage channel, $V^{res}(t)$. The pre-filtering step is reasonable because, as non-neuronal activity generally does not travel spatially, it boosts the SNR of $R^{avg}(t)$. The suitable scale of spatial averaging depends on the relative noise levels between the two channels and was usually set to be ∼0.5–1.5 mm. Although more spatial averaging leads to improved SNR values (*e.g.*, compare the single-pixel and spatially-averaged traces in Figures S5H and S5I), reduced spatial averaging allows the spatial variations of the hemodynamics to be captured with higher accuracy. Overall, compared to a simple (*i.e.*, frequency independent) regression,^131^ our unmixing method better removed broad- and narrow-band artifacts and did not transfer noise from the reference to the GEVI channel, which was critical for detecting high-frequency activity with high sensitivity (Figures S5E–S5J).

Software for convolutional filtering and unmixing is available at https://github.com/schnitzer-lab/CoReU and https://doi.org/10.5281/zenodo.15686223.

#### Processing of TEMPO mesoscope video data

We preprocessed the data using a custom pipeline written in MATLAB (https://github.com/schnitzer-lab/TEMPO-processing; https://doi.org/10.5281/zenodo.15686336). This pipeline comprised the following main steps:

- Loading and conversion: We first loaded the raw, 16-bit *.dcimg* video files from the GEVI and reference channels into the computer RAM in small batches (∼10% of the available RAM memory). We spatially downsampled each movie by applying 8 × 8 binning using the Matlab function *imresize3()*, with the ‘box’ interpolation method that performs averaging over all 64 pixels. We saved the 3D matrices as single floating-point numbers (32-bit) in HDF5 file format to a separate RAID0 SSD array. The HDF5 files also contained the recording settings (*e.g.*, imaging frame-rate).
- Registration: We next registered the GEVI and reference movies to correct for any static spatial displacements between them. We did this by estimating a rigid transformation, which corrects for translation and rotation, between representative image frames of spatially bandpass-filtered versions of the green and red fluorescence movies. The image from the green channel served as the reference because of its superior optical contrast showing the vasculature pattern. We estimated the affine transformation between the green and red images using 5 different approaches: (1) an intensity-based method (MATLAB function *imregcorr(*)), (2) a phase correlation-based method (MATLAB function *imregcorr(*)), and (3) a mutual information correlation-based method (MATLAB function *imregtform(*)). The last two approaches were implemented twice, using either the ‘mono-modal’ or ‘multi-modal’ option, yielding a total of 5 methods. The ‘mono-modal’ option assumes similar brightness and contrast between the two images, whereas the ‘multi-modal’ option does not.^134,135^ All 5 approaches performed differently for different movies. Therefore, we evaluated their respective performance using the structural similarity index measure (SSIM; MATLAB function *ssim(*)), computed between the fixed green image and transformed red image. We used the image registration approach that yielded the highest SSIM value and then applied the transformation obtained with it to all individual movie frames of the red channel.
- Motion correction: Both GEVI and reference channel movies underwent rigid motion-correction using phase correlation image registration^136^ within the NoRMCorre^137^ software package and were then cropped to remove parts of the images lacking fluorescently labeled brain tissue. We used the rigid registration mode of NoRMCorre, instead of non-rigid registration, as our image data primarily exhibited rigid displacements, and we found the former mode of operation to be more robust and faster than the latter. The NoRMCorre parameter shift_method was set to ‘cubic’, instead of the default ‘fft’.
- Decrosstalking: To remove any residual crosstalk between the GEVI and reference channels, we first estimated the fraction of fluorescence bleedthrough from the optical filters’ properties. In practice, ∼7% of ASAP3 (∼9.5% for AcemNeon1) fluorescence signals bled into the reference channel. We scaled the green fluorescence movie (usually the GEVI channel) by the estimated coefficient and subtracted the resultant from the red fluorescence movie (usually the reference channel). We did not correct for bleedthrough of the red fluor into the green fluorescence detection channel, since the fluorescence filters were expressly chosen to keep this value negligible (∼0.1% for bleedthrough of mRuby or Varnam into the green channel).
- Detrending: To account for fluorescence photobleaching, at each time point in the fluorescence video, F(t), we normalized each pixel by $F_{0}(t)$, the time-varying value of the pixel’s baseline fluorescence. The resultant is the detrended movie, $F(t)/F_{0}(t)$. For Figures 4, 5, 6, and S6, the detrending involved normalizing the trace by the time-varying baseline fluorescence, $F_{0}$, which we estimated using a first-order exponential fit (see typical values, in data processing for fiber-optic TEMPO recordings, above). We then applied a temporal high-pass filter to the data [type-1 FIR filter (symmetric, even order, linear phase; >10^5^ stopband attenuation, <10^–2^ passband ripple) designed using the *design()* function of the MATLAB DSP System Toolbox; 0.5 Hz cutoff frequency for anesthetized mice, 1.5 Hz for awake mice]. For Figure 7, the detrending involved normalizing by the time-varying baseline fluorescence $F_{0}$, as estimated using a temporal low-pass filter (4th-order Butterworth filter, 0.5 Hz cut-off frequency).
- Unmixing: To remove broad- and narrow-band hemodynamic artifacts as well as residual brain motion, we used the custom convolutional unmixing approach described above.

For dual cell-type recordings (Figure 7), we incorporated an additional step in which we upsampled the videos in the temporal domain by twofold and temporally shifted them by half of the nominal frame duration, *i.e.*, by 1 frame after interpolation. This allowed us to eliminate any phase shifts between signals obtained from cameras using interleaved light sources, thereby simplifying the unmixing of the fluorescence signals. As with dual cell-type uSMAART studies, in dual cell-type imaging studies we similarly included a decrosstalking step to remove Varnam2 signals that bled into the cyOFP detection channel. For studies of visual cortex, we computed mean cyOFP and Varnam2 traces that were each spatially averaged over primary visual cortex. We regressed the cyOFP trace against the Varnam2 trace, using bandpass-filtered versions (3rd-order Butterworth filter with a 3–7 Hz passband) of both traces centered on the 3–7 Hz range that contains the strong neural oscillation that is reliably evoked at visual stimulus offset and is generally free of artifacts. Using the resulting regression coefficient, we corrected each frame and pixel value of the cyOFP movie by subtracting the coefficient-weighted value of the corresponding pixel in the Varnam movie. The corrected version of the cyOFP movie was then used as one of the inputs to the convolutional filter. This decrosstalking step ensures that the convolutional filtering algorithm does not unmix or alter the voltage signals in the two GEVI movies.

#### Fast denoising of TEMPO movies

For display purposes only, the images of Figure 4O were smoothed with a Gaussian low-pass filter (156 μm FWHM). For the analyses of Figure 6, after applying the frequency-dependent convolutional unmixing algorithm (above), we improved the signal-to-noise ratios of the videos by applying a custom denoising algorithm based on a singular value decomposition (SVD) of the movie data. Because the runtime of SVD scales cubically with the size of the movie, we first broke the unmixed movie into temporal segments, each of 2500 frames in duration. We denoised each segment separately and then re-concatenated them to obtain a denoised version of the full movie.

To denoise each movie segment, we reshaped it into a two-dimensional matrix, $M\in {\mathbb{R}}^{p\times d}$, where *p* is the number of video frames in the segment (*i.e.*, 2500) and d is the total number of pixels in the field of view. Using SVD, we decomposed M as a product, M=U⋅C, where $U\in {\mathbb{R}}^{p\times k}$ is a set of *k* low-rank components, and $C\in {\mathbb{R}}^{k\times d}$ are weighting coefficients. The components U are assumed to be semi-unitary, without loss of generality, and were obtained by computing the SVD of M. The number, *k*, of low-rank components retained in U was five; we found that this choice removed the grainy appearance of noise in the videos of wave dynamics without altering the underlying depiction of wave propagation. We calculated the coefficients as $C=U^{T}\cdot M$. For each row of the coefficient matrix, after reshaping it back into a two-dimensional image, we denoised it with the MATLAB routine *denoiseImage()*. This routine calls the MATLAB function *denoisingNetwork()*, which uses a deep convolutional neural network for denoising.^138^ The resultant is a set of denoised coefficients, $\hat{C}$, from which we computed the denoised movie segment, $U\text{.}\hat{C}$. We then reshaped the movie segment back to its original dimensions. After denoising all the movie segments, we assembled the denoised version of the full movie and proceeded to the analyses of hippocampal waves.

#### Computations of coherence between optical and electrical traces

To assess the coherence between the optical and electrical traces (Figures 1, 2, 3, S1–S3, and S7), we applied a Fourier-based approach and used the MATLAB function *mscohere()* to compute the coherence magnitude between the two time series, and we used the MATLAB function *cpsd()* to compute the coherence phase between the two time series. We applied both functions to 1-s-long data segments, chosen such that temporally successive segments from the traces overlapped by 0.8 s. In imaging studies, we computed the coherence between the LFP and the TEMPO voltage signal at each pixel in the field of view (Figures 6E and 6F). The Fourier approach for assessing coherence levels has been commonly used in neurophysiology.^110,139–142^ However, we also checked on a subset of our data that this method yielded qualitatively similar results as a wavelet-based approach to assessing coherence. This alternative method relied on the MATLAB function *wcoherence()*, which provides a time-frequency representation of the coherence between a pair of non-stationary time series without using computational time-windows of fixed duration.

In Figures 1H and 1I, to identify the resting and running epochs of freely behaving mice for which we did not have behavioral video tracking, we used the LFP time series to isolate periods of high versus low theta power, which is a well-accepted proxy.^8,60,143,144^ We validated this data segmentation approach on mice in which we had both the LFP and behavioral video tracking.

#### Behavioral video tracking and pose estimation for freely moving mice

To track the mouse’s two-dimensional (*x-y*) position in the raw.avi behavioral movies (Figures 1 and 3), we used the deep learning-based animal tracking algorithm, DeepLabCut (version 2.2.1).^145^ We labeled the mouse’s head and the base of its tail in 187 image frames that were randomly chosen from 5 video recordings. We split this dataset into portions used for training and testing in a 95/5 ratio. We used a ResNet-50-based neural network^146,147^ and 500,000 training iterations with default parameters. We used a p-cutoff of 0.6 to condition the *x-y* coordinates, which led to a training error of 1.24 pixels and a test error of 3.46 pixels (image size was ∼300 × 300 pixels). We then used this network to analyze behavioral videos taken under similar conditions. Because the estimated position of the base of the mouse’s tail was more accurate than the estimated head position, we used the displacements of the tail base across image frames as the basis for computing the mouse’s velocity.

#### Characterizations of cross-frequency coupling

To compute cross-frequency coupling between two oscillations of different frequencies (Figures 2 and S3), we first bandpass filtered the raw signal at the low-frequency bandwidth of interest. From this filtered version of the trace, we estimated the analytic signal using the Hilbert transform (MATLAB function, *Hilbert(*)) and computed the phase using the function *angle()*. Next, we estimated the wavelet spectrogram of the raw signal using the MATLAB function *cwt()* and computed the average across the high-frequency bandwidth of interest. Finally, we identified every phase reset of the low-frequency oscillation and aligned the high-frequency signal accordingly.

In studies of cross-frequency coupling (Figures 2C, 2D, 2G–2J, and S3J–S3L), the convention used for the phase of the carrier oscillations (delta or theta) was that zero degrees refers to the trough of the carrier oscillation, *i.e.*, the greatest hyperpolarization in the TEMPO signal (Figures 2C, 2D, and S3J–S3L) or the greatest hyperpolarization in the extracellular electric field recording (Figures 2G–2J).

To detect periods of epileptic spikes (Figure 2M), we high-pass-filtered the raw LFP signal (3 dB cutoff of 25 Hz; 6th-order Butter-worth) and detected spikes using the MATLAB function *findpeaks()*, with the input arguments *minpeakwidth*, *maxpeakwidth*, and *minpeakprominence* set at 2 ms, 20 ms, and 1000 μV, respectively.

#### Continuous and event-related spectrograms

To compute continuous spectrograms (Figure 3), we applied the MATLAB function *spectrogram()* to the LFP trace. To compute event-related spectrograms (Figures 3, 5, 6, 7, S1, S2, and S6), we first computed the wavelet spectrogram of the entire time-series using the MATLAB function *cwt()*. Then, we isolated the time points of each stimulus presentation and determined the mean spectrogram, averaged across the set of all stimulus presentations.

#### Identification of hippocampal layers using electrophysiological landmarks

To identify the boundaries between the different layers of CA1 hippocampus in our 32-site silicon probe LFP recordings, we examined the voltage traces recorded from each site during intervals in which there were either sharp-wave ripple (SWR) events or theta oscillations (Figures S3A and S3B). For each of the 32 sites, we arranged plots of the LFP traces in order of the depth into CA1 tissue, temporally bandpass filtered (3rd-order Butterworth) across either the ripple (120–180 Hz) or theta (5–9 Hz) frequency range. We identified the 4 canonical hippocampal layers using 3 electrophysiological landmarks: (1) the center of *stratum pyramidale* corresponds to the depth at which ripple oscillations have the greatest amplitude; (2) the anatomical boundaries of *stratum pyramidale* were estimated based on the tissue depths at which ripple power approximately vanished; (3) the anatomical boundary between *stratum radiatum* and *stratum lacunosum moleculare* corresponds to the depth at which theta oscillations change in both amplitude and phase.

#### Detection of hippocampal ripples from silicon probe recordings

To study ripple events (Figures 3Q and S3F), we applied the following detection algorithm using the data acquired on the recording site of our 32-site probe with the greatest ripple amplitude (Figure S3A). Specifically, to detect ripple events, we used a previously described method that involves bandpass-filtering the raw LFP signals at the ripple-band frequency (120–200 Hz; 3rd-order Butterworth filter) and then calculating the normalized squared signal (NSS).^148–150^ To compute the NSS, we first squared the filtered signal, then applied a moving maximum (MATLAB function *movmax(*)) with a 20-ms-window, and then performed a smoothing using a 20-ms-window (MATLAB function *smooth(*)). We scaled the resulting trace between 0 and 1 using the *rescale()* MATLAB function. To isolate ripple events, we then identified local maxima using the MATLAB function *findpeaks()* with the following option parameters, ‘MinPeakProminence’, ‘MinPeakDistance’, ‘MinPeakWidth’ and ‘MaxPeakWidth’ set to, respectively, 10%, 10 ms, 10 ms and 200 ms. We chose these values based on characteristics of hippocampal ripples in the published literature.^148–150^ All extracted ripple events were isolated within a 300-ms-window. Finally, we visually curated all automatically identified ripple events so as to exclude non-stereotypical ripples (*e.g.*, events corrupted by high-frequency noise or events that were recorded on all 32 recording channels, instead of being localized to the hippocampal pyramidal layer).

#### Seed-pixel spatial correlation maps

To characterize wave propagation, we selected a seed pixel located in the brain region of interest and computed the cross-correlation between the voltage signal at the seed pixel and every other pixel. We used the MATLAB function *xcorr()* to compute the cross-correlation map, $X_{i,j}(a,b)$, where i and j denote spatial indices and the pair, (*a, b*), denotes the temporal indices of a pair of video frames. In this way, we obtained cross-correlation and delay maps characterizing the spatiotemporal propagation of the wave present in the seed pixel.

#### Calculations of velocity distributions in visual cortex

To characterize traveling waves in Figures 4 and 5, we used the cross-correlation function to determine the level of similarity between pairs of time traces and the delay between them in a spatially resolved manner. For each pair of points in space, corresponding to pixels i and j, we computed the time-dependent correlation coefficient, $X_{i,j}(\tau )=C_{i,j}(\tau )/\sqrt{C_{i,i}(\tau )C_{j,j}(\tau )}$, where $C_{i,j}(\tau )=\int V_{i}(t)V_{j}(t-\tau )dt$. We then estimated the speed, $v_{i,j}$, at which a wave traveled between two points, $\left(x_{i},y_{i}\right)$ and $\left(x_{j},y_{j}\right)$, using $\tau_{max}=argmax_{\tau }\left\{X_{i,j}\right\}$ and $v_{i,j}=\sqrt{{\left(x_{i}-x_{j}\right)}^{2}+{\left(y_{i}-y_{j}\right)}^{2}}/\tau_{max,i,j}$. We applied a threshold $X_{i,j}>X_{min}$ to restrict speed calculations to pairs of points at which the voltage waveforms were similar. We chose $X_{min}$ values between 0.6–0.9, balancing the spatial range of the wave and the signal-to-noise ratio of the spatially resolved waveforms. To estimate wavefront gradients, we parametrically fit the spatial map of wavefront delays, τ(x,y), using least-squares fitting to the equation τ(x,y)=Ax+By+C. We then used the fit coefficients to calculate the local wavefront velocities, $\vec{v}=\left[A^{-1},B^{-1}\right]$. To improve the quality of the fit, we often performed additional spatial binning. We obtained the flow maps of Figures 4 and 5 by normalizing all velocity vectors to a uniform length. We computed the distributions of wave speed and traveling direction based on the velocity vector distributions.

#### Calculations of velocity distributions in hippocampus

To characterize the speeds and directions of traveling voltage waves in Figure 6, we performed these four steps: (1) Wave detection; (2) Space-time projection; (3) Wave crest identification; (4) Linear regression. We began these analyses using movie data that had already undergone convolutional unmixing (see Figure S5 and above section, “frequency-dependent unmixing of voltage signals”) and denoising (see “fast denoising of TEMPO movies”).

To detect wave occurrences, we (1) created a trace representing the time-dependent, net fluorescence averaged across the entire field of view; we identified wave events as intervals in which the net fluorescence exceeded 3 s.d. of the mean fluorescence signal (time-averaged across the entire trace). Next, we (2) projected the movie onto two orthogonal spatial vectors, x and y, yielding a pair of one-dimensional movies (see *e.g.*, Figure 6G). Then, for each wave event, we (3) determined the time bin at which each pixel in the pair of one-dimensional movies attained its maximum fluorescence signal. Finally, using robust linear regression with the MATLAB function *robustfit()*, we regressed the spatial location of each pixel in the one-dimensional movies against the time at which the pixel achieved its signal maximum (*i.e.*, with the spatial location as the independent variable). These regressions yielded a pair of wavenumber values, $\alpha_{x}$ and $\alpha_{y}$, with physical units of s/mm. We determined the wave’s vector speed and direction by converting the vector wavenumber from a Cartesian to a polar coordinate representation. (To avoid infinite values for estimated speeds, we ensured that both Cartesian wavenumber values were significantly different from zero (*p*-value <0.01, as determined using the above robust regression).

To validate this method of speed determination, we applied it to simulated movies of waves with known characteristics; we assessed the accuracy of velocity determination across a range of speeds, propagation directions, and signal-to-noise ratios (SNRs). These tests provided confidence in the wave velocities estimated for empirical datasets (Figure 6). Overall, in comparison to the method of velocity determination described in the preceding methods section, “Calculations of velocity distributions in visual cortex,” the 4-step method described here was faster and more robust when applied to low SNR movies, but was also unsuitable for waves that did not travel in a unidirectional manner.

#### Model to estimate the physiological time delay between two cell-types

During our joint studies of two neuron-types at once, we found that, owing to the voltage-dependence of voltage-indicator activation and deactivation time-constants, the different voltage-indicators used here (Ace2, pAce, and Varnam2) report oscillatory dynamics with slightly different phase delays in different neuron-types. In other words, the oscillatory phase-shift induced by temporal low-pass filtering varies slightly between the different indicators and neuron-types. Hence, to estimate the actual physiological time delay between the visually-evoked 3–7 Hz oscillations of cortical PV interneurons and pyramidal cells in Figures 7N and 7P, we conducted a series of 7 ancillary experiments to characterize the phase shifts arising from indicator kinetics (Figures S7D–S7F).

Each ancillary experiment involved a different pair of voltage indicator assignments to cortical PV and pyramidal cells and yielded an apparent time delay between the visually-evoked 3–7 Hz oscillations in the two neuron-types. We computed these time delays using the MATLAB cross-correlation function *xcorr()*, by applying it to every bout of oscillations measured in the green and red fluorescence channels (bandpass filtered between 3–7 Hz). The time at which the value of the cross-correlation function was maximized corresponds to the apparent time delay for a specific bout of 3–7 Hz activity, as studied with a specific pair of voltage indicators. For each mouse used to study a given indicator pair, we evoked 100 trials of oscillatory activity.

We then performed a $L_{1}$-regularized linear regression to determine the values of the time delays induced by indicator kinetics for the 6 different assignments of our 3 indicators to the 2 neuron-types, as well as the actual time delay between the oscillations of PV and pyramidal cells. To do this, we sampled the apparent time delays for 100 different oscillatory bouts, for each of the 7 experiments. Using these 700 measurements, we computed mean and s.d. values for each of the 7 different time delays. The loss function for the regression was the sum, over all 7 experiments, of the squared difference between the mean measured time delay and the predicted value based on the estimate for the actual delay plus the difference of the two estimated indicator-induced delays for each experiment, with each term in the sum weighted inversely by the square of the experiment’s computed s.d. value. The regularization term was the Euclidean norm of the 6-dimensional vector comprised of all 6 indicator-induced delays. Fitting results were not sensitive to the value of the $L_{1}$-regularization parameter, which we set to 0.01. Using this approach, and the constraint that indicator-induced delays must have positive values, we fit the regression model for 100 different samplings of measured time delays; from the results, we computed mean and s.d. values for each of the 7 different delay times across the 100 different samplings, as shown in Figure S7E.

To generate Figures 7N and 7P showing the distributions of estimated physiological time delays between PV interneurons and pyramidal excitatory neurons, we applied the linear regression model described above to the data taken in individual mice plus that from the 6 experiments in Figures S7D and S7E with distinct GEVI labeling assignments. In other words, for each imaging session in Figures 7N and 7P we determined the histogram of estimated physiological time delays through parametric fits to the measured values of the time-delays observed in each individual mouse, plus its 6 complementary experiments. We repeated the fitting procedure for each of the 4 recordings in Figures 7N and 7P.

#### Statistics

We performed statistical tests (all two-tailed) in MATLAB. For two-sample comparisons of a single variable, we used non-parametric Wilcoxon rank-sum or signed-rank tests [*ranksum()* and *signrank()*].

## Supplementary Material

MMC3

MMC1

MMC2

1

Supplemental information can be found online at https://doi.org/10.1016/j.cell.2025.06.028.

## Figures and Tables

**Table:** KEY RESOURCES TABLE

REAGENT or RESOURCE	SOURCE	IDENTIFIER

Bacterial and virus strains		

All AAV viruses	HHMI Janelia Viral Tools	N/A

Chemicals, peptides, and recombinant proteins		

ketamine	VEDCO	50989–996-06
xylazine	AnaSed	59399–110-20
tamoxifen	Sigma	T5648

Deposited data		

data repository	This paper	https://doi.org/10.25740/cj061qr5050

Experimental models: Organisms/strains		

PV-IRES-Cre	Jackson Laboratory	Jax #008069
Cux2-CreERT2	Allen Institute	MMRRC stock #032779-MU131
Rbp4-Cre KL100	Allen Institute	MMRRC stock #037128-UCD
FVB-Tg(Nr5a1-cre)2Lowl/J	Allen Institute	Jax #006364
Sst-IRES-Cre	Jackson Laboratory	Jax #013044
C57BL/6	Jackson Laboratory	Jax #000664
Ai218	Allen Institute	Jax #037940

Recombinant DNA		

pAAV-CaMKII-Ace-mNeon2	Addgene	#195526
pAAV-CaMKII-Varnam2	Addgene	#195527
pAAV-EF1α-DIO-ASAP3-WPRE	Addgene	#132318
pAAV-CaMKII-ASAP3-WPRE	This paper	#240052
pAAV-CaMKII-Ace-mNeon1-WPRE	This paper	#240053
pAAV-EF1α-DIO-Ace-mNeon2-WPRE	This paper	#240056
pAAV-EF1α-DIO-Varnam1-WPRE	This paper	#240054
pAAV-EF1α-DIO-Varnam2-WPRE	This paper	#240055
pAAV-CAG-cyOFP-WPRE	This paper	#240050
pAAV-CAG-mRuby2-WPRE	This paper	#240051

Software and algorithms		

MATLAB	Mathworks	mathworks.com
mesoscope TEMPO preprocessing (Zenodo)	This paper	https://doi.org/10.5281/zenodo.15686336
mesoscope TEMPO preprocessing (Github)	This paper	https://github.com/schnitzer-lab/TEMPO-processing (commit ba0f7b2)
fiber-optic TEMPO preprocessing (Zenodo)	This paper	https://doi.org/10.5281/zenodo.15679319
fiber-optic TEMPO preprocessing (Github)	This paper	https://github.com/sihaziza/uSMAART_public (commit e1876bc)
convolutional unmixing algorithm (Zenodo)	This paper	https://doi.org/10.5281/zenodo.15686223
convolutional unmixing algorithm (Github)	This paper	https://github.com/schnitzer-lab/CoReU (commit 3eeb4f6)

## Highlights

- Fiber photometry tracks high-frequency neural oscillations in freely moving animals
- Imaging large-scale traveling neural voltage waves in head-restrained behaving animals
- Coupled oscillations of distinct frequencies in specific neuron types of active mice
- Locomotion-evoked hippocampal theta and beta rhythms are bidirectional traveling waves
