# Imaging high-frequency voltage dynamics in multiple neuron classes of behaving mammals

**DOI:** 10.1016/j.cell.2025.08.010

**Published:** 2025-08-21

**Authors:** Simon Haziza, Radosław Chrapkiewicz, Yanping Zhang, Vasily Kruzhilin, Jane Li, Jizhou Li, Geoffroy Delamare, Rachel Swanson, György Buzsáki, Madhuvanthi Kannan, Ganesh Vasan, Michael Z. Lin, Hongkui Zeng, Tanya L. Daigle, Mark J. Schnitzer

In the original version of this article, two instances of “20–60 Hz” were inadvertently noted as “30–70 Hz.” The legend for Figure 3F and citation of this figure panel in the main text should refer to the gamma frequency range as 20–60 Hz, matching the range stated in the figure. The original article has now been corrected. The authors apologize for any confusion this may have caused.

