# Supplementary material for: Imaging high-frequency voltage dynamics in multiple neuron classes of behaving mammals
Source: Cell. Author manuscript; Available in PMC 2025 Nov 14. (PMC12616578; doi:10.1016/j.cell.2025.06.028)
Supplement: 1 [file NIHMS2097661-supplement-1.pdf]

CellPress
OPEN ACCESS

Supplemental figures

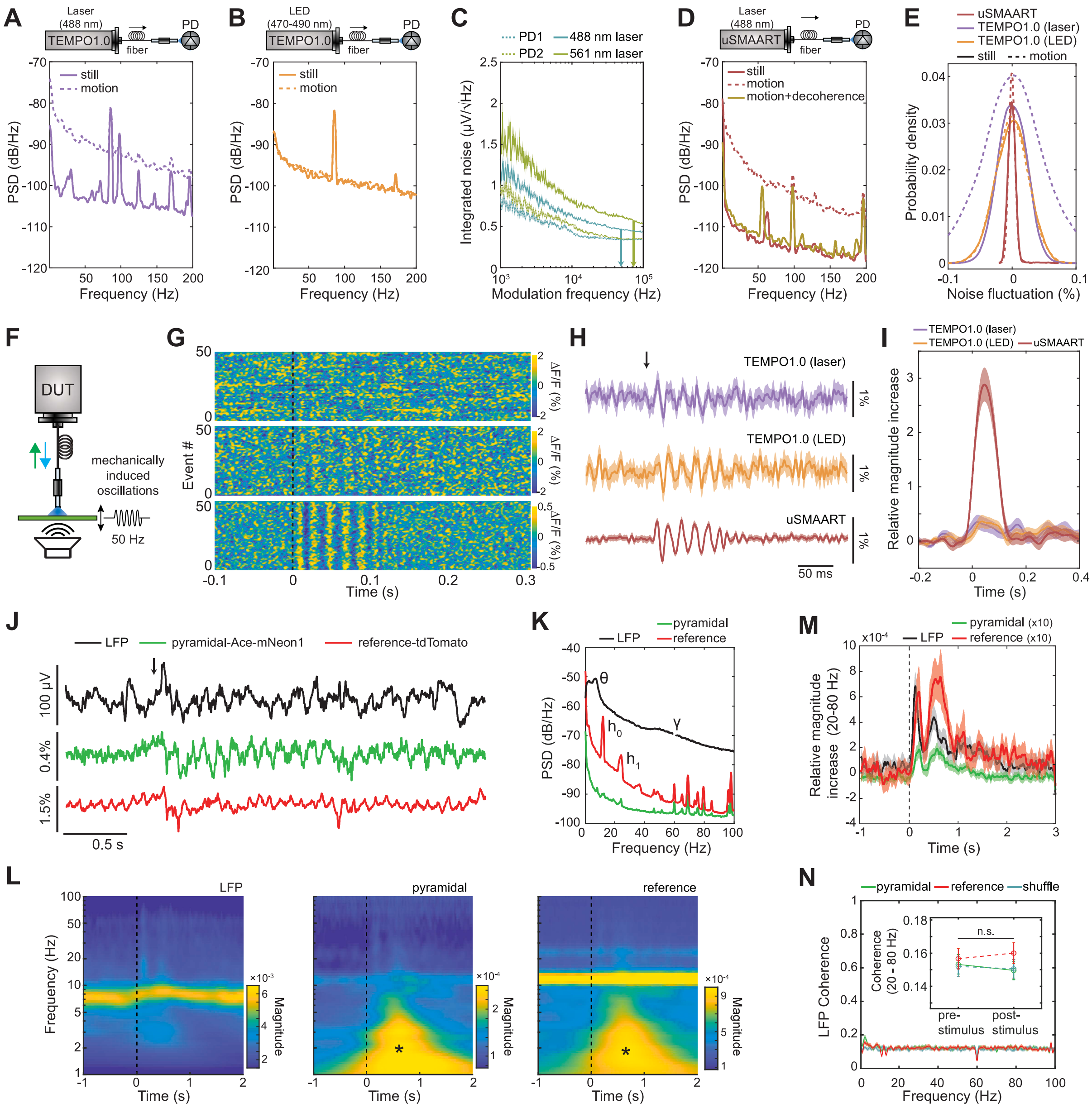

O

GEVI (*negative polarity*)	Subthreshold (% per mV)	Suprathreshold (% per mV)	Ratio sub/supra
ASAP3[a]	-1.06	-0.27	3.93
ASAP2[a]	-0.71	-0.27	2.63
Ace2[b]	-0.26	-0.26	1
Voltron[c]	-0.20	-0.20	1
Vnm2[b]	-0.19	-0.19	1
Ace1[d]	-0.12	-0.12	1
Vnm1[d]	-0.11	-0.11	1

GEVI (*positive polarity*)	Subthreshold (% per mV)	Suprathreshold (% per mV)	Ratio sub/supra
ASAP4e[e]	0.40	1.90	0.21
ASAP4b[e]	0.62	1.67	0.37
QuasAr6a[f]	0.73	0.73	1
Archon1[f]	0.70	0.70	1
pAce[b]	0.31	0.31	1
pAceR[b]	0.28	0.28	1
QuasAr6b[f]	0.24	0.24	1
Positron[g]	0.20	0.20	1

(legend on next page)

Figure S1. Benchmarking of uSMAART performance and tabulations of GEVI kinetics, related to Figure 1

(A–E) Coherent illumination from a solid-state laser had lower intensity noise fluctuations than the incoherent illumination of a LED but led to motion-induced optical mode-hopping within the multi-mode optical fiber used for photometry. Passing the laser light through a decoherence module before coupling it into the optical fiber made the light intensity at the fiber output insensitive to movement of the fiber.

(A) Power spectrum of illumination intensity noise as measured for a 488-nm laser (Obis; Coherent Inc.) that is commonly used for photometry, at the output of the multi-mode optical fiber, with (dashed curve) and without (solid curve) continuous shaking of the fiber. To perform a lock-in measurement, we sinusoidally modulated the laser output intensity at 3.5 kHz and used a digital lock-in amplifier to demodulate the signals from the photodetector to produce the plots shown (3.5 kHz demodulation frequency; 1 ms time constant). Mean illumination intensities at the output of the optical fiber were uniformly set to 50 μW in (A), (B), (D), and (E).

(B) Power spectrum of illumination intensity noise as measured for a commonly used LED (Thorlabs; ~470–490 nm spectral bandwidth), at the output of the multi-mode optical fiber, with (dashed curve) or without (solid curve) continuous shaking of the fiber. Although shaking the fiber did not induce additional intensity fluctuations, the baseline intensity noise was substantially higher than that of the 488-nm laser (compare with A). Illumination intensity modulation and signal demodulation were performed as in (A).

(C) We compared the levels of noise power in our photodetector signals when the laser sources were either turned off (dashed curves) or on (solid curves) as a function of the modulation frequency used for the lock-in measurements. Notably, higher modulation frequencies led to reduced noise fluctuations. Thus, for all subsequent fiber-optic measurements, we used modulation frequencies of either 50 or 75 kHz (marked with arrows on the graph).

(D) Power spectra of illumination intensity noise as measured at the output of the optical fiber for the 488-nm laser source and using a modulation frequency of 50 kHz without fiber movement (red solid curve), with continuous shaking of the fiber (red dashed curve), or with continuous shaking of the optical fiber plus the use of an optical decoherence module to prevent motion-induced mode-hopping (beige solid curve). Use of the decoherence module made the noise spectrum insensitive to fiber movement. Moreover, use of the 50 kHz modulation frequency reduced noise power levels by about 10 dB across the entire measurement bandwidth (0–200 Hz), in comparison with when the modulation frequency was 3.5 kHz (A).

(E) Probability distributions of illumination intensity noise amplitudes for the optical configurations used in (A), (B), and (D).

(F–I) Sensitivity benchmarking with a fluorescent slide confirmed the enhanced ability to detect gamma-band (50 Hz) signals using uSMAART.

(F) Schematic of an apparatus to generate artificial gamma-band oscillations. We affixed a fluorescent slide to the membrane of a voice coil actuator and drove the voice coil with a 50 Hz sinusoidal voltage wave (100 ms in duration). We positioned the tip of the multi-mode optical fiber from the photometry system (either TEMPO 1.0 or uSMAART) nearby the slide such that it detected ~150 pW of fluorescence photons, which is typical of *in vivo* TEMPO recordings from sparse neuron types using 50 μW of illumination power.

(G) Color raster plots showing relative changes in fluorescence evoked by the 50-Hz wave and captured by either a TEMPO 1.0 system using a laser source (top plot), a TEMPO 1.0 system using an LED source (middle plot), or the uSMAART system of Figure 1A (bottom plot). Data from 50 different trials are shown in each plot, unfiltered. Dashed vertical line marks the onset of the 100-ms-duration sinusoidal (50 Hz) wave.

(H) Traces of the mean time-dependent fluorescence during the 50-Hz waves averaged over 50 wave presentations for each of the 3 different fiber photometry systems. Shading: SEM.

(I) Mean time-dependent signal amplitudes in the gamma band (45–55 Hz) determined via a wavelet transform of the traces of (H). uSMAART reported the artificial gamma bursts with ~10-fold greater signal-to-noise ratios (SNRs) than prior TEMPO 1.0 instruments (gamma signal increases for uSMAART vs. TEMPO 1.0 [laser]: 2.9 ± 0.3 vs. 0.3 ± 0.1, $p < 10^{-11}$; uSMAART vs. TEMPO 1.0 [LED]: 2.9 ± 0.3 vs. 0.3 ± 0.1, $p < 10^{-11}$, rank-sum test). Shading: SEM over the same 50 trials as in (H).

(J–N) Re-analysis of stimulus-evoked fiber-optic TEMPO 1.0 dataset from Marshall et al.[7]

(J) Example set of concurrently acquired LFP, voltage, and reference fluorescence traces taken in a head-fixed mouse with our prior fiber-optic TEMPO 1.0 system.[7] The voltage indicator, Ace-mNeon1, was virally expressed in somatosensory cortical pyramidal cells via the *CaMKIIa* promoter, and the reference fluor was red fluorescent tdTomato. Black arrow marks the approximate start of vigorous whisker stimulation. The sustained post-stimulus oscillation at ~4 Hz could be due to motion-induced mode-hopping noise in the optical fiber, although similar features are also visible in the LFP trace.

(K) Power spectral density of all three signals in (J). The reference channel contains the heartbeat rhythm (h_0) and its harmonics (e.g., h_1), as well as high-frequency instrument noise in the 40–100 Hz range, some of which is also apparent in the GEVI channel.

(L) Wavelet spectrograms for all three signals in (J) and (K) averaged over 246 stimulus presentations from 3 mice ($t = 0$ is the time of stimulus onset). A theta-band oscillation is evident in the LFP recording (5–10 Hz), and the heartbeat (10–15 Hz) appears in the reference channel. Asterisks mark the onset of instrument noise at ~1–5 Hz after stimulus onset in both the pyramidal cell and reference channels.

(M) Mean signal magnitudes in the gamma range for all 3 signals shown in (L) plotted relative to baseline values and as a function of time relative to stimulus onset. All three traces have similar time courses, but as shown in (N), the GEVI signal increase is not phase coherent with the LFP increase. Shading in (M) and (N): SEM across $n = 246$ trials from 3 mice.

(N) Plots of the mean coherence between the LFP trace and either the reference, pyramidal cell, or temporally shuffled pyramidal signals, showing the lack of coherence between LFP and pyramidal voltage signals across the full 0.5–100 Hz frequency range. Inset: gamma coherence between LFP and pyramidal cell voltage during pre- and post-stimulus presentation periods (LFP/pyramidal: $p = 0.78$, LFP/shuffled pyramidal: $p = 0.68$; LFP/reference: $p = 0.93$; mean ± SEM, $n = 246$ trials from $n = 3$ mice).

(O) Fluorescence change per millivolt of membrane voltage change for negative (left) and positive (right) polarity GEVIs as estimated for the subthreshold (−100 to −40 mV) and suprathreshold (−40 to +30 mV) voltage ranges. Superscripts denote values that were extracted from the following publications: (a) Villette et al.[55]; (b) Kannan et al.[1]; (c) Abdelfattah et al.[3]; (d) Kannan et al.[8]; (e) Evans et al.[151]; (f) Tian et al.[152]; (g) Abdelfattah et al.[153]

CellPress
OPEN ACCESS

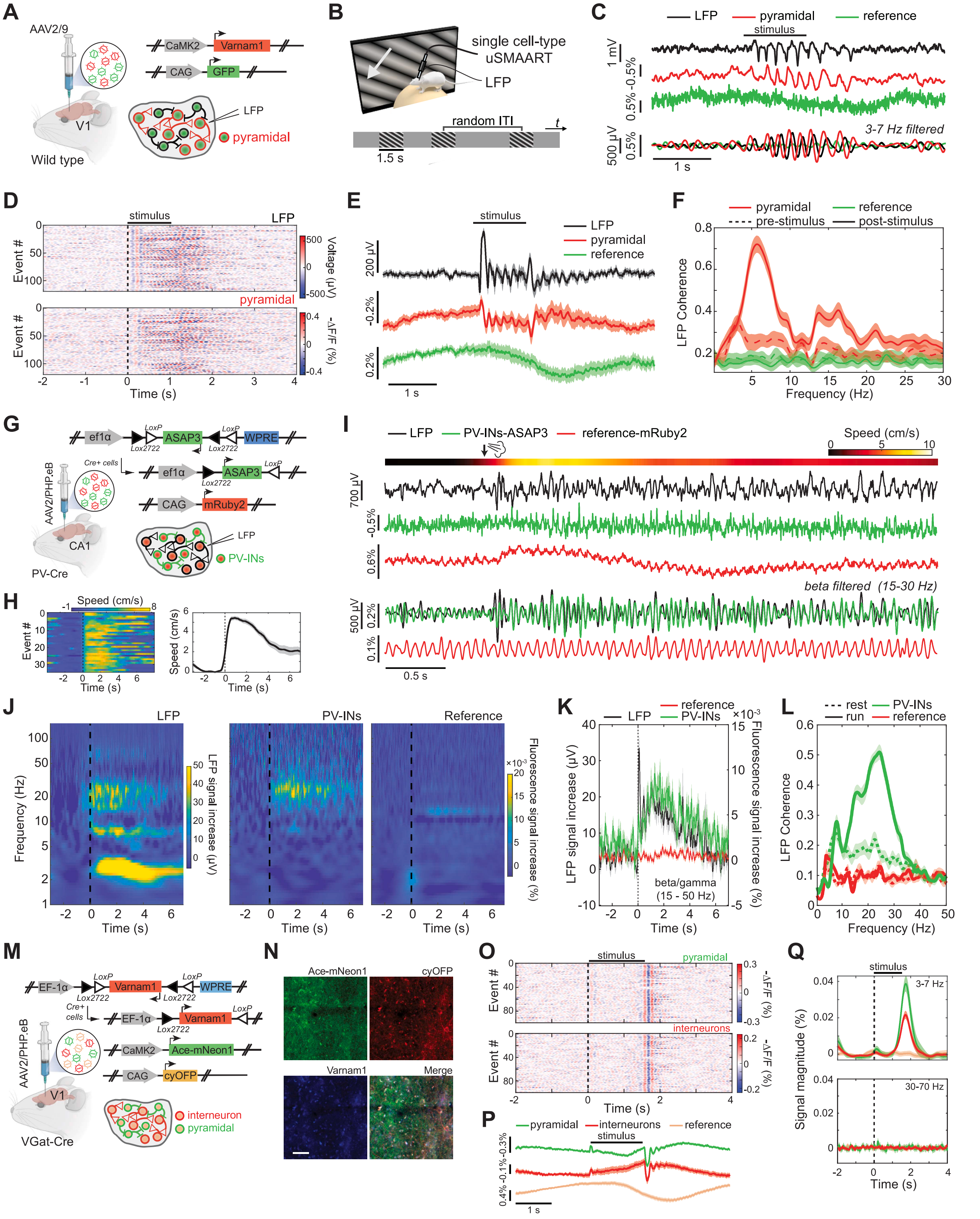

(legend on next page)

Figure S2. uSMAART captures high-frequency voltage dynamics in the primary visual cortex and hippocampus of head-fixed awake mice, related to Figure 1

(A–F) uSMAART studies of visually evoked high-frequency voltage dynamics in pyramidal neurons in the primary visual cortex of awake mice.
(A) Genetic constructs used to virally express the voltage indicator Varnam1 in glutamatergic neurons and the reference fluor GFP in a variety of neuronal cells.
(B) We provided visual stimuli using the same protocols as in Figure 3B.
(C) Example time traces of LFP, pyramidal cell membrane voltage, and reference fluorescence signals during visual stimulation along with bandpass-filtered (3–7 Hz) versions of the same traces. The vertical scale bar for the bandpass-filtered fluorescence traces indicates the percentage magnitudes but not the signs of the fluorescence changes in both the red and green traces, which are plotted with the same sign conventions as those of the color-corresponding unfiltered traces.
(D) Raster plots of LFP (top) and pyramidal cell voltage signals (bottom) showing that visual stimuli consistently evoked 3–7 Hz oscillations. Each row shows data from one of 115 different visual stimulation trials in the same mouse of (C).
(E) Mean time-dependent fluorescence traces obtained by averaging each of the 3 signals across all 115 trials of (D). Shaded area: 95% CI.
(F) Plots of the coherences between the LFP and either pyramidal cell (red curves) or reference fluor (green curves) signals during 2-s intervals immediately before the onset (dashed curves) or right after the offset (solid curves) of visual stimulation. Pyramidal cell voltage but not reference signals displayed visually evoked increases in coherence with the LFP up to 30 Hz ($p < 0.05$, rank-sum test). Shaded area: 95% CI across all 115 trials of (D).
(G–L) uSMAART studies of how rest-to-run transitions impact beta- and gamma-band activity in hippocampal PV interneurons. We delivered airpuffs to the mouse's back to induce running.
(G) Genetic constructs used to virally express the voltage indicator ASAP3 in PV interneurons of hippocampal area CA1 along with the reference fluor mRuby2.
(H) Left: raster plot showing time courses of locomotor speed by a head-fixed mouse on a running wheel. Right: plot of the mean time-dependent locomotor speed averaged over 36 rest-to-run transitions. Vertical dashed lines mark the time of airpuff delivery. Analyses of this animal are shown in (I)–(L).
(I) Top: color plot showing the change in locomotor speed induced by an individual airpuff (black arrow). Bottom: time traces of concurrently recorded LFP and fluorescence signals. The top 3 traces are unfiltered, whereas the bottom three are bandpass filtered (15–30 Hz). Note that the reference signal captures a slow hemodynamic response after the airpuff as well as the heartbeat artifact, which both appear to be absent from the LFP and PV cell membrane voltage traces.
(J) Mean time-dependent wavelet spectrograms showing the locomotor-evoked increase in beta-frequency (15–30 Hz) power in both the LFP (left) and PV cell (middle) recordings but not in the reference signals (right). Vertical dashed lines mark the time of airpuff delivery.
(K) Mean time-dependent magnitudes of the LFP and the two fluorescence signals at high frequencies (15–50 Hz) across 50 trials in one mouse. Vertical dashed line marks the time of airpuff delivery. Shaded area: SEM.
(L) Plots of the mean coherence between the LFP and the two fluorescence signals during resting (dashed curves) and running (solid curves) periods across 36 rest-to-run transitions in one mouse. Shaded area: SEM.
(M–Q) Studies of the joint dynamics of excitatory and inhibitory neuron types in the primary visual cortex of awake mice.
(M) Local injection of three AAVs of the PHP.eB serotype into the visual cortex of VGat-Cre mice allowed expression of Cre-dependent Varnam1 in GABAergic interneurons, Ace-mNeon1 in pyramidal neurons, and cyOFP, a reference fluorophore.
(N) Confocal images of a brain slice from a mouse expressing the fluorescent proteins shown in (M). Scale bar: 50 μm.
(O) Raster plots showing the population membrane voltage dynamics of pyramidal cells (top) and interneurons (bottom) in the same mouse on 96 different trials of visual stimulation. The visual stimuli consistently evoked voltage oscillations in both neural populations. Vertical dashed lines in (O) and (Q) mark the time of visual stimulus onset.
(P) Mean time-dependent fluorescence traces obtained by averaging the signals from all three fluors across all 96 trials of (O). Shaded area: 95% CI.
(Q) Mean time-dependent fluorescence signal magnitudes in the 3–7 Hz (upper) and gamma (30–70 Hz; lower) bands for all three fluors. Shaded area: 95% CI over all 96 trials of (O).

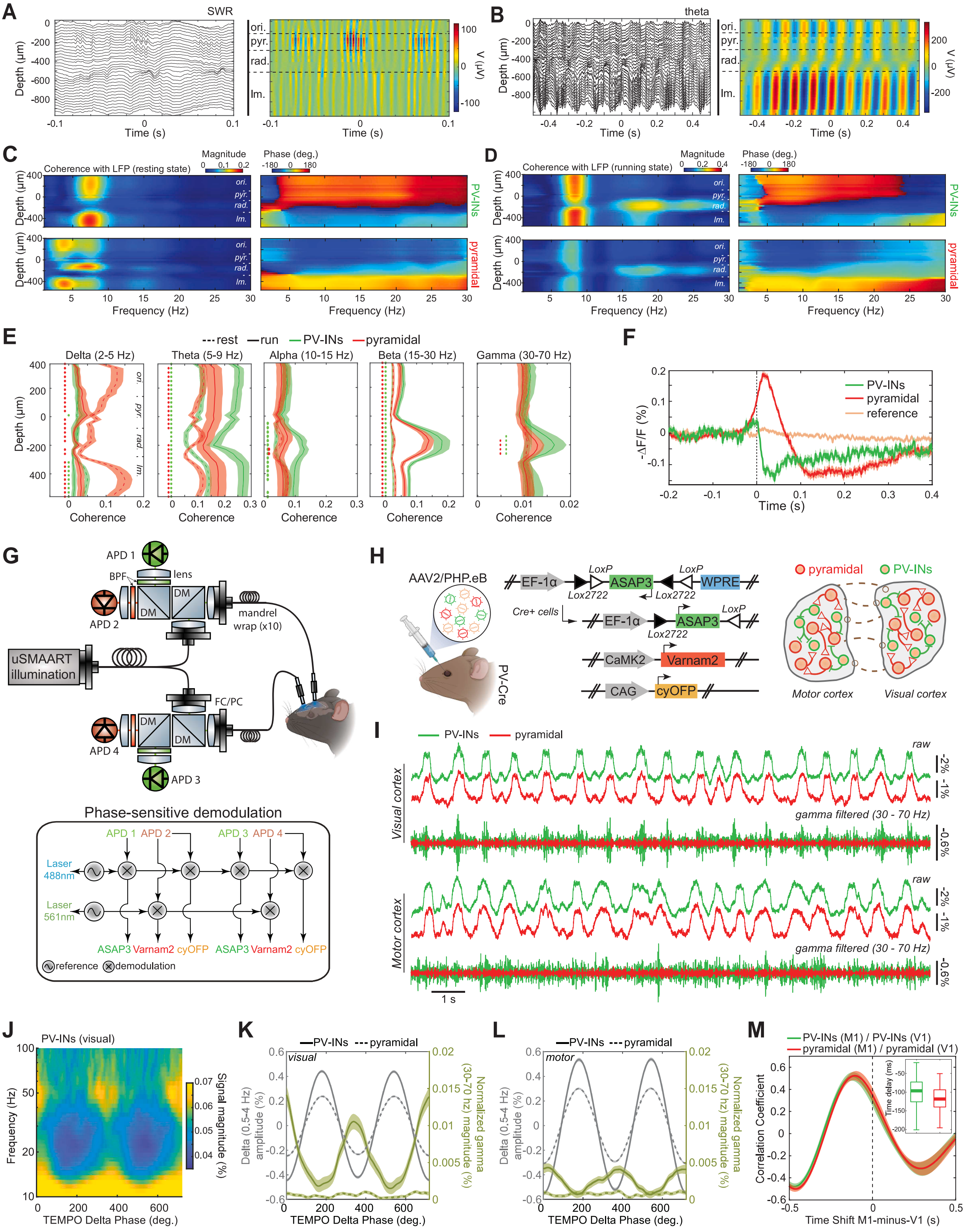

(legend on next page)

Figure S3. uSMAART studies of the dynamic interplay between multiple neuron types, related to Figure 3

(A and B) To identify the boundaries between the different layers of CA1 hippocampus in our 32-site silicon probe recordings, we examined the voltage traces from each of the recording sites during intervals in which there were either sharp-wave ripple (SWR) events or theta oscillations. The left panels of (A) and (B) show illustrative, raw unfiltered LFP traces for each of the 32 recording sites plotted with the tissue depth of each recording noted on the *y* axis, as measured from the most dorsal recording site. An example SWR event is shown in (A), and a bout of theta oscillations is shown in (B). The right panels have color plots of the same data shown at left but temporally bandpass-filtered across either the ripple (120–180 Hz; A) or theta (5–9 Hz; B) frequency range. We identified the 4 canonical hippocampal layers using 3 electrophysiological landmarks: (1) the center of stratum pyramidale corresponds to the depth at which ripple oscillations have the greatest amplitude; (2) the anatomical boundaries of stratum pyramidale were estimated based on the tissue depths at which ripple power approximately vanished; and (3) the anatomical boundary between stratum radiatum and stratum lacunosum moleculare corresponds to the depth at which theta oscillations change in both amplitude and phase.

(C and D) Plots of the magnitude (left columns) and phase (right columns) coherence between PV (top) and pyramidal (bottom) cell fluorescence voltage signals and the LFP across the 32 recording sites of the silicon probe (plotted as a function of depth relative to the center of stratum pyramidale) during resting (C) and running (D) periods. Data are from a different mouse as that in Figures 3N and 3O.

(E) Depth-dependent average coherence magnitude between each of the 32 recording sites of the silicon probe and PV (green curves) and pyramidal (red curves) cell TEMPO signals across 5 different frequency bands as extracted from the data of (C) and (D). During rest (red and green dashed curves), PV and pyramidal cells exhibited delta frequency rhythms that had greater coherence with the LFP than during locomotion (red and green solid curves), especially in stratum oriens and stratum radiatum. During locomotion but not rest, PV and pyramidal cells exhibited beta and gamma frequency rhythms that were coherent with the LFP, especially in stratum pyramidale. Green and red dots indicate tissue depths at which there were statistically significant differences between the coherence values associated with rest and locomotion ($p < 0.01$, two-sample rank-sum test). Averages were performed across $n = 26$ and $n = 41$ epochs (each 30 s in duration) of rest and locomotion, respectively. Shaded areas: 95% CI.

(F) Mean time-dependent traces of the fluorescence voltage signals for CA1 hippocampal PV interneurons and pyramidal cells during ripples as in Figure 3Q but for another mouse. Shading: SEM.

(G–M) uSMAART allows concurrent optical voltage recordings of two neuron types in each of two different brain areas.

(G) Diagrams of the optical pathway (top) and electronic signal demodulation scheme (bottom) used to monitor two neuron types in each of two different areas.

(H) Retro-orbital injection of three PHP.eB viruses into PV-Cre mice enabled expression of Cre-dependent ASAP3 in PV interneurons, Varnam2 in pyramidal neurons, and cyOFP in all neuron types.

(I) Example fluorescence voltage traces for PV and pyramidal cells in visual (V1) and motor (M1) cortical areas of a KX-anesthetized mouse. For each brain area, traces in the top set are unfiltered, whereas traces in the bottom set are bandpass filtered (30–70 Hz). Note that in V1, it is apparent that high-frequency oscillations coincide with up-state depolarization events. Further analyses of this mouse are in (J)–(M).

(J) Wavelet spectrogram of the activity of the PV interneurons in V1 averaged across cycles of the delta oscillation (using 0.9 Hz as the frequency of peak delta power). Bursts of gamma frequency (30–70 Hz) power arose at depolarized voltages of the delta rhythm. In (J)–(L), the convention used for the phase of the delta oscillation is that 0° refers to the trough of the oscillation, i.e., the greatest hyperpolarization in the TEMPO signal.

(K and L) Average signal amplitudes filtered at the delta frequency (black curves, left axis) and the magnitudes of gamma frequency activity (olive curves, right axis) estimated for each cell type in V1 (K) or M1 (L). Note that ASAP3-labeled PV interneurons (solid curves), but not Varnam2-labeled pyramidal neurons (dashed curves), displayed delta-gamma CFC in both areas, with CFC in V1 being stronger than in M1. The observed difference between the two neuron types was likely due to the distinct signaling ranges of the two GEVIs used. Shading: SEM.

(M) Plots of the mean time-dependent cross-correlation coefficient between the two areas V1 and M1 quantified using the voltage dynamics of either excitatory or inhibitory neuron types in each brain area. For PV cells, anesthesia-induced delta rhythms in M1 exhibited a -103 ± 41 ms (mean $\pm$ SD) phase lead over the delta rhythms in V1, whereas in pyramidal cells the phase lead was -117 ± 35 ms. Shading: SEM over 60 samples, each 10 s in duration. Inset: box-and-whisker plot of the differences in the times of the peak correlation coefficient between V1 and M1, for PV (green) and pyramidal (red) cells. Horizontal lines denote medians, boxes cover the middle two quartiles, and whiskers extend to the 10th and 90th percentiles.

CellPress
OPEN ACCESS

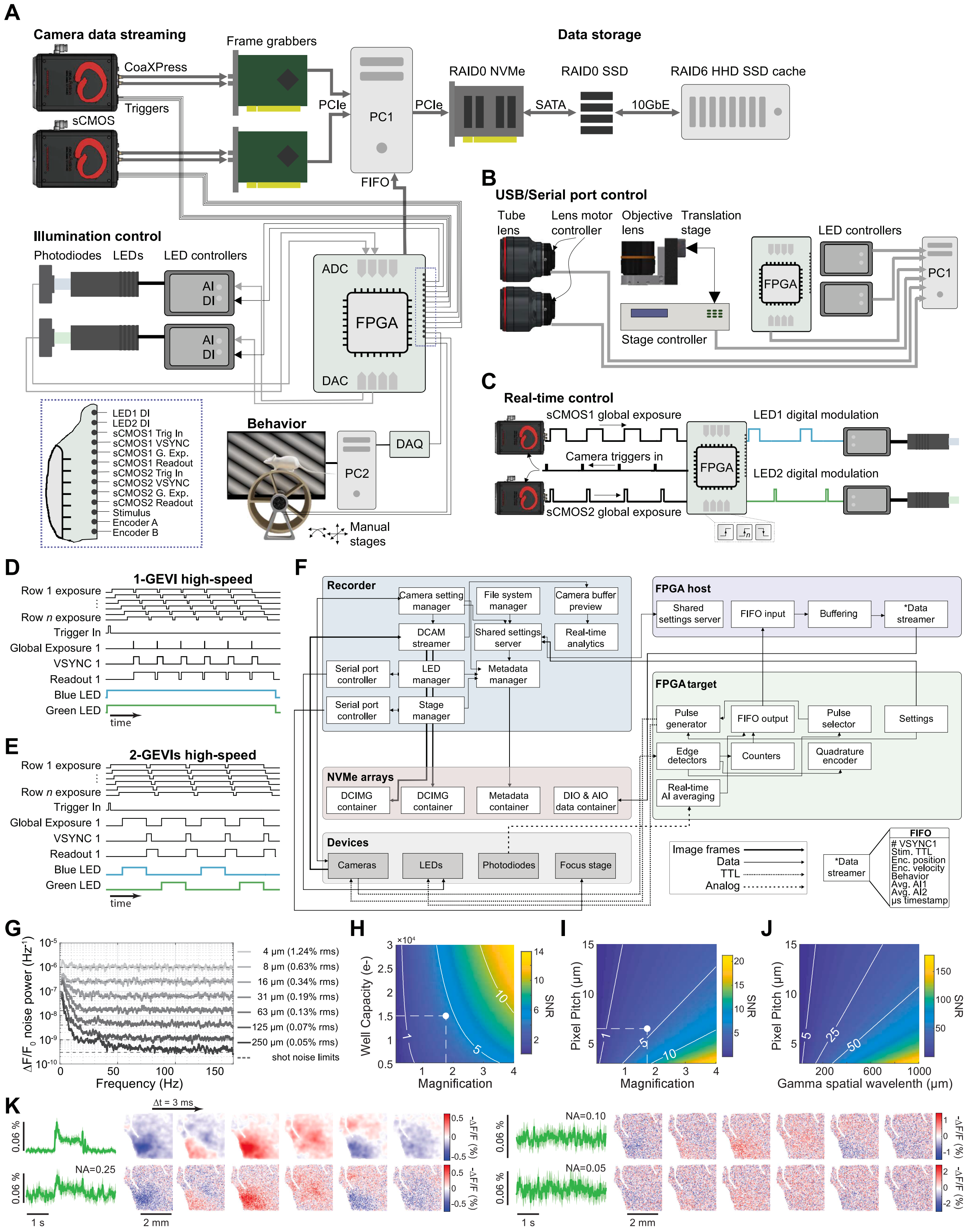

(legend on next page)

Figure S4. Electronics, controllers, and software for the TEMPO mesoscope, related to Figure 4

(A) Each of the two sCMOS cameras (Orca Fusion; Hamamatsu) streams image frame data at 0.8 GB/s via the CoaXPress interface to their respective frame grabbers installed on a single personal computer (PC). A RAID0 hardware array of non-volatile memory express (NVMe) hard drives on PCIe stores incoming frames in real time. For each frame, the field-programmable gate array (FPGA) transmits structured data back to the computer using a FIFO queue framework (see F). After each imaging session, we stored and processed the data on a RAID0 SSD array on the same PC. We backed up the raw data on a RAID6 HDD array connected to the PC via a 10 GbE optical link. We used a FPGA board to control the cameras' input and output triggers as well as the LEDs pulsing protocol (see D and E). Inset: expanded view of all digital signals received or sent by the FPGA board. For dual cell-type mesoscope TEMPO measurements, we monitored the LEDs' powers with photodiodes, whose signals were sent to the analog input ports of the FPGA. For the visual stimulation protocol (Figures 5 and 7), we used another PC with its own DAQ system (see STAR Methods) and recorded the stimulation timing on the FPGA board. We monitored the rotation of the mouse's running wheel using a quadrature rotary encoder whose pair of encoders A and B were routed to the FPGA.

(B) The TEMPO mesoscope uses a combination of USB and serial port communication to control its components from a single PC workstation, which facilitates streamlined operation and synchronization of the entire system. The focusing mechanism in each tube lens (see Figure 4A) is controlled via a serial port, as is the driver for the stage adjusting the axial position of the objective lens. The FPGA board communicates with the main PC via USB. The LED controllers use a serial port.

(C) To achieve uniform illumination and prevent spatially dependent flickering, it is crucial to precisely trigger the LED pulses within the time period when all camera rows are simultaneously illuminated. For dual cell-type TEMPO mesoscope recordings, real-time TTL pulses controlled the LEDs based on the global exposure of the cameras operating in rolling shutter mode. The FPGA board generated a camera trigger and created an interleaved pulse sequence that matched the camera exposure precisely. To ensure uniform illumination, the FPGA board triggered either the green or blue LED, using every other pulse from the global exposure signal provided by the camera.

(D) Triggers and digital pulse sequences for experiments with one GEVI. Both cameras are triggered simultaneously with one TTL pulse and operate in rolling shutter mode, with a frame exposure duration that maximizes the frame acquisition rate for the chosen FOV size and duty ratio of the frame imaging time, i.e., avoiding the sensor reset time. To synchronize the recording on the sub-frame level, we monitor different types of triggers that have different meanings. The camera operates in rolling shutter mode, in which all camera rows are exposed simultaneously for a very short time. To control this precisely, the camera outputs the global exposure trigger signal. The camera outputs are also monitored via the VSYNC and readout triggers. The VSYNC signal indicates the end of a frame and the start of a new one and is vital for achieving accurate synchronization of the data. The two cameras themselves are not directly synchronized to each other, but we routinely use an oscilloscope and logic analyzers to test that no misalignment occurs during the duration of typical recordings (<10 min).

(E) Schematic of the timing protocol used for dual neuron-type recordings. To capture sufficient fluorescence light in this modality, it is crucial to extend the exposure time of both cameras beyond their default values while staying within the global exposure period. However, the trade-off is that longer exposure times lead to a slower frame rate, and we are limited by the energy of the illumination pulse. Thus, careful optimization is needed to find the optimal exposure time that balances the needs for sufficient photon capture and for having a reasonable frame rate. This implies that the frame rate is slower than that allowed in principle by the FOV sampled on the camera chips. Using global exposures of both cameras as depicted in (C), we created interleaved pulses of blue and green LED illumination. The interleaved pulses allowed us to separate the signals from each GEVI using a long Stokes-shift reference fluorophore (cyOFP).

(F) The high-level architecture of software and data flow of the system comprises 3 main programs: the Recorder graphical user interface, FPGA host software, and FPGA target software. The Recorder program acquires, processes, and stores data in real time. The FPGA host software communicates with the FPGA target software on the FPGA board, which controls the devices involved in data transfer and real-time control, such as the cameras, LEDs, photodiodes, and focus stage. We stored data in real time on NVMe arrays, which allowed us to perform continuous recording for nearly 1 h.

(G) To study the effects of spatial averaging on high-frequency noise, we used a wild-type mouse in which GFP and mRuby2 were virally expressed in all neuron types. Through a 7-mm-diameter cranial window, we imaged this mouse at 300 Hz with the TEMPO mesoscope (Figure 4A) and unmixed signals in the green and red-channel movies via convolutional unmixing (Figure S5). Plotted are the power spectral densities of single-pixel noise computed with different levels of spatial averaging. Usually, we averaged voltage and reference signals over 31-μm-wide areas of brain tissue (8×8 camera pixels), as we found this to be an effective compromise that yielded reasonable computational speed, sensitivity to high-frequency phenomenon, and spatial accuracy of the unmixing procedure.

(H–K) We quantified the ability of the TEMPO mesoscope to capture traveling gamma oscillations as a function of different system parameters, such as the camera well capacity, the camera pixel size, and the system magnification. We also considered the spatial wavelength of the high-frequency voltage dynamics as an input parameter.

(H–J) Theoretical estimates of the wave detection SNR, highlighting the influence of different parameters of the optical system and the sCMOS camera chip. The plots show the interactions between three different pairs of parameters: the optical magnification and the camera's pixel well capacity (H); the magnification and the camera pixel pitch (I); or the wavelength of the traveling gamma wave and the pixel pitch (J). With our system's magnification of 1.7$\times$, well capacity of 15,000 electrons, and NA of 0.47, the resulting single-pixel level SNR was about 4.28 (marked by the white points in each panel). This value is sufficient to quantify the dynamics of a single traveling gamma wave, as shown in Figure 5. Jointly, these panels suggest that, to detect high-frequency propagating voltage waves, it is best to use a modest magnification over the brain areas of interest together with an sCMOS camera with a small pixel size and large well capacity.

(K) Detection of propagating gamma events (Figure 5) strongly depends on the voltage signal SNR, which in turn depends on the NA of the microscope objective lens. The NAs considered in this set of plots are 0.5 (the actual NA value of our lens, real data shown in the upper left plots), and then 0.25, 0.1, and 0.05, which respectively led to declines in SNR by factors of 2, 5, and 10. The effects of having different SNRs were mimicked by computationally adding noise to the panels of the top left row (gamma-band [35–100 Hz] filtered images; 3 ms between frames), thereby reducing effective SNR levels.

CellPress
OPEN ACCESS

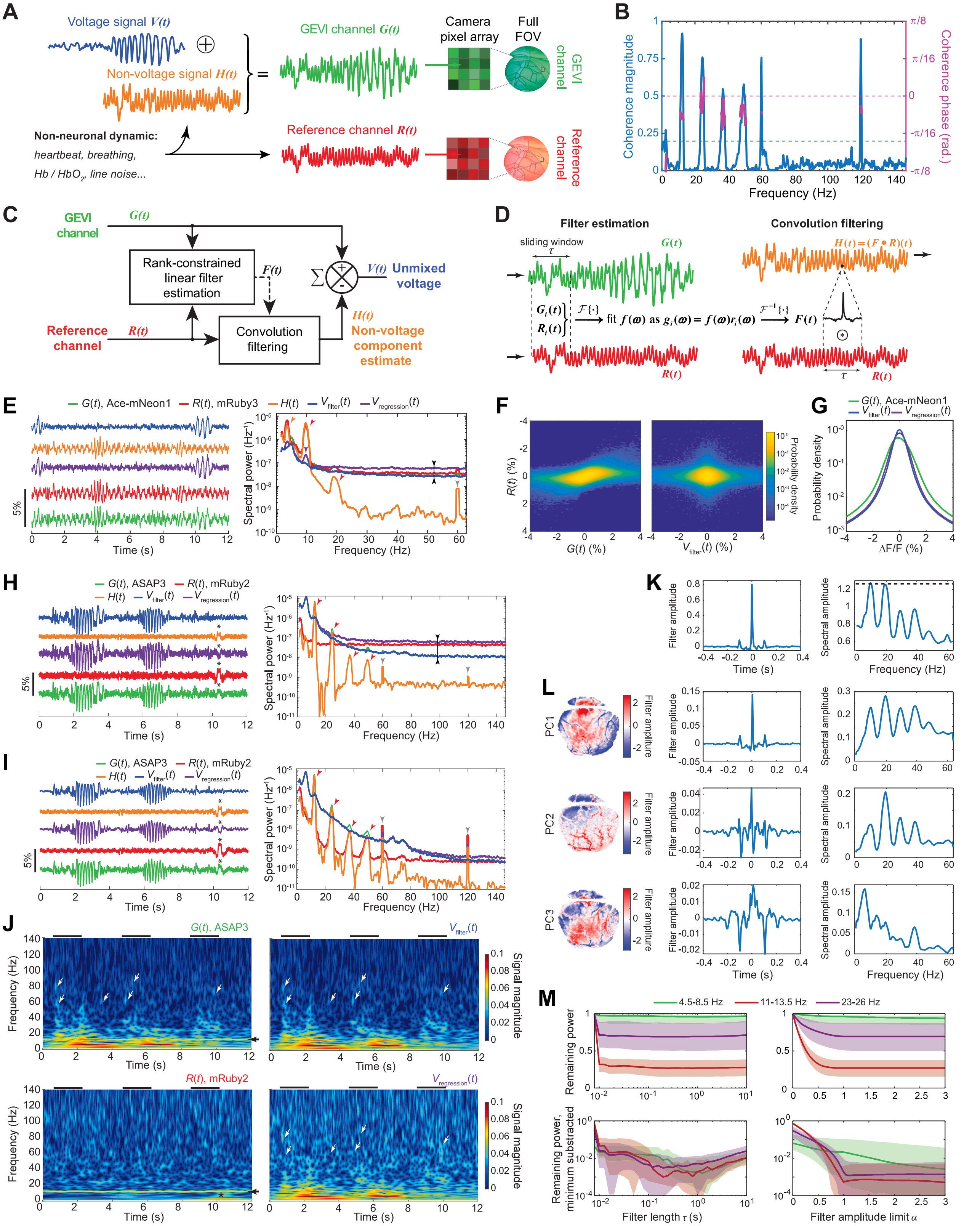

(legend on next page)

Figure S5. Convolutional unmixing removes biological and instrumentation artifacts from neural voltage signals in a frequency-dependent manner, related to Figure 4

(A) A model of signal content used for the estimation and unmixing of artifacts from the GEVI fluorescence channel. Hemodynamics and other artifacts are present in both fluorescence channels, but neural voltage signals are present only in the fluorescence voltage channel. The diagram refers to studies with a single GEVI; when using two GEVIs, we unmixed the reference channel content from each voltage channel independently.

(B) Plots of the frequency-dependent normalized amplitude (blue curve) and phase (magenta curve) of the coherence between the voltage and reference fluorescence channels before unmixing and averaged over brain area V1 in an example mouse expressing ASAP3 in PV interneurons and mRuby2 in all neuron types (same mouse as H–J and M). Phase values are shown only for frequencies at which the coherence amplitude is >0.2 (horizontal dashed blue line). The two fluorescence channels display high coherence peaks at the fundamental (~12 Hz) and harmonic frequencies of the heartbeat, as well as the fundamental (60 Hz) and second-harmonic (120 Hz) frequencies of the electric power line noise. Although these stereotypical oscillations are highly coherent between the GEVI and reference channels, the phases are variable and differ from zero (dashed purple line), and a simple linear regression approach is unable to capture non-uniform phase lags.

(C) Block diagram of the unmixing strategy. A rank-constrained linear filter, $F(t)$, is estimated using both fluorescence channels and captures the manner in which artifacts seen in the reference channel coherently invade the voltage channel in different frequency bands (e.g., as in B). $F(t)$ is convolved with the reference channel, $R(t)$, to attain an estimate, $H(t)$, of the non-voltage signals present in the GEVI channel. $H(t)$ is subtracted from the GEVI channel trace, $G(t)$, to attain an estimate of the true voltage signals, $V(t)$. Plain arrows represent data flow. Dashed arrow denotes transfer of the $F(t)$ function.

(D) Schematic of the filter estimation and convolution steps in (C) (see also STAR Methods). We used the Fourier transform $\mathcal{F}\{\}$to obtain the frequency-domain representations, $r(\omega)$ and $g(\omega)$, of $R(t)$ and $G(t)$, respectively, over sliding time windows of duration, τ (typically 0.5–2 s, see M). We then used linear regression to compute for each frequency, ω, the value of the filter coefficient, $f(\omega)$, that best describes the amplitude with which the reference signal, $r(\omega)$, is present in the voltage channel, $g(\omega)$, across the set of all time windows. After performing an inverse Fourier transform of $f(\omega)$ to obtain the filter's time-domain representation, $F(t)$, we estimated $H(t)$ by convolving $F(t)$ with $R(t)$ and then subtracted $H(t)$ from $G(t)$ to obtain $V(t)$.

(E) Left: an example set of raw, $G(t)$ and $R(t)$, and unmixed, $V_{filter}(t)$ and $V_{regression}(t)$, fluorescence $\Delta F/F$ traces for the Ace-mNeon1 voltage indicator and mRuby3 reference fluor obtained from an Ai218 transgenic mouse crossed with a PV-Cre mouse (Figure S6). Traces are colored to match the convention used in (A)–(D) and $R(t)$ was scaled (by 1.8-fold) to match the power at the heartbeat frequency (that is the dominant shared contribution, ~10 Hz) between $G(t)$ and $R(t)$. Hemodynamics are visible in $R(t)$ (red trace) throughout the recording segment and contaminate the raw voltage trace (green) but are reduced after unmixing using either standard linear regression[131] (purple trace) or our filter-based approach (blue trace). Notable voltage signals appear just after ~10 s and remain after unmixing. Right: power spectral density plots for all 5 traces shown in the left graph, computed across the full recording duration. After unmixing using our filter-based method, the voltage signals (blue curve) lack several spectral peaks that arise from heartbeat-related artifacts (~9 and ~18 Hz harmonic; red arrowheads), breathing-related artifacts (~4 Hz; orange arrowhead), and electric line noise (60 Hz; light gray arrowhead). Unmixing with a standard linear regression (purple curve) does not fully remove the heartbeat and breathing artifacts, since a standard regression does not account for amplitude and phase differences in the heatbeat content between the two fluorescence channels (see B). Moreover, the linear regression introduces high-frequency (>10 Hz) noise from the reference channel into the voltage channel, as seen via the increase in baseline high-frequency spectral power after unmixing (marked with opposing black arrowheads). By comparison, after the filter-based unmixing approach, the power at high frequencies remains unchanged after unmixing. Further analyses of the data from this mouse are shown in (F), (K), and (L).

(F) Density maps showing the joint probability distribution of fluorescence signals in the voltage and reference channels before (left) and after (right) filter-based unmixing across the full recording session (9 min) of (E). After unmixing, the two channels are less correlated.

(G) Probability distributions of signal fluctuations for the traces used in (F), confirming that filter-based unmixing reduces the variance level in the voltage trace, $V_{filter}(t)$. Note that unmixed traces $V_{filter}(t)$ and $V_{regression}(t)$ have smaller variances as compared with the raw trace $G(t)$.

(H and I) Plots in the same format as those in (E) from studies of a mouse expressing the ASAP3 voltage indicator as computed for a single image pixel in area V1 (H) or averaged over the entirety of V1 (I). Colored and black arrowheads mark the same spectral features noted in (E). Asterisks mark an artifact at ~10.5 s that is likely to result from brain motion. Further analyses of this mouse are shown in (J) and (M).

(J) Wavelet spectrograms of the traces $G(t)$ and $R(t)$ from (I) (left column) and of the estimated traces of the voltage signal obtained by standard regression, $V_{regression}(t)$ (lower right), or convolutional filtering, $V_{filter}(t)$ (upper right), estimated for a single image pixel in area V1. Black horizontal bars above the plots mark periods of visual stimulation. Both unmixing approaches (right column) removed the heartbeat artifact and its harmonic (~11 and 22 Hz; marked with black arrowheads), but only convolution filtering removed a low-frequency artifact (at time ~10.5 s and frequency <5 Hz, marked with a black asterisk as in H and I), which is likely to result from brain motion. Notably, because linear regression adds high-frequency noise to the estimated voltage trace, only convolution filtering preserves the visibility of visually evoked high-frequency voltage transients (marked with white arrowheads) that were absent in the reference channel (bottom left).

(K) Plots of the filter, $F(t)$, in the time (left) and frequency (right) domains averaged across the whole imaging window, for the example mouse of (E). The horizontal dashed line in the right plot represents the unmixing coefficient as determined by linear regression estimated on the fundamental frequency of the heartbeat artifacts.

(L) To characterize how $F(t)$ varied across the cortical surface, we performed a principal component analysis (PCA) for the example mouse used in (E). Plots show the spatial variations (left column), time dependence (middle column) and frequency-domain power density (right column) for the first three principal components (PCs) of $F(t)$. The first PC is mainly uniform across the FOV, except for the periphery. The second and third PCs capture a gradient of variation across the FOV and distinct sets of blood vessels. The PCs have distinct signatures in the frequency domain, highlighting their distinct hemodynamic properties.

(M) Plots of the relative signal powers that remain after unmixing as a function of the hyperparameters τ (filter length; left column) and α (filter amplitude limit; right column) in a given frequency band. The dependencies are plotted for 3 frequency ranges: low-frequency visually evoked voltage signals (4.5–8.5 Hz), the fundamental (11–13.5 Hz), and first harmonic (23–26 frequencies Hz) of the heartbeat. For individual pixels in the GEVI channel $G(t)$, we computed the power in each of the frequency bands as a function of both hyperparameters, either by normalizing by their respective initial values (top row) or by normalizing and then subtracting each pixel's minimum value (bottom row). The solid curves show median values across all pixels for every value of the hyperparameter, and the shaded areas indicate the 0.05–0.95 interquartile range. In each case, we first estimated the convolutional filter $F(t)$ on a training dataset and then applied it to perform unmixing on the testing dataset (50/50 split between train and test). Note that, in the bottom right plot, the heartbeat-related powers decline rapidly until $\alpha \sim 1$ and stay constant for higher values of α. However, the power in the voltage frequency band flattens for $\alpha \sim 1$ but then decreases for higher values, suggesting over-subtraction of the GEVI signals due to excessive amplification of the filter's coefficients at this specific frequency band due to cross-channel coherence. Typical values of τ are $\tau \sim 0.5 - 2$ s, depending on the dataset. We used $\tau = 1$ s for the plots in the right column. A typical value for α is 1.2, meaning that no coefficient values of the convolution filter $f(\omega)$ will be more than 20% greater than the linear regression coefficient (e.g., 1.25 in K) for the fundamental hemodynamic frequency. The dataset used is the same as that in (H) and (I).

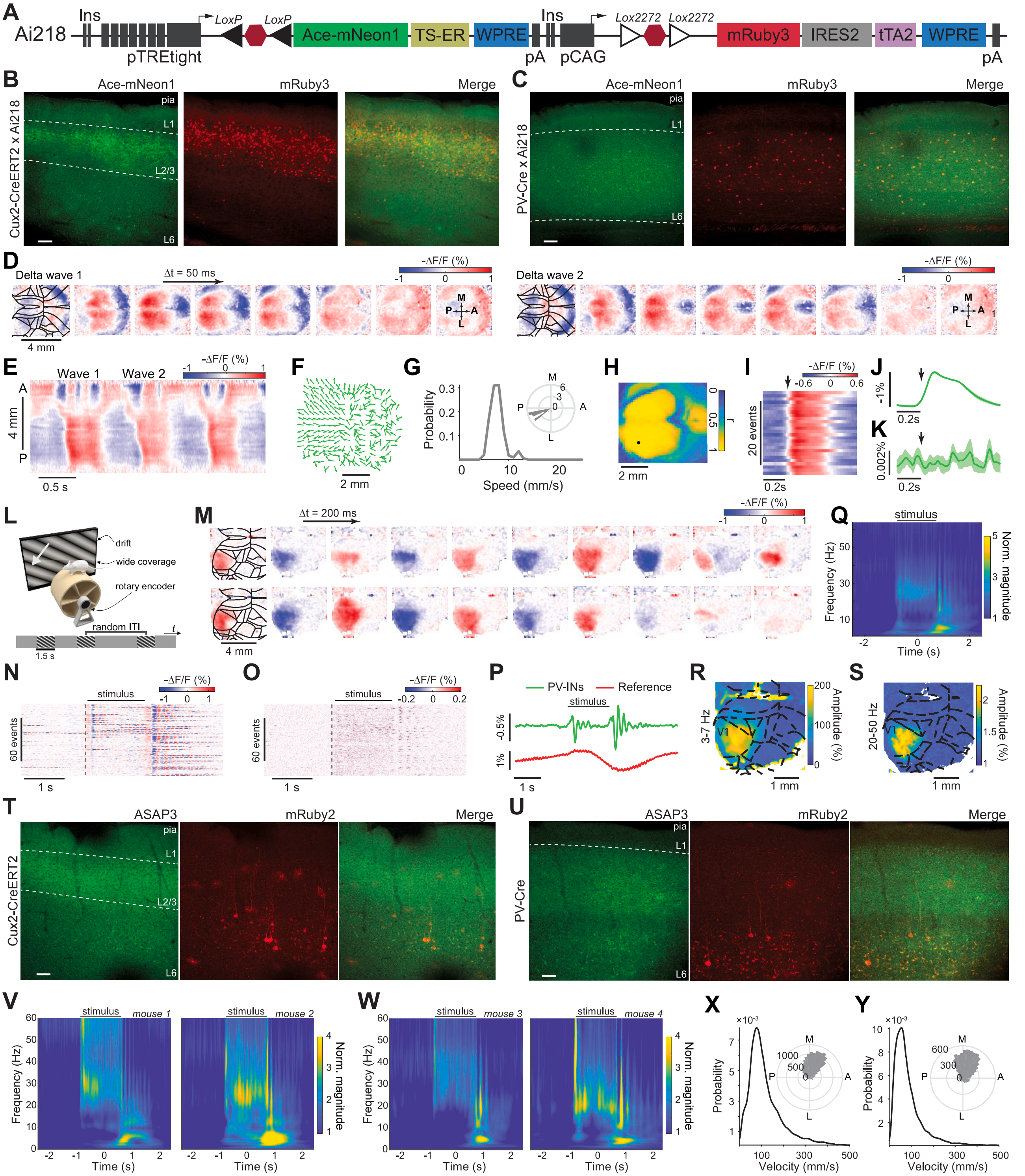

Figure S6. Construction and validation of mouse transgenic and viral methods for fluorescence labeling expressly designed for TEMPO studies, related to Figures 4 and 5

(A) To co-express the FRET-opsin voltage sensor Ace-mNeon1 and the reference fluor mRuby3 in specific cell types, we generated a Cre-dependent Ai218 reporter mouse line expressly designed for TEMPO studies (see STAR Methods). The genetic construct is inserted in the TIGRE2.0 mouse locus. To express Ace-mNeon1 and mRuby3 in targeted neuron types, we crossed the Ai218 reporter mice with cell-type-specific Cre-driver lines.

(legend continued on next page)

(B and C) Confocal fluorescence images of a pair of brain slices showing the cortical expression patterns of Ace-mNeon1 and mRuby3 in two mice that were genetic crosses of Ai218 with either a Cux2-Cre^{ERT2} mouse line (B) to label L2/3 pyramidal cells or a PV-Cre line (C) to label PV interneurons. Approximate neocortical layers are marked to aid visualization (L1, layer 1; L2/3, layer 2/3; and L6, layer 6). Scale bars: 100 μm.
(D) Two examples of traveling neocortical voltage waves in the delta frequency band shown in sequences of Ace-mNeon1 images (50 ms between successive images) taken at 130 fps in a KX-anesthetized Ai218 × Cux2-Cre^{ERT2} mouse. Images underwent unmixing (Figure S5) to remove hemodynamic changes captured in the mRuby3 channel but were not otherwise filtered. Brain area boundaries are superposed on the first image in each sequence. In each case, a voltage depolarization (denoted by red hues) sweeps across the cortex in the anterior to posterior direction. For display purposes only, the images shown were spatially low-pass-filtered using a Gaussian filter (156 μm FWHM). Further analyses of waves in this animal are in (E)–(K).
(E) Color plot (top) showing the anterior to posterior propagation of the two traveling waves in (D). To create the plot, at each time point (*x* axis) and for each A-P coordinate (*y* axis), we averaged the measured fluorescence ΔF/F values along the medio-lateral direction.
(F) Flow maps showing the local propagation directions of voltage depolarization for the individual delta wave #1 observed in (D) and (E). Flow vectors are all normalized to have the same length.
(G) Distributions of delta wave propagation speed across all delta events seen in the Ai218 × Cux2-Cre^{ERT2} mouse computed near the center of area V1 (marked by a black dot in H), where wave propagation was consistently anterior to posterior. Inset: polar histogram showing the distribution of wave propagation direction for the same mouse at the same location, revealing an approximate alignment of wave propagation with the A-P axis.
(H) Maps of peak correlation coefficients, *r*, for a Ai218 × PV-Cre mouse computed for each spatial point by calculating the temporal correlation function between the local fluorescence trace and that at the center of V1 (black dot) and then finding this function's maximum value.
(I–K) Raster plots of down-to-up transition events in the ΔF/F voltage signal in area V1 (I) along with plots of the mean time-dependent activity obtained by averaging over 29 transition events in the delta frequency (0.5–4 Hz) (J) or gamma frequency (30–60 Hz) bands (K). At the peaks of the delta waves, we observed no activity in the gamma frequency band, unlike in our studies of anesthetized mice using the ASAP3 indicator. (However, see O below.) The dark arrows mark the onset of the down-to-up state transition. Shading in (J) and (K): 95% CI.
(L) We used the same visual stimulation approach as in Figure 3B.
(M) Two example sequences of fluorescence images from a Ai218 × PV-Cre mouse showcasing the spatiotemporal dynamics of visually evoked 3–7 Hz oscillations in PV interneurons in area V1. Successive images shown in the sequences were taken 200 ms apart. Brain area boundaries, based on the Allen Brain Atlas (Figure 4A, inset), are superposed onto the first image in each sequence. For display purposes only, the images shown were spatially low-pass-filtered using a Gaussian filter (156 μm FWHM). Further analyses of the waves detected in this animal are in (N)–(S).
(N) Raster color plots showing fluorescence voltage signals averaged over V1, revealing 3–7 Hz oscillations locked to stimulus offset in V1 (80 stimulus trials shown).
(O) Raster plots of gamma-band filtered fluorescence voltage activity in V1, showing that gamma-band activity was evoked during stimulus presentation in V1 (80 stimulus trials shown).
(P) Mean time-dependent traces of PV interneuron voltage activity (green trace) and reference fluor signals (red trace) obtained by averaging the unfiltered fluorescence signals of (N) over all 80 trials. Shaded area: SEM.
(Q) Stimulus-triggered average wavelet spectrogram of the PV cell fluorescence voltage traces from (N) and (P), revealing the high-frequency voltage activity during visual stimulus presentation, plus the 3–7 Hz oscillations that arose at stimulus offset.
(R and S) Spatial maps showing the magnitudes of visually evoked 3–7 Hz (R) and gamma-band (S) activity at their peaks within the averaged trial. The plots illustrate that the visually evoked voltage signal was confined within the visual system.
(T and U) Fluorescence confocal imaging of brain slices from a Cux2-Cre^{ERT2} mouse that virally expressed mRuby2 in all cell types and ASAP3 in L2/3 pyramidal neurons (T) and a PV-Cre mouse that virally expressed mRuby2 in all cell types and ASAP3 in PV interneurons (U). Images were acquired from the sensory neocortex atop the dorsal hippocampus. The mouse preparations are the same as for Figures 4 and 5. L1, layer 1; L2/3, layer 2/3; L6, layer 6. Scale bars: 100 μm.
(V and W) Event-related wavelet spectrograms for the two PV-Cre mice (V) and two Cux2-Cre^{ERT2} mice (W) used in Figures 5G and 5H.
(X and Y) Probability distributions of 3–7 Hz wave speed and histograms of propagation direction (insets) in area V1 for a PV-Cre mouse (mouse 1 in Figures 5G and 5H) determined from either the raw data (X) or by computing wave speeds using gamma-band filtered (35–100 Hz) versions of the same movie data (Y; $n = 50$ wave events). The similar speeds and directions evidenced in the two sets of graphs suggest that the high- and low-frequency content are both part of the same phenomenon, i.e., with the gamma frequency activity constituting harmonics of a 3–7 Hz carrier wave. Importantly, the speeds and directions of the gamma waves analyzed here are notably distinct from those of the isolated gamma waves shown in Figure 5.

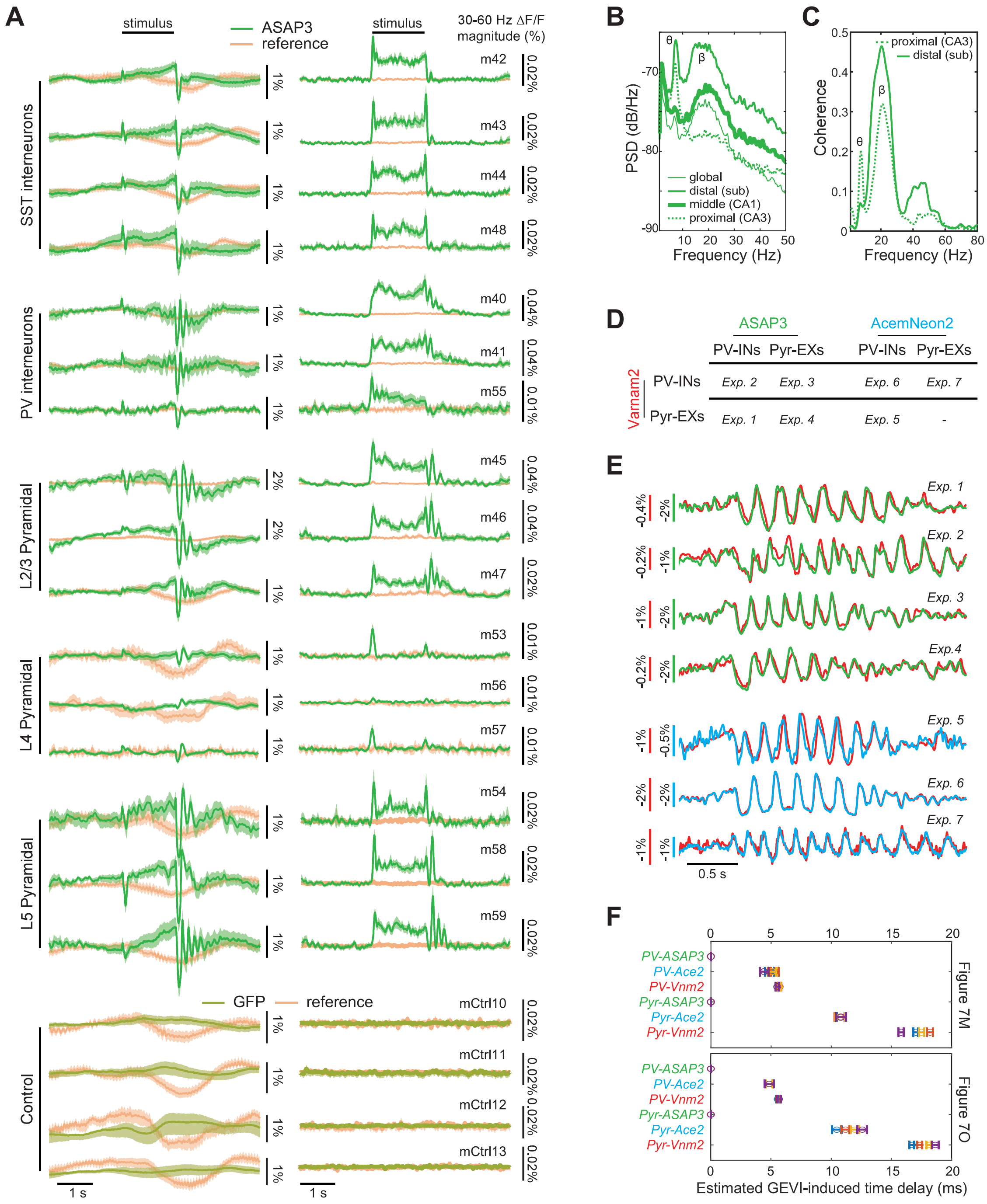

Figure S7. Voltage signal variations with neuron class and indicator type, related to Figures 5, 6, and 7

(A) Similar to Figures 5G and 5H, plotted for mice in which ASAP3 labeled either neocortical SST-interneurons (4 mice), PV interneurons (3 mice), layer 2/3 pyramidal neurons (3 mice), layer 4 pyramidal neurons (3 mice), or layer 5 pyramidal neurons (3 mice). Mean fluorescence traces are in the left plots; mean fluorescence signal magnitudes in the gamma-band (30–60 Hz) are in the right plots. Visually evoked gamma- and 3–7-Hz-band activity are respectively visible during stimulus presentation (black horizontal bars) and following stimulus offset to varying extents across the cell types. Note, for example, the near absence of oscillatory activity except near the times of stimulus onset and offset. Traces for the mRuby2 reference fluor are included in all plots. The bottom 4 plots show results from control mice, in which the static green fluorescent marker, GFP, was used instead of the green ASAP3 voltage indicator. We did not separate waves between periods of resting and running because consistent with past work,[56] neither occurrence rates (9.1 ± 0.8 vs. 6.8 ± 1.2 oscillation events per min; $p = 0.22$) nor magnitudes (2.8 ± 0.1 vs. 2.7 ± 0.1 Z-scored wave amplitudes relative to baseline values; $p = 0.47$) of visually evoked 3–7 Hz oscillations varied significantly between resting and running states (Wilcoxon signed-rank tests; $n = 7$ mice). Vertical scale bars for the fluorescence traces in the left column indicate the percentage magnitudes but not the signs of the fluorescence changes in both the ASAP3 and reference traces. Because ASAP3 is a negative-polarity GEVI,

(legend continued on next page)

decreases in green fluorescence, corresponding to transmembrane depolarizations, are plotted as upward deflections of the traces, whereas fluorescence changes in the reference channel are plotted with the normal sign convention. The GFP traces are plotted with the same sign convention as the ASAP3 traces. Shading: 95% CI.

(B) Plots of the power spectral density of PV cell fluorescence voltage signals acquired as in Figures 6B–6D by TEMPO imaging in hippocampus at 3 different parts of the 2.1-mm-diameter FOV (proximal, middle, and distal) along the CA1-CA3 axis or across the full FOV (global).

(C) Plots of the coherence between the LFP acquired in the same mouse as in (B) at the CA1 side of the FOV and PV cell voltages at proximal and distal positions in the visible field in area CA1.

(D and E) Table (D) summarizing the voltage-indicator labeling configurations for 7 different experiments (E) that used fiber-optic TEMPO to study relative time delays in the visually evoked 3–7 Hz oscillations of PV interneurons and/or pyramidal neurons in neocortical area V1 (cf. Figures 7M and 7O). Representative voltage traces from each experiment (E) show subtle differences in the measured temporal shifts across the sets of paired traces that depended upon the assignments of 3 different voltage indicators (Ace-mNeon2, ASAP3, and Varnam2) to the 2 neuron classes. Traces are colored according to the color scheme shown in (D).

(F) We determined the temporal shifts in the fluorescence reports of oscillatory activity that arise from voltage-indicator kinetics by performing parametric fits to the values of the time delays found in the data from Figures 7M–7P and in the 7 experiments of (D) and (E) (see STAR Methods). The plots show the mean ± SD temporal shifts induced by each of the 6 possible assignments of the 3 indicators to the 2 neuron classes. The top and bottom plots show time shift values determined using the data from the 4 imaging sessions of Figures 7M and 7O, respectively, which had reversed labeling assignments. The colors of each data point match those used to label the 8 corresponding imaging sessions (n = 7 mice) in each of Figures 7M and 7O. Notably, the estimated time shifts arising from indicator kinetics are nearly invariant across the top and bottom plots, arguing that the values obtained do not vary much across mice. We estimated SD values across 100 different samplings of the data, each containing 100 different oscillation events.
